# Structure of the pre-initiation complex explains CMGE biogenesis

**DOI:** 10.1038/s41586-026-10657-7

**Published:** 2026-06-17

**Authors:** Thomas Pühringer, Berta Canal, Giacomo Palm, Agata Butryn, Emma C. Couves, Oliver Willhoft, Jacob S. Lewis, John F. X. Diffley, Alessandro Costa

**Affiliations:** 1 Macromolecular Machines Laboratory, Francis Crick Institute, London, UK; 2 Chromosome Replication Laboratory, Francis Crick Institute, London, UK; 3 Present Address: Molecular Horizons, University of Wollongong, Wollongong, New South Wales Australia

**Keywords:** Cryoelectron microscopy, Origin firing

## Abstract

When cells enter S phase, bidirectional DNA replication is initiated through the kinase-regulated recruitment of three activators (Cdc45, GINS and Pol ε) to a duplex-DNA-loaded double hexamer of minichromosome maintenance (MCM) ATPases. Together, these proteins form two CMGE helicases that establish divergent replication forks as they become separated^1^. Here, to gain an understanding of CMGE biogenesis, we reconstituted the pre-initiation complex with purified yeast proteins. The cryo-electron-microscopy structure shows a set of firing factors caught in the act of assembling two symmetrical CMGEs. We show how stepwise complex formation reshapes MCM in preparation for DNA opening, and we explain how ATP promotes firing-factor ejection and CMGE maturation. We find that although Sld2 facilitates the recruitment of GINS to MCM, as expected, it also aids the efficient separation of the CMGE dimer, and is essential for the ejection of the lagging strand from MCM. These findings have direct implications for our understanding of the metazoan Sld2 orthologue, RECQL4, and point to a replication-fork establishment mechanism that is conserved across eukaryotes.

## Abstract (web summary)

Cryo-electron microscopy and biochemical reconstitution experiments in yeast provide insight into the assembly of the CMGE complex, a helicase that establishes bidirectional DNA replication in eukaryotic cells, and elucidate the role of the firing factor Sld2.

## Main

DNA replication must occur only once per cell cycle to maintain genome stability. To achieve this, eukaryotes have evolved to temporally separate the loading of the replicative helicase from its activation^1^. During the G1 phase of the cell cycle, two copies of the MCM motor of the replicative helicase are loaded as an inactive double hexamer (DH) onto DNA replication origins^2,3^. Activation occurs after S-phase transition and involves the recruitment of Cdc45 and the tetrameric GINS (Go-Ichi-Ni-San) complex to MCM, together forming the Cdc45–MCM–GINS (CMG) helicase^4,5^. CMG assembly occurs under the control of three kinases. It is promoted by the Dbf4-dependent kinase (DDK)^6–8^ and Cdc28–Clb5 (hereafter, CDK), the activity of which increases when cells enter S phase^9–11^. CMG assembly is inhibited by the checkpoint kinase Rad53, which blocks late origin firing if DNA damage is detected^9–13^. DDK selectively phosphorylates DNA-loaded DHs^14^. A heterodimeric firing factor composed of Sld3 (essential) and Sld7 (dispensable) then recognizes the phosphorylated DH and recruits Cdc45 to MCM (refs. ^10,11,15^). An N-terminal truncation of Mcm4 bypasses the requirement for DDK in cells^7,8^ and cryo-electron microscopy (cryo-EM) work has shown that phosphorylation causes N-terminal Mcm4 to become unstructured^16,17^. Whether Sld3 engages an epitope that is unmasked after N-terminal Mcm4 phosphorylation or whether it merely reads phosphorylated (phospho-) Mcm4 sites remains to be established. Also, although we know that Rad53 prevents CMG formation by targeting Sld3 (alongside DDK)^13^, the mechanism is unclear. The second activating kinase, CDK, targets two firing factors, Sld3 and Sld2. The latter was previously implicated in GINS recruitment. Phospho-Sld2 and phospho-Sld3 are recognized by the Dpb11 ‘phospho-reader’^10,11^. The leading-strand polymerase Pol ε (formed of Pol2, Dpb2, Dpb3 and Dpb4) also contributes to CMG assembly and becomes part of the holohelicase, CMGE. In particular, the N-terminal domain of Dpb2 supports CMG formation in cells^18^, and a complex containing Dpb2 and the C-terminal half of Pol2 achieves GINS recruitment and replication initiation in a reaction reconstituted with purified yeast proteins^19^.

**Fig. 1:**
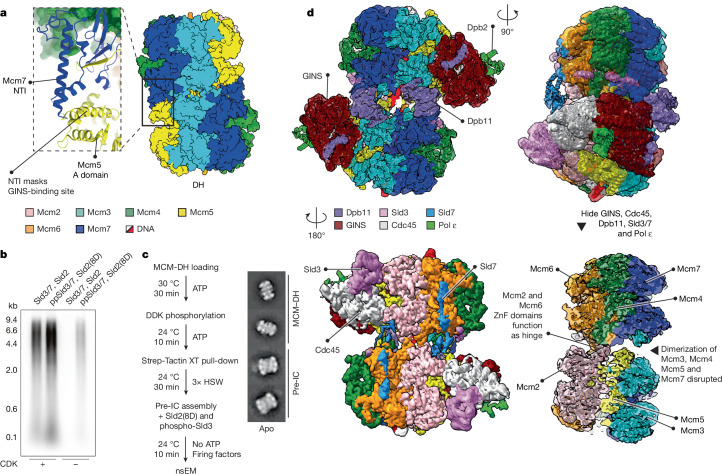
Structures of the double hexamer and the pre-IC. **a**, In the double hexamer (DH; Protein Data Bank (PDB) ID: 7P30), the Mcm7 NTI element from one MCM ring protects the N-terminal Mcm5 A domain of the opposed ring. **b**, CDK-prephosphorylated Sld3/7 (ppSld3/7) and the Sld2(8D) phosphomimetic mutant support origin-dependent replication reconstituted in a test-tube, albeit at reduced levels (experiment performed twice). The low levels could be due either to the Sld2(8D) phosphomimetic mutations covering only a subset of CDK sites, or to inefficient phosphorylation of Sld3 in the isolated prephosphorylation reaction, compared with the complete initiation mix. For gel source data, see Supplementary Fig. 1. **c**, CMG assembly in the absence of ATP, using ppSld3/7 and Sld2(8D), enables formation of the pre-IC. HSW, high-salt wash. Right, representative 2D classes. **d**, Cryo-EM structure of the pre-IC. The two MCMs are split on one side, with Mcm2 and Mcm6 functioning as a hinge. ZnF, zinc finger domain.

In the inactive DH, an Mcm7-specific N-terminal insertion (NTI) from one MCM hexamer reaches across the DH interface and protects the N-terminal A domain of Mcm5 of the opposing hexamer^20^ (Fig. 1a). The same Mcm5 A-domain site is engaged by GINS in the CMG, implying that Mcm7 must let go of Mcm5 for GINS to bind^21,22^. How this happens is unknown. Likewise, it is established that Cdc45 and GINS recruitment occur sequentially^23–25^. Whether recruitment of each component involves only one MCM hexamer or both MCM hexamers at once is debated^25–27^.

Origin activation is controlled by ATP binding and hydrolysis. MCM loading, for example, requires ATP hydrolysis^28,29^, so that, by the time a DH is formed, eight of its twelve subunits are bound to ADP (ref. ^14^). Firing-factor recruitment to the DH promotes ADP release, and ATP binding by MCM achieves stable double CMG–Pol ε (ATP–dCMGE) complex formation, which nucleates DNA melting^24,30^. It has been postulated that a pre-initiation complex (pre-IC) exists^31^, in which Sld2, Sld3–Sld7 (hereafter, Sld3/7) and Dpb11 all bind to the DH at the same time, while also recruiting Cdc45, GINS and Pol ε (refs. ^24,32^). However, the pre-IC has not so far been isolated, and we do not know how Sld2, Sld3/7 and Dpb11 are ejected to achieve ATP–dCMGE maturation^24,30^. Finally, whether the role of Sld2 is conserved across eukaryotes is unclear. In fact, Sld2 functions in CMGE assembly in yeast, whereas the metazoan orthologue, RecQL4, is involved in a loosely defined downstream activation step. To address these issues and understand replisome biogenesis, we took a biochemical reconstitution approach, combined with cryo-EM imaging and single-particle reconstruction.

## Cryo-EM structure of the pre-IC

**Extended Data Fig. 1:**
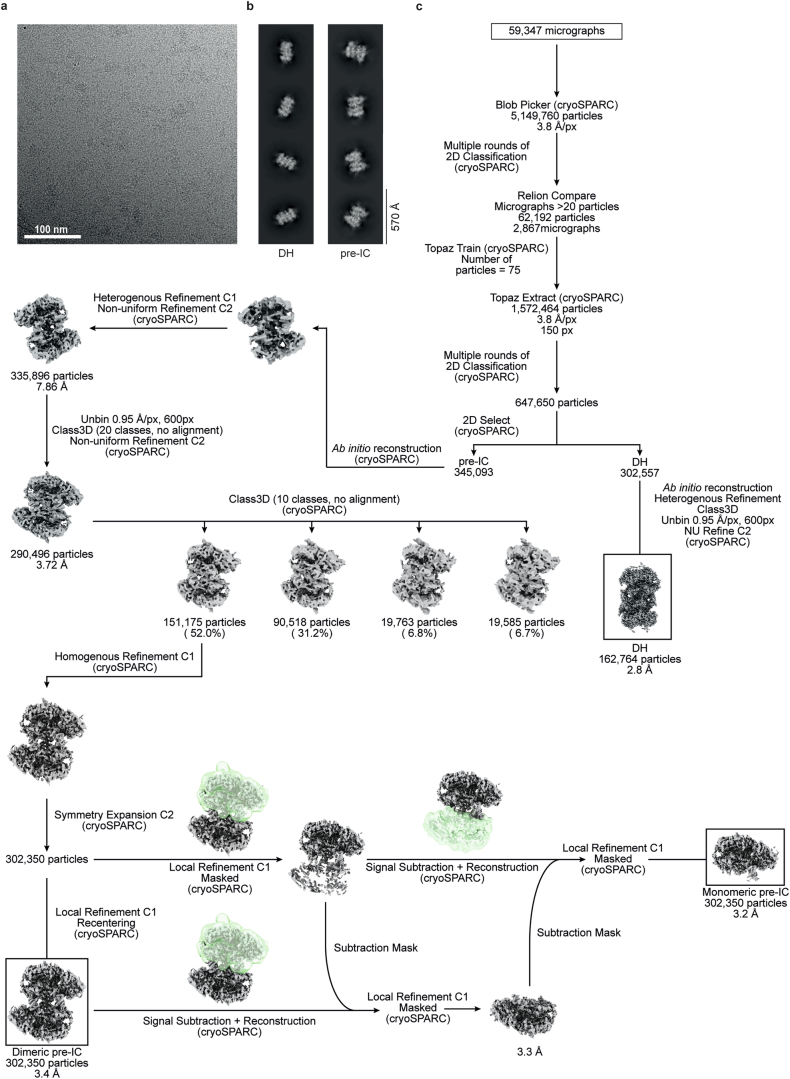
Cryo-EM imaging and image-processing workflow for the pre-IC. **a**, Representative cryo-electron micrograph. This cryo-EM sample was prepared and imaged more than three times. **b**, Cryo-EM 2D averages of the DH and pre-IC assembly. **c**, Image-processing pipeline.

**Extended Data Fig. 2:**
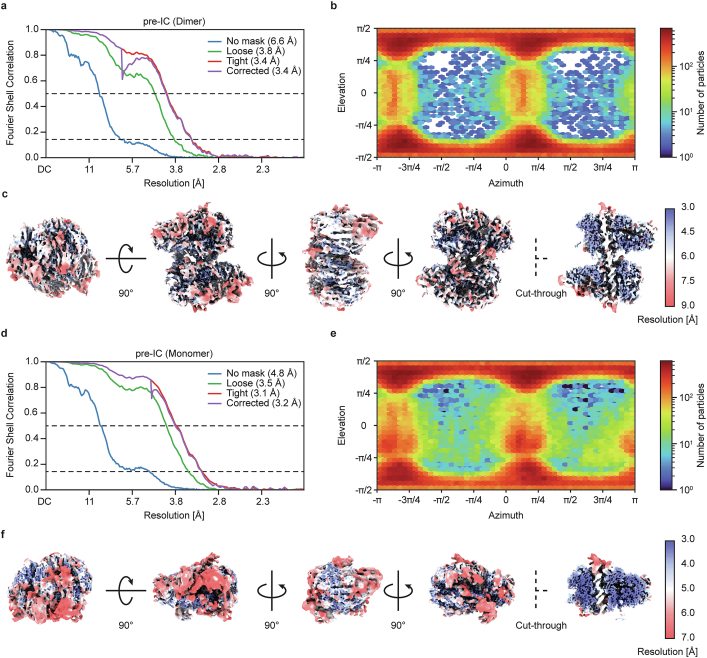
Validation of the pre-IC map. **a**, Fourier shell correlation of the full dimeric pre-IC map. **b**, Angular distribution plot of the full dimeric pre-IC map. **c**, Five views of the of the full dimeric pre-IC map coloured according to the local resolution. **d**, Fourier shell correlation of the signal-subtracted, monomeric pre-IC map. **e**, Angular distribution plot of the signal-subtracted, monomeric pre-IC map. **f**, Five views of the of the signal-subtracted, monomeric pre-IC map coloured according to the local resolution.

In previous cryo-EM work, we established that by the time the ATP–dCMGE complex is formed at an origin, all of the factors involved in its assembly process have been released^30^. We reasoned that, if ATP binding by MCM promotes this release, forming dCMGE in a buffer that lacks ATP might mean that the firing factors Sld2, Sld3/7 and Dpb11 are retained at origins, allowing us to reconstitute the pre-IC. Obtaining enough sample for cryo-EM analysis in such conditions is a challenge. Moreover, ATP is required for DH phosphorylation by DDK (ref. ^33^), as well as Sld2 and Sld3 phosphorylation by CDK (refs. ^10,11^). To circumvent this issue, we isolated the phospho-DH on origin DNA^30^, in a buffer lacking ATP (Supplementary Fig. 2a–e). Inspired by previous work^24^, we also phosphorylated recombinant Sld3/7 using CDK and repurified it in ATP-free conditions (prephosphorylated Sld3/7; hereafter, ppSld3/7; Supplementary Fig. 2f). We also cloned, expressed and purified a phosphomimetic variant of Sld2 containing 8 of 11 aspartate substitutions (hereafter, Sld2(8D)), previously shown to support Dpb11 binding and CDK bypass in cells^10,34^ (Supplementary Fig. 2g). We then tested ppSld3/7 and Sld2(8D) in a DNA replication reaction reconstituted in vitro with purified proteins^19^. DNA replication could be established in the absence of CDK with these reagents, although at reduced levels, compared with reactions containing CDK (Fig. 1b). Despite the reduction, ATP–dCMGE complexes^30^ could be assembled efficiently using ARS1-loaded phospho-DH, ppSld3/7 and Sld2(8D) in the absence of CDK, as observed by negative-stain electron microscopy (nsEM; Supplementary Fig. 3a,b). We then asked whether GINS and Cdc45 could be recruited to MCM using ppSld3/7 and Sld2(8D), in the absence of CDK and any nucleotide. nsEM two-dimensional (2D) averaging revealed a complex reminiscent of ATP–dCMGE^30^, but with MCMs engaged in tighter interaction and with no recognizable Pol ε density—at least at the limited resolution achieved with negative staining (Fig. 1c). Unlike ATP–dCMGE, the new ATP-free complex became disassembled when purified in high-salt conditions (Supplementary Fig. 3c–e). This observation agrees with previous western blot evidence that high-salt-resistant association of GINS and Cdc45 with licensed-origin DNA requires ATP binding^24^. To determine the composition of the ATP-free high-salt-sensitive assembly, we solved the cryo-EM structure (Extended Data Figs. 1 and 2). Inspection of the resulting density map, refined to 3.4-Å resolution after symmetry expansion^35^ (or 3.2 Å for the locally refined asymmetric unit), revealed a quasi-symmetric (flexible) assembly of two CMGs (Extended Data Table 1). Compared with the DH, Mcms 5, 3, 7 and 4 are disengaged across the two rings. Homodimerization through Mcm2 and Mcm6 persists as observed^20^ in the DH, with zinc finger domains acting as a hinge. Unoccupied density could be assigned to two copies of Sld3, Sld7, Dpb11 and the Dpb2 subunit of Pol ε, which, together with two CMG assemblies, form the pre-IC complex (Fig. 1d).

## Kinase-regulated Mcm4 engagement by Sld3

**Fig. 2:**
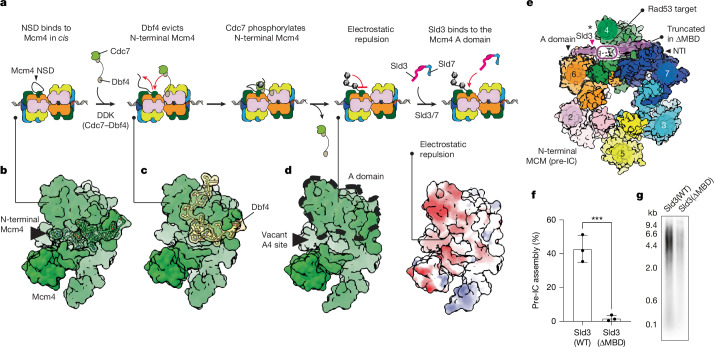
DDK enables the binding of Sld3 to Mcm4. **a**, Cartoon of the sequence of events leading to Sld3 recruitment. First, the DDK subunit Dbf4 dislodges N-terminal Mcm4 from the Mcm4 A domain. Then, DDK phosphorylation of N-terminal Mcm4 prevents its re-engagement with the A domain. Finally, Sld3 binds to the newly exposed Mcm4 A-domain epitope (vacant A4 site). **b**, Structure of non-phosphorylated Mcm4 (PDB: 7V3U). **c**, Structure of Mcm4 engaged by the Dbf4 subunit of DDK (PDB: 7PT7). **d**, Structure of phosphorylated Mcm4 (PDB: 7P30). Right, surface representation of phosphorylated Mcm4, coloured by electrostatic potential. **e**, Sld3 binds to the Mcm4 A domain as well as to the neighbouring NTI Mcm7 element and Mcm6 A domain. MBD, MCM-binding domain. The asterisk indicates Sld3 binding to the Mcm4 A domain. **f**,**g**, Truncation of the Sld3-MBD impairs pre-IC assembly (**f**; ***P = 0.0010 by two-tailed *t*-test; mean ± s.d.; experiment performed three times) and replication reconstituted in a test-tube (**g**; experiment performed twice). WT, wild type. For gel source data, see Supplementary Fig. 1.

Previous studies^16,17,36^ have established that docking of DDK onto the DH dislodges the N-terminal tail of Mcm4 from the A domain of Mcm4 (A4; Fig. 2a,b). A4 remains occupied by the Dbf4 subunit of DDK, while the Cdc7 kinase subunit phosphorylates the N-terminal Mcm4 tail that has become solvent-exposed (Fig. 2a,c). When DDK releases the DH, the negatively charged phospho-Mcm4 tail does not return to binding the electronegative A4 site, probably owing to electrostatic repulsion^16,17^ (Fig. 2a,d). Within the pre-IC, we find that the newly exposed A4 epitope becomes engaged by a C-terminal Sld3 α-helix, which in turn extends towards the two neighbouring subunits on either side of Mcm4. This same interaction was also predicted using AlphaFold 3^37^. N-terminally, Sld3 touches the Mcm6 A domain and C-terminally, it engages the Mcm7 NTI element (Fig. 2e). This Sld3 interaction might contribute to dislodging Mcm7 from its position across the two hexamers, where it protected the Mcm5 A domain in *trans*. We expressed and purified a truncated Sld3/7 complex lacking the Mcm4-binding site of Sld3 (∆515–538), to test whether this variant can still form the pre-IC and support DNA replication in reconstituted reactions. We found that, compared with the wild-type protein, both functions were almost completely blocked when using the truncated variant (Fig. 2f,g). Our results agree with previous biochemical data, which identified the same Sld3 region as being essential for MCM binding^23^. In addition to the interaction with Mcm4 described above, Sld3 has been observed to bind specific phosphorylated segments of Mcm4 and Mcm6 independently (not observed in our structure). This indicates that Sld3 has a distinct phospho-reader role, which complements its function in recognizing a DDK-dependent structural change in MCM (ref. ^23^).

Rad53 halts origin firing by targeting Sld3, as well as the Dbf4 subunit of DDK. In the Supplementary Results, we show that Rad53 phosphorylates a segment of Sld3 that is involved in binding to the A4 site, hence blocking origin firing by preventing Sld3 recruitment (Supplementary Figs. 4 and 5). This indicates that modulation of MCM engagement through phosphorylation by two different kinases has evolved to target three different interaction partners of the same Mcm4-binding pocket (Mcm4 itself, Dbf4^16,17,38^ and Sld3), which subsequently bind to MCM on the path to origin activation^9–13,33^.

## Sld3 tethers Cdc45 to MCM aided by Sld7

**Fig. 3:**
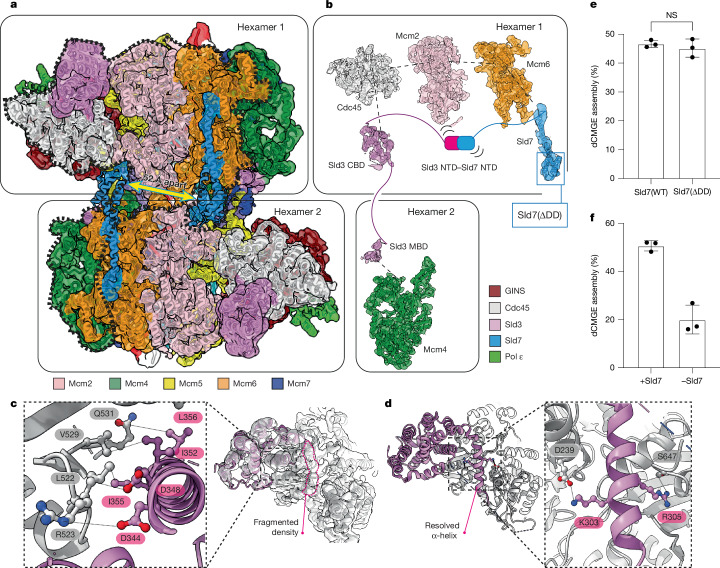
Sld3 and Sld7 recruit Cdc45 to MCM in the pre-IC. **a**, Sld3 engages Mcm4 on one MCM hexamer and deposits Cdc45 onto the opposing MCM hexamer (marked by the black dotted line). Two separate Sld7 molecules, 52 Å apart, straddle across two Mcm6 subunits. **b**, Interaction map of Sld3–Cdc45–Mcm2–Mcm6–Sld7 on one MCM hexamer and Sld3–Mcm4 on the opposing hexamer. See also Extended Data Fig. 3f,g. Although Sld3 is adjacent to Mcm4 on the opposing MCM ring, making C-terminal Sld3–Mcm4 engagement most likely, we cannot rule out the possibility that Sld3 takes a longer route, wrapping around Mcm2, Mcm6 and Sld7, to connect the Cdc45-binding domain (CBD) and MBD within the same MCM hexamer. See PDB 3X37. **c**, Cryo-EM density for Cdc45–Sld3. Detail of a first tethering point provided by an α-helix element (residues 340–359). Density for a second tethering point is fragmented (dotted pink line). **d**, Atomic model of the Cdc45–Sld3 determined by X-ray crystallography (PDB: 8J09). The inset shows Sld3 residues whose mutations impair Cdc45 recruitment and replication. **e**, Truncation of the Sld7 dimerization domain (Sld7(ΔDD)) does not impair dCMGE assembly (*P* = 0.4941 by two-tailed *t*-test; mean ± s.d.; experiment performed three times. NS, not significant). **f**, Omitting Sld7 allows dCMGE assembly, although at reduced efficiency (**P = 0.0011 by two-tailed *t*-test; mean ± s.d.; experiment performed three times).

Opposite the A4 element, on the juxtaposed MCM hexamer (in *trans*), Cdc45 is contacted by a central α-helical domain of Sld3 (amino acids 156–421), known as the Cdc45-binding domain^23^ (CBD) (Fig. 3a,b). A first tethering element of Sld3 is provided by an α-helix (residues 340–359), wedged in a hydrophobic groove in Cdc45. Mutational analysis previously showed that five of these residues (D344, D348, I352, I355 and L356) are essential for Cdc45 binding and cell viability (Fig. 3c). Cryo-EM density for the second tethering element was not as well resolved. To complement our atomic model, we docked the previously determined crystal structure of a yeast heterodimeric complex containing Cdc45 and the Sld3-CBD, which provided an excellent fit into the pre-IC map^39^. According to the crystal structure, an α-helix occupies the poorly defined density at the Sld3–Cdc45 interface, with Lys303 and Arg305 of Sld3 engaging in polar interactions with Glu239 and Ser647 of Cdc45, respectively (Fig. 3d). A 6× reverse-charge mutation variant of Sld3, including Lys303Glu and Arg305Glu, impairs Cdc45 engagement and replication in reactions reconstituted with purified proteins^23^. We conclude from these data that the incorporation of Cdc45 into the pre-IC involves the same interactions as those described for the isolated Sld3-CBD–Cdc45 heterodimer^39^.

**Extended Data Fig. 3:**
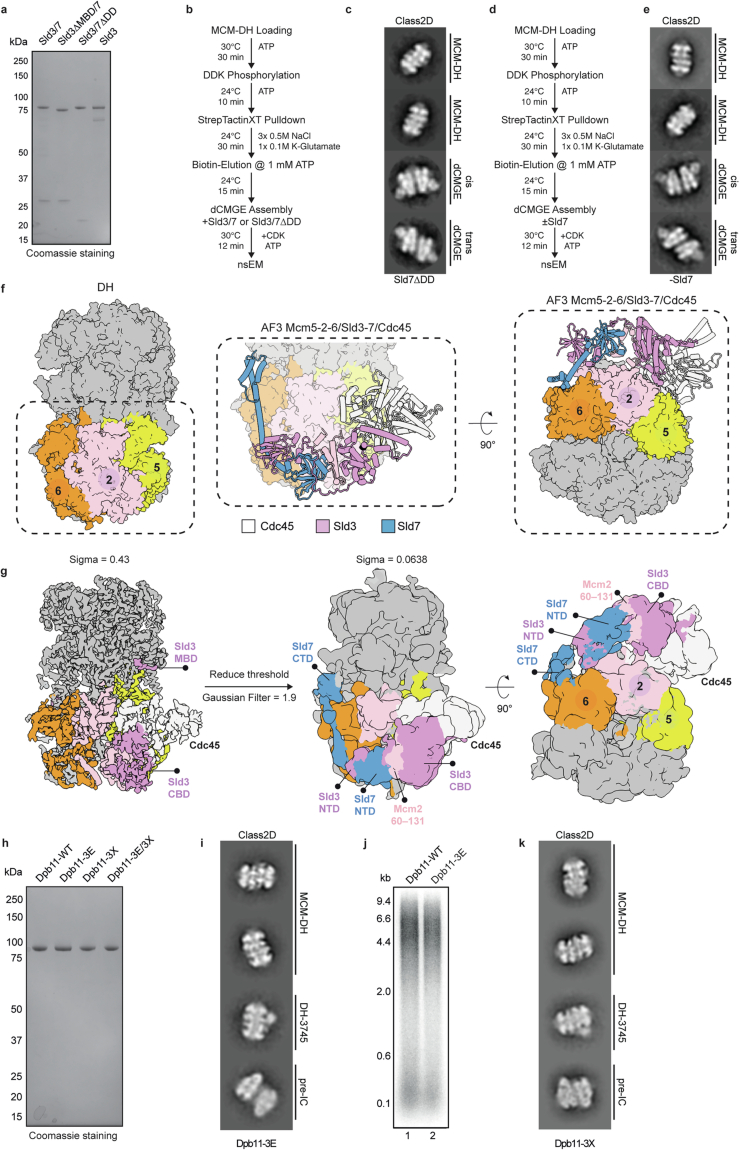
Sld7 requirement, Sld3/7–Cdc45 architecture on the DH and pre-IC assembly with Dpb11 variants. **a**, SDS–PAGE of purified Sld3 constructs. This gel was reproduced three times. **b**,**c**, Workflow (**b**) and representative 2D classes (**c**) for pre-IC assembly with wild type or Sld7 DD truncation mutant. **d**,**e**, Workflow (**d**) and representative 2D classes (**e**) for dCMGE formation with or without Sld7. **f**, AlphaFold 3 modelling of the entire Sld3–Sld7–Cdc45 interaction with Mcm6,2,5, overlaied with the experimentally determined DH structure. **g**, A cryoSPARC derived volume of the DH-3745 Gaussian-filtered in ChimeraX displaying the network of interactions between Sld3/7 and Cdc45 on the DH. **h**, Coomassie-stained gel for the wild type and mutant Dpb11 targeting the Mcm7 (3E), GINS (3X) or both (3E/3X) binding sites. This gel was repeated twice. **i**, 2D averages for the pre-IC formation reaction assembled with Dpb11(3E). **j**, DNA replication reactions with wild type and 3E variant of Dpb11. A small defect for the variant protein can be detected. The bigger effect observed in pre-IC assembly by EM (Fig. 4d) indicates that assessing the formation of this transient structural intermediate is a more sensitive assay compared to DNA replication reconstituted in vitro. This experiment was performed twice. **k**, 2D averages for the pre-IC formation reaction assembled with the Dpb11(3X) mutant. For gel source data, see Supplementary Fig. 1.

Sld3 was expressed and purified in complex with Sld7, a non-essential factor known to increase replication initiation efficiency^40,41^. We used AlphaFold 3 predictions^37^ to help locate cryo-EM density for Sld7 (amino acids 151–257). Sld7 features a 48-residue α-helix, straddling the outer perimeter of the Mcm6 N-terminal and ATPase domains, and ending with a C-terminal helical bundle pointing towards the N-terminal domain of MCM, where two hexamers meet. The local resolution of the pre-IC map was sufficient to allow model building of these Sld7 features (Fig. 3a,b). Crystallographic evidence was previously used to propose that Sld7 forms a functional homodimer in solution, mediated by its C-terminal dimerization domain (DD). This model supports a symmetrical mechanism for the concomitant recruitment of two Cdc45 factors. Although all pre-IC particles always contain two copies of Cdc45 (as well as GINS), we find that the two Sld7 DD domains do not engage in homodimerization; rather, they map 52 Å apart (Fig. 3a). To test the functional importance of the C-terminal module, we generated an Sld3/7 variant bearing an Sld7 truncation of residues 181–257 (Sld7(∆DD); Fig. 3b). We performed nsEM analysis to show that Sld7(∆DD) supports efficient dCMGE assembly (Fig. 3e and Extended Data Fig. 3a–c). Conversely, when Sld7 was omitted, dCMGE formation occurred inefficiently (Fig. 3f and Extended Data Fig. 3a,d,e). Although we were unable to visualize a direct interaction between Sld3 and Sld7 unambiguously in our pre-IC cryo-EM map, a co-crystal structure of the N-terminal heterodimerization domains exists^27^. AlphaFold 3^37^ co-folding of Mcm2, Mcm6, Cdc45, Sld3 and Sld7 predicts that this heterodimerization complex is tethered to the rest of the pre-IC through an unstructured element, connecting the Sld3-CBD to the Mcm6-interacting helix of Sld7 (Extended Data Fig. 3f). This would explain why the Sld3/7 heterodimerization element is not visible in our cryo-EM map.

## Dpb11 recruits GINS onto splayed DHs

**Fig. 4:**
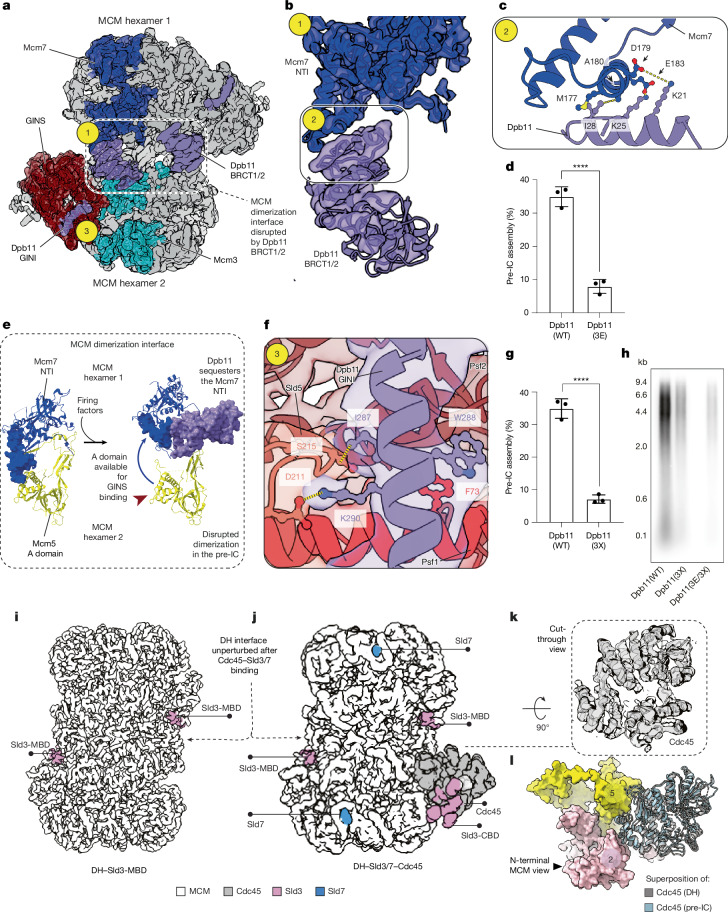
Structural role of Dpb11 in the pre-IC. **a**, Dpb11 wedges itself between Mcm3 and Mcm7, at the interface between two MCM hexamers (yellow label 1). Dpb11 also engages GINS (yellow label 3). BRCT1/2, BRCT1–BRCT2. **b**, Cryo-EM density with refined atomic model of Mcm7–Dpb11. Yellow label 2 indicates the Dpb11–Mcm7 interaction interface. **c**, AlphaFold 3 prediction of the Mcm7–Dpb11 interaction interface. **d**, Mutating three Mcm7-interacting residues in Dpb11 (Dpb11(3E)) causes a big drop in pre-IC complex formation, as observed by nsEM (****P < 0.0001 by one-way ANOVA; mean ± s.d.; experiment performed three times). **e**, Mcm5–Mcm7 compared in the DH and pre-IC structures. In the DH, the Mcm7 NTI protects the Mcm5 A domain. In the pre-IC, the Mcm7 NTI is sequestered by Dpb11, rendering the Mcm5 A domain available for GINS binding. **f**, Detailed view of the interaction between GINS and the GINI element of Dpb11. **g**,**h**, A three-amino-acid change in GINI impairs pre-IC assembly (**g**) and replication (**h**). In **g**, ****P < 0.0001 by one-way ANOVA; mean ± s.d.; experiment performed three times. Panels **d** and **g** show the same wild-type condition because Dpb11 variants were probed in the same three replicate experiments. A mutant that also targets the Dpb11–Mcm7 interaction exacerbates the replication defect (this experiment was performed twice). For gel source data, see Supplementary Fig. 1. **i**, Surface rendering of a DNA-loaded MCM-DH bound by the Sld3-MBD. **j**, Surface rendering of the DH structure bound by Sld3/7–Cdc45. Two copies of Sld3/7 and one copy of Cdc45 are visible. The Sld3-CBD recruits Cdc45 to MCM. **k**, Detail of the cryo-EM density for Cdc45. **l**, Cdc45 is slotted between the A domains of Mcm2 and Mcm5, in a position virtually identical to that observed in the pre-IC structure.

Wedged in between the two splayed MCM rings, we identified two copies of Dpb11 (BRCA1 C terminus, BRCT1 and BRCT2 domains). Although they are proximal, these two Dpb11 subunits do not interact. Rather, they symmetrically bridge between two Mcm3 and Mcm7 subunits aligned across the two MCM rings of the pre-IC (Fig. 4a). Despite the limited resolution of the interaction interface, we could recognize α-helix 1 of BRCT1 directly engaging the Mcm7 NTI (Fig. 4b). AlphaFold 3^37^ recapitulated the same interaction interface, allowing us to identify three Dpb11 candidate residues engaged in a network of polar and hydrophobic contacts with Mcm7 (Fig. 4c). To disrupt these contacts, we introduced three mutations in Dpb11 (K21E, K25E and I28E; hereafter, Dpb11(3E)) (Extended Data Fig. 3h). When using this Dpb11 variant, we observed a fourfold decrease in the DH-to-pre-IC conversion efficiency, and a small drop in the replication signal from reconstituted reactions (Fig. 4d and Extended Data Fig. 3h–j). Dpb11 sequesters the Mcm7 NTI, which no longer protects the Mcm5 A domain of the opposed MCM hexamer in the DH (ref. ^20^). This structural change exposes an epitope on the Mcm5 A domain, allowing the engagement of GINS through the Psf2 subunit^22^ (Fig. 4e). Dpb11 also directly engages GINS subunits Psf1, Psf2 and Sld5, through an α-helical element (residues 273–295; Fig. 4f). Despite the poor sequence conservation, the same GINS-interaction (GINI) element was previously identified in a comparative biochemical study of yeast Dpb11 and the orthologous TopBP1 protein of *Xenopus laevis*^42^. Indeed, a cryo-EM structure of a frog TopBP1–GINS complex revealed a structurally conserved GINI α-helix serving the same function as that observed in our yeast pre-IC^43^. We introduced three mutations in Dpb11 GINI residues (I287S, W288A and K290E; Dpb11(3X)) involved in GINS engagement (Extended Data Fig. 3h). Using this mutant protein in reconstitution experiments yielded a fourfold drop in DH to pre-IC formation efficiency, and a severe replication defect (Fig. 4g,h and Extended Data Fig. 3k). When combining the Mcm7 NTI interaction and GINI mutants (Dpb11(3E/3X)) we observed an additive detrimental effect, with a replication product barely above background level (Fig. 4h). Our structure uncovers an unexpected function of Dpb11, in which it acts across the pre-IC, through engagement of Mcm7 on one MCM hexamer and recruitment of GINS onto the opposed hexamer. Thus, just like DDK (ref. ^14^) and Sld3 (described above), each copy of Dpb11 engages two opposed MCM hexamers that contain the symmetry to support bidirectional replication. Furthermore, in the Supplementary Results, we explain how Dpb11 exerts its reported phospho-reader function. The BRCT1–BRCT2 epitope on Dpb11 is solvent-exposed and poised to bind to CDK-phosphorylated Sld3 (Supplementary Fig. 6a–d), providing another example of an essential, kinase-driven mechanism that triggers origin firing^10,11^.

## What disrupts the DH interface

**Extended Data Fig. 4:**
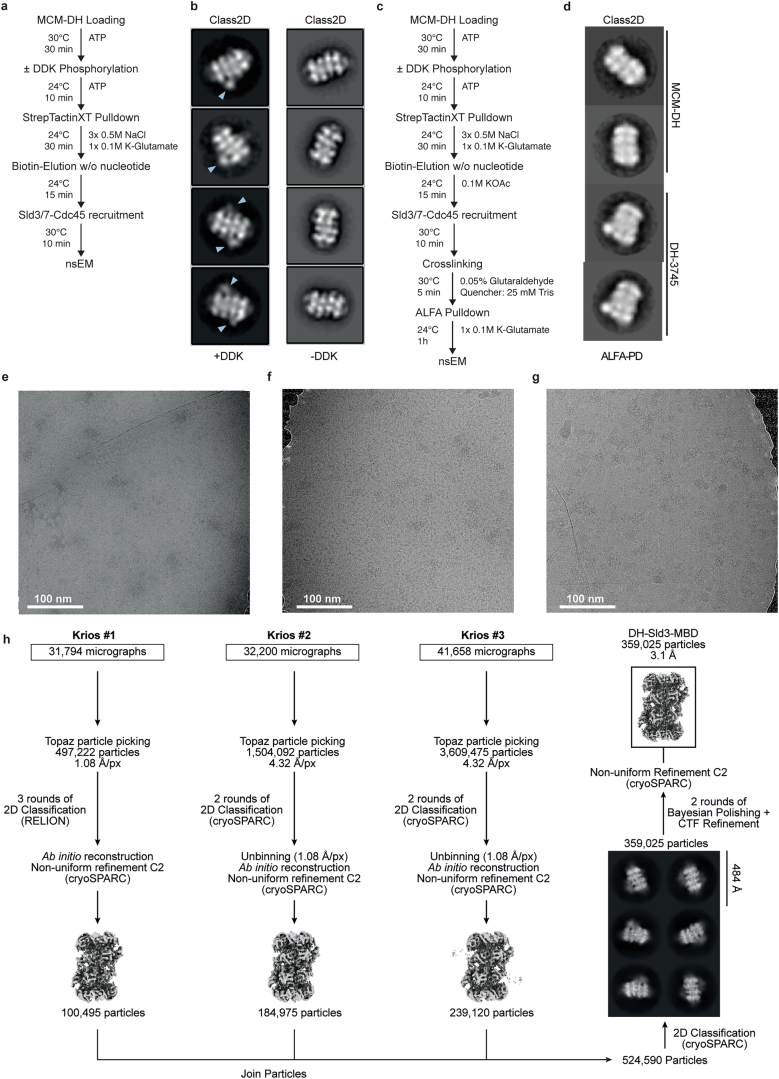
DH–Sld3/7–Cdc45 assembly and processing pipeline for the DH–Sld3-MBD. **a**, Workflow for the preparation of the non-cross-linked DH–Sld3/7–Cdc45. **b**, 2D averages obtained by supplementing (left) or omitting (right) DDK. The blue arrows indicate Cdc45 density. Our first nsEM attempts resulted in decoration of DHs with poorly averaged density on the side of either one or both MCM hexamers. **c**, Workflow for the preparation of DH–Sld3/7–Cdc45 (cross-linked and purified). **d**, 2D averages obtained for the cross-linked and purified DH–Sld3/7–Cdc45. Improved DH decoration was obtained after glutaraldehyde cross-linking followed by nanobody-affinity purification using an ALFA tag^97^, a second, cross-linking-tolerant handle fused to the MH DNA roadblock. **e**–**g**, Representative cryo-electron micrographs from each individual dataset. The respective samples were reconstituted more than three times and imaged under cryo conditions two (**e**) and four (**f**,**g**) times. **h**, Pipeline for determining the DH–Sld3-MBD structure.

**Extended Data Fig. 5:**
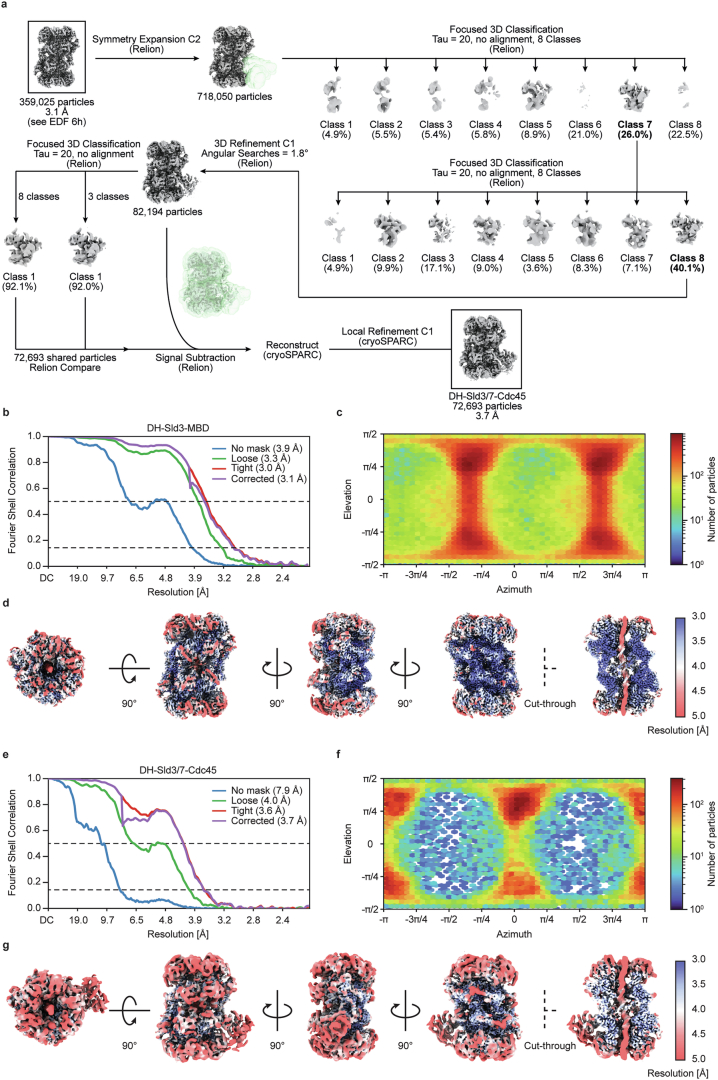
Processing pipeline for DH–Sld3/7–Cdc45 and validation of the DH–Sld3-MBD and DH–Sld3/7–Cdc45 maps. **a**, Pipeline for determining the DH–Sld3/7–Cdc45 structure. **b**, Fourier shell correlation of the DH–Sld3-MBD map. **c**, Angular distribution plot of the full dimeric DH–Sld3-MBD map. **d**, Five views of the of the DH–Sld3-MBD map coloured according to the local resolution. **e**, Fourier shell correlation of the DH–Sld3/7–Cdc45 map. **f**, Angular distribution plot of the DH–Sld3/7–Cdc45 map. **g**, Five views of the of the DH–Sld3/7–Cdc45 map coloured according to the local resolution.

From the pre-IC structure alone, it is unclear whether Dpb11 binding breaks the DH interface or Dpb11 accesses a preformed gap, and whether Mcm7–Sld3 engagement causes or is enabled by DH cracking. Previous reports indicate that pre-IC formation involves two biochemical steps that can be staged^9,23,24,40^. To reconstitute the Cdc45 assembly step, we repeated the pre-IC formation reaction omitting Sld2, Dpb11, Pol ε and GINS^30^ (Extended Data Fig. 4a–d), and analysed the reaction by cryo-EM. We solved a 3.1-Å-resolution structure of a DH featuring Sld3 density at the A4 site, which might represent a first recruitment intermediate. In this structure, the Mcm7 NTI still interacts tightly with Mcm5, sealing the interface between the two hexamers (Fig. 4i). After symmetry expansion, three-dimensional (3D) classification and local refinement, we also solved a 3.7-Å-resolution structure of DH–Sld3/7–Cdc45 (DH-3745; Fig. 4j). The Sld3-CBD became visible, if at lower local resolution, reflecting its high flexibility here compared with in the pre-IC structure (Extended Data Figs. 4e–h and 5 and Extended Data Table 1). Cdc45 slots in between the A domains of Mcm2 and Mcm5, occupying the same position observed in the pre-IC and CMG (Fig. 4k,l). Sld7 was also observed to engage Mcm6, at the same ATPase site as was observed in the pre-IC (Fig. 4j and Extended Data Fig. 3g). Parallel 3D classification efforts provided some evidence for the flexible N-terminal domains connecting Sld3 and Sld7, compatible with previous X-ray work^27^ and the AlphaFold 3^37^ prediction described above (Extended Data Fig. 3f,g). MCM in the DH-3745 structure is similar to a DDK-treated DH solved in ATP-containing buffer^14^, although the nucleotide occupancy is different^38^ (Supplementary Fig. 7a,b). The MCM dimerization interface of the DH does not change after Sld3/7–Cdc45 binding, and an overlay of DH with Mcm7–Dpb11 extracted from the pre-IC structure reveals extensive steric clashes with Mcm5 (Supplementary Fig. 7c). We conclude that the separation between the two MCM rings must involve the recruitment of Sld2, Dpb11, GINS and Pol ε, or a subset of these factors.

Our EM imaging of reconstituted Cdc45 recruitment by Sld3/7 reveals one or two Cdc45 molecules engaged to DHs at any one time (Extended Data Fig. 4b,d), suggesting that Cdc45 is recruited to each MCM hexamer in independent events. This is compatible with single-molecule measurements using yeast proteins^25^, and different from single-molecule observations with frog egg extracts. In fact, in the *Xenopus* system, Cdc45 is recruited to DHs synchronously, through a mechanism that has yet to be elucidated^44^.

In the Supplementary Results, we discuss the changes in DNA engagement that are triggered by the transition between DH-3745 and the pre-IC (Supplementary Fig. 8a,b).

## How CMG assembly factors are ejected

**Extended Data Fig. 6:**
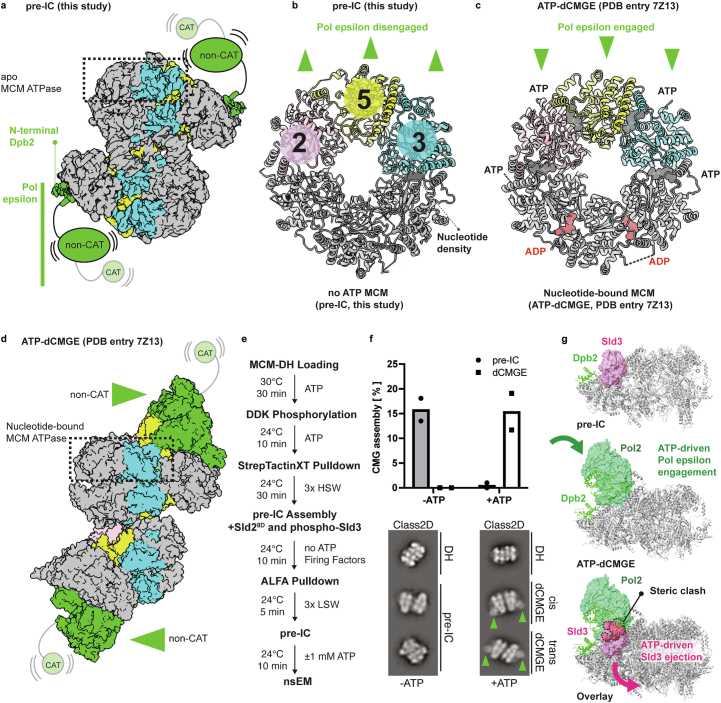
Pol ε in the pre-IC. **a**, The only Pol ε element visible in the pre-IC is N-terminal Dpb2. **b**, In the pre-IC, no nucleotide density can be observed within the Mcm2–5 interface. In the same structure, Pol ε is disengaged from the ATPase tier of MCM. **c**, In the ATP–dCMGE structure, the Mcm2–5 interface is ATP-bound and Pol ε engages the ATPase domain. **d**, Side view of ATP–dCMGE, with the non-catalytic Dpb2–C-terminal-Pol2 portion of Pol ε visible. **e**, Flow chart of pre-IC formation in apo conditions, followed by addition of ATP. **f**, In apo conditions, only pre-ICs and no dCMGE can be observed. When ATP is spiked in, most pre-ICs are converted to dCMGE, where Pol ε decorates CMG on the ATPase face of MCM. This experiment was performed twice. **g**, Mechanism for Sld3 eviction, through the ATP-dependent engagement of C-terminal Pol2 to the ATPase side of MCM.

The only Pol ε element visible in the pre-IC is the N-terminal domain of Dpb2 (Extended Data Fig. 6a). This observation caught our attention for two reasons. First, the isolated N-terminal Dpb2 was observed to support CMG assembly after depletion of endogenous Dpb2 in yeast cells^18^. Second, according to structural studies on CMGE bound to an artificial DNA fork, N-terminal Dpb2 is the only Pol ε element detected in CMG when the Mcm2–5 site is nucleotide-free^45^ (just as in the pre-IC; Supplementary Fig. 7b and Extended Data Fig. 6b). Instead, when Mcm2–5 is ATP-bound, the non-catalytic portion of Pol ε can be averaged in full, stably anchored to MCM^30,45,46^ (as seen in ATP–dCMGE; Extended Data Fig. 6c,d). Inspired by these observations, we hypothesized that not only Dpb2 but the full Pol ε complex might be tethered to CMG in our pre-IC structure, and that it would become fully MCM engaged (visible) when ATP was added. To test this hypothesis, we assembled the pre-IC in the absence of nucleotide, as described above, and purified this complex having washed away excess Pol ε (Extended Data Fig. 6e). After elution and incubation with or without ATP we imaged particles by nsEM. Only when ATP was supplemented did we recognize the non-catalytic Pol ε decoration that is characteristic of ATP–dCMGE complexes. As observed before, the two CMGE particles were present in both the *cis* and the *trans* configuration^30^ (Extended Data Fig. 6f).

The ATP-dependent transition from the pre-IC to dCMGE suggests a mechanism for how ATP binding at an origin of replication might stabilize the topological binding of CMG to DNA, making it resistant to high-salt washes^24^. In fact, whereas GINS and Cdc45 latch across the Mcm2–5 gate on the N-terminal MCM side of CMG, fully engaged Pol ε stabilizes the C-terminal MCM side, by securing GINS and Cdc45 to the Mcm2-5-3 ATPase domains^46^. An ancillary role of Pol ε in CMG assembly is discussed in Supplementary Fig. 7d,e.

Tight Pol ε engagement to C-terminal CMG in the ATP–dCMGE complex also addresses a key unknown in replication origin activation: how the eviction of assembly factors is triggered through ATP binding by MCM^24,30^. In fact, Cdc45 engagement by C-terminal Pol2 sterically clashes with the Sld3-CBD, providing a mechanism for Sld3 ejection (Extended Data Fig. 6g). The ability of two ATP–CMGEs to rotate with respect to one another^30^ provides another means for the release of CMG assembly factors. Indeed, as soon as two CMGs visit a *cis* dCMGE configuration, Dpb11 will be dislodged from its position, nestled between Mcm3 and Mcm7 across the two hexamers (Fig. 4a). CMG rotation would also lead to the eviction of Sld3 itself, because Sld3 binds to Cdc45 on one CMG and Mcm4 in the opposed CMG across the pre-IC (Fig. 3b).

## Sld2 is essential after CMGE assembly

**Fig. 5:**
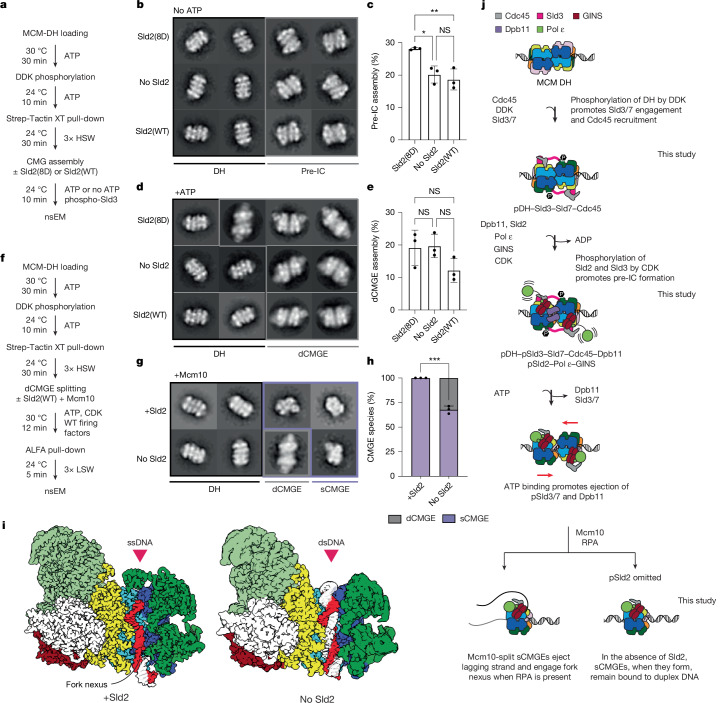
Role of Sld2 in CMG assembly. **a**, Flow chart of pre-IC and dCMGE formation with or without Sld2 (8D variant or wild type). **b**, Representative 2D averages of the pre-IC assembly reaction. **c**, Bar graphs of pre-IC formation with or without Sld2 (8D variant or wild type). *P* values by one-way ANOVA: 8D versus no Sld2, *P = 0.0160; 8D versus Sld2(WT), **P = 0.0070; no Sld2 versus Sld2(WT), *P* = 0.7293; mean ± s.d.; experiment performed three times. **d**, Representative 2D averages of the dCMGE assembly reaction. **e**, Bar graphs of dCMGE formation with or without Sld2 (8D variant or wild type). *P* values by one-way ANOVA: 8D versus no Sld2, *P* = 0.9875; 8D versus Sld2(WT), *P* = 0.1981; no Sld2 versus Sld2(WT), *P* = 0.1635; mean ± s.d.; experiment performed three times. **f**, Flow chart of dCMGE splitting by Mcm10 in the presence or absence of Sld2. LSW, low-salt wash. **g**, Representative 2D averages of the dCMGE splitting reaction. **h**, Bar graph showing that Sld2 is not strictly required to split dCMGE into sCMGEs, but that its absence negatively affects splitting efficiency. ***P = 0.0001 by two-tailed *t*-test; error bars, mean + s.d.; experiment performed three times. **i**, Cut-through side view of sCMGE assembled in the presence of Mcm10 and RPA, with or without Sld2. In the presence of Sld2, sCMGE engages the fork nexus at the N-terminal front. Only the leading-strand template enters the MCM pore. In the absence of Sld2, duplex DNA is observed running through the entire length of the MCM pore. The structure assembled with Sld2 is coloured according to a newly built atomic model. The structure assembled without Sld2 is coloured according to a previously published atomic model of double-stranded DNA–CMGE (PDB: 7QHS). **j**, Cartoon representation of origin activation, showing that Sld2 has an essential origin-activation function downstream of CMG formation.

In the pre-IC cryo-EM map, density for all factors previously implicated in CMG assembly can be identified, except for one, Sld2. This was not surprising, given that phospho-Sld2 is known^34^ to engage a flexibly tethered region of Dpb11, which is not resolved in our pre-IC structure. To establish whether Sld2 is indeed required for GINS recruitment, as commonly accepted, we assembled the pre-IC as described above, and analysed the reaction by nsEM, either using the full complement of firing factors or omitting individual components. Whereas ppSld3/7, GINS and Cdc45 were all required for pre-IC formation (Supplementary Fig. 7d,e), leaving out the Sld2(8D) phosphomimetic variant still yielded recognizable pre-IC averages, although the DH-to-pre-IC conversion rate dropped by roughly one-third (from 28% to 20%, on the basis of 16,318 averageable particles for Sld2(8D) and 22,234 for the dropout, each over three biological replicates; Fig. 5a–c). Similarly reduced efficiency (from 20,030 averageable particles) was obtained when swapping Sld2(8D) for non-phosphorylated, wild-type Sld2 (Fig. 5a–c). When these experiments were repeated in the presence of ATP, dCMGE^30^ could be assembled^30^ with robust efficiency, reflecting the increased stability provided by nucleotide binding^24^ (analysis based on 55,833 averageable particles over the three conditions; Fig. 5d,e).

So far, we have shown that Sld2 can stimulate but is not needed for pre-IC assembly or dCMGE formation. Previous biochemical reconstitution experiments, however, established that phospho-Sld2 is strictly required for origin-dependent DNA replication^40^. Given that we can reproduce the observation with the same purified proteins used in our nsEM experiments (not shown), we conclude that an essential function of Sld2 during origin activation must be performed downstream of dCMGE assembly. To work out which step after CMGE formation requires phospho-Sld2, we reconstituted Mcm10-dependent dCMGE splitting from purified DDK-phosphorylated DHs, in the presence of CDK and replication protein A (RPA), with or without wild-type Sld2. We further purified this preparation using paramagnetic beads to pull on an ALFA-tagged covalently linked methyltransferase roadblock capping the ends of the double helix, to ensure that all helicase particles imaged were engaged with DNA (Fig. 5f). According to our nsEM analysis, when Sld2 was included, only DHs and single CMGEs (sCMGEs) were observed (on the basis of 28,577 averageable particles). This recapitulates previous observations that all dCMGEs assembled are split into sCMGE when Mcm10 is added^47^. When Sld2 was omitted, we instead observed a mixture of dCMGEs (32%) and sCMGEs (68%, from 25,386 averageable particles over three repeats; Fig. 5g,h). We then asked whether sCMGE that is assembled on origin DNA without Sld2 is different from sCMGE obtained with the complete set of firing factors. To address this, we analysed the two reactions by cryo-EM.

**Extended Data Fig. 7:**
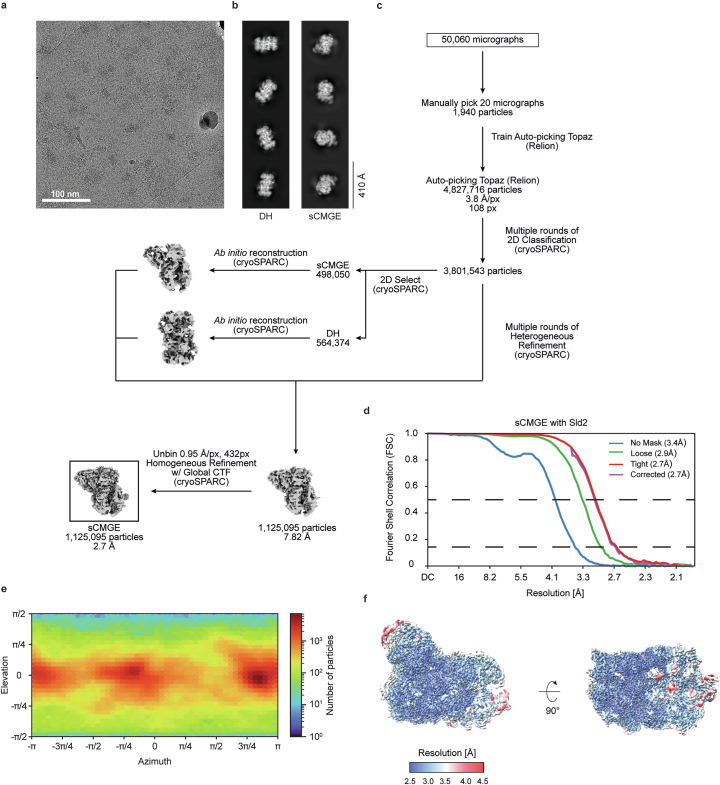
Processing pipeline and validation of the sCMGE assembled at the ARS1 origin with Mcm10, RPA and Sld2. **a**, Representative cryo-electron micrograph. This sCMGE formation experiment was repeated three times. **b**, 2D averages of inactive DHs and activated sCMGEs. **c**, Image-processing pipeline. **d**, Fourier shell correlation plot. **e**, Angular distribution. **f**, sCMGE filtered and coloured according to the local resolution.

**Extended Data Fig. 8:**
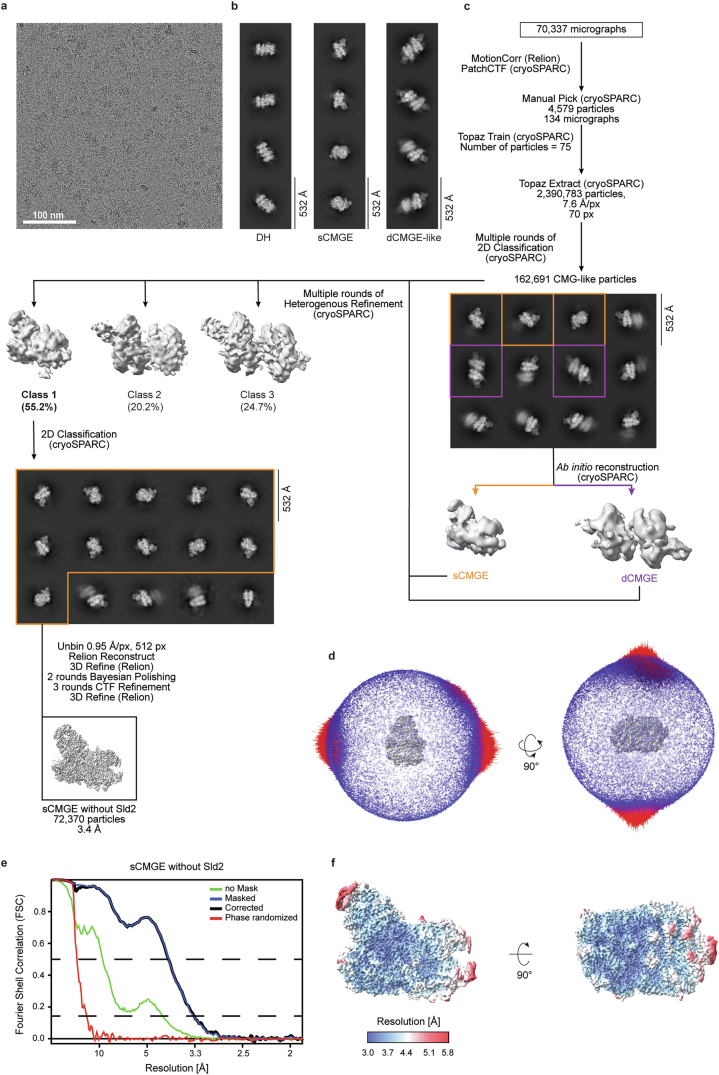
Processing pipeline and validation of the sCMGE assembled at the ARS1 origin with Mcm10 and RPA and without Sld2. **a**, Representative cryo-electron micrograph. This sCMGE formation experiment was repeated three times. **b**, 2D averages of inactive DHs, sCMGEs and split sCMGEs. **c**, Image-processing pipeline. **d**, Angular distribution. **e**, Fourier shell correlation plot. **f**, sCMGE filtered and coloured according to the local resolution.

When Sld2 and RPA were present, the 3D structure revealed sCMGE engaged with the fork nexus, with single-stranded DNA running through the MCM central channel (no sCMGE engaged with duplex DNA could be identified in this condition, when further 3D classification was attempted). When Sld2 was left out, however, sCMGE was found to be bound to duplex DNA (no sCMGE bound to single-stranded DNA could be identified). The engagement of the double helix in this context seems to be identical to what was observed^30^ in dCMGE (Protein Data Bank (PDB) entry 7QHS; Fig. 5i, Extended Data Figs. 7 and 8 and Extended Data Table 2), or in sCMGE at termination^48^. Our data imply that Sld2 has an essential role in lagging-strand ejection after dCMGE formation.

## Discussion

The factors involved in eukaryotic DNA replication initiation are mostly conserved from yeast to humans^49^. However, there are exceptions, in terms of both the function and the identity of the proteins involved. For example, all six subunits of the origin recognition complex (ORC), the loader of the MCM helicase, are essential for helicase loading in yeast, but not in humans^50,51^. Likewise, Mcm10 is required to split the dCMGE and establish bidirectional replication in yeast, but it is dispensable in *Caenorhabditis elegans*^52^. The picture becomes more complicated when one specific gene is lost during evolution, because other factors must be repurposed to fulfil the missing function. This is the case for DONSON, which is present in most eukaryotes but has been lost in yeast. Studies in metazoans have established^52–55^ that DONSON performs an essential function during CMG assembly, by delivering GINS to MCM. In yeast, the GINS delivery function has previously been assigned to Sld2, on the basis of observations that GINS is not detected on chromatin when Sld2 is absent^10,26^.

Whether DONSON does indeed perform the same function as yeast Sld2, as recently proposed, is not clear. Phylogenetic analysis quickly unveils a complex scenario, casting doubt on the matter. For example, *C. elegans* encodes both DONSON and yeast-like SLD2 (ref. ^56^), whereas most of the other DONSON-containing eukaryotes encode the RECQL4 helicase^57^, a multidomain protein with an Sld2-homology domain. This domain alone is essential for origin activation in *X. laevis*, but functions downstream of—not during—CMG assembly^58–61^. Thus, a more appropriate question is whether the role of Sld2 (metazoan RECQL4) has been repurposed in yeast to make up for the loss of DONSON.

Our results do not fully support this scenario. In agreement with previous work, we find that Sld2 is involved in the efficient delivery of GINS to MCM, as observed in the pre-IC assembly step of CMGE biogenesis. However, Sld2 is not absolutely required for making a stable dCMGE complex. Instead, it has an essential role after holohelicase assembly. In fact, we find that dCMGE splitting is defective without Sld2, and helicases that still mature to the sCMGE state, in these conditions, do not transition to engaging single-stranded DNA; instead, they remain bound to duplex DNA. We conclude that the processes of double CMGE splitting, MCM gate opening and controlled lagging-strand ejection must be tightly coupled, and that all require Sld2 to function productively (Fig. 5j). In this respect, we note that according to western blot analysis of the same reconstituted system, the amount of CMG retained on DNA in the presence of Mcm10 is markedly reduced when Sld2 is omitted^40^. Does this imply that selective-strand ejection fails without Sld2, such that not only the lagging- but also the leading-strand DNA can be evicted, putting CMGEs at risk of falling off the double helix? Is the loss of a sCMGE partner the reason that orphan sCMGEs remain bound to duplex and do not transition to single-stranded DNA? In other words, can lagging-strand ejection occur only when two CMGEs cross paths? From an evolutionary perspective, is the lagging-strand ejection function of Sld2 conserved in RECQL4 (refs. ^58–61^)? Further work is needed to address these topics.

Another question has yet to be answered: which yeast factor, if not Sld2, performs the role of DONSON during CMG formation? When inspecting the yeast pre-IC and the *X. laevis* double CMG–DONSON complex^62^, we noticed a tantalizing similarity. Just like frog DONSON, yeast Dpb11 sits across the N-terminal homodimerization domain of MCM, and concomitantly engages GINS and Mcm3. On the basis of this observation, we suggest that Dpb11 takes on both the essential phospho-reader role of TopBP1 (its known orthologue)^63^ and the GINS-recruitment function of DONSON.

*Note added in proof*: While our manuscript was under review, two related preprints were posted^64,65^. Sonneville et al.^64^ reported that *C. elegans* SLD2 acts with MCM10 to activate CMG; and Bektash et al.^65^ reported similar findings for human RECQL4. These two studies agree with our observation that yeast Sld2 functions during lagging-strand ejection.

## Methods

### Protein expression and purification

HpaII methyltransferase (MH), ORC, Cdc6, Mcm2–7–Cdt1, DDK, CDK, (yeast-expressed) Sld3/7, Cdc45, GINS, (yeast-expressed) Pol ε, Mcm10, RPA, topoisomerase I (TopoI), Pol α and Rad53 were expressed and purified as previously described^24,30,40,46,66–69^. All mutant constructs were expressed and purified following the same protocol as was used for the wild-type protein unless stated otherwise. All buffers described below are also reported in Supplementary Table 1.

### Cell lines

Sf21 insect cells were obtained in-house from the Cell Services Science and Technology Platform. These cells were not authenticated and tested negative for mycoplasma contamination.

### Cloning, expression and purification of Twin-Strep-tagged Sld3/7

Codon-optimized gene blocks (IDT) encoding *Saccharomyces cerevisiae* Sld3 in-frame with a tobacco etch virus (TEV) protease cleavage site and a C-terminal Twin-Strep-tag (TST) as well as *S. cerevisiae* Sld7 were inserted into GoldenBac shuttle vectors pGB-01;02 and pGB-02;03. Subsequently, Sld3-TEV-TST and Sld7 expression cassettes were subcloned into pGB-dest using GoldenBac assembly^70^ and transformed into electrocompetent EMBacY cells (Geneva Biotech). Cells were screened by blue–white selection for successful bacmid integration and selected colonies were grown overnight at 37 °C. Cells were collected by centrifugation and bacmids were purified by isopropanol precipitation. A total of 45 µg bacmid DNA was mixed thoroughly with 13.5 µl FuGENE HD Transfection Reagent (Promega) and 450 µl Sf-900 III SFM medium and incubated for 30 min at room temperature. Two hundred microlitres of transfection mix was added dropwise to 2 ml Sf21 insect cells seeded at 0.5 million cells per ml in a six-well plate. Plates were incubated for 3–5 days at 27 °C in a wet-towel box. Efficient transfection was monitored through YFP fluorescence. Adherent cells were resuspended and supernatant containing P0 baculovirus was collected. To increase the multiplicity of infection, 46 ml Sf21 insect cells at 0.5 million cells per ml were inoculated with 4 ml P0 baculovirus in suspension and cultured at 27 °C shaking at 120 rpm. After viability had dropped below 90%, cells were pelleted by centrifugation at 380*g* for 15 min at 4 °C, and supernatant containing the amplified P1 virus was sterile-filtered (0.22 µm pore size) and stored at 4 °C. One litre of Sf21 insect cells were seeded at one million cells per ml in Sf-900 III SFM medium and infected with 0.5 % v/v P1 virus. Cells were collected 48 h after baculovirus-induced cell-cycle arrest by centrifugation for 15 min at 180*g* at 4° C, and pellets were flash-frozen in liquid nitrogen and stored at −80 °C.

Cells were thawed and resuspended in 50 ml buffer A (25 mM HEPES-KOH pH 7.5, 500 mM KCl, 10% v/v glycerol, 0.02% w/v NP-40, 1 mM EDTA and 1 mM DTT) supplemented with one cOmplete EDTA-free protease inhibitor tablet (Merck) and 0.7 mM phenylmethylsulfonyl fluoride (PMSF), then lysed by sonication on ice for 2 min (1-s pulse-on, 4-s pulse-off). The lysate was clarified by ultracentrifugation for 1 h at 45,000 rpm in a Ti45 rotor (Beckman) at 4 °C, and the supernatant was mixed with 2.4 ml Bio-Lock (IBA) reagent and applied onto 1 ml pre-equilibrated Strep-Tactin XT Superflow HighCapacity resin in a gravity column. The resin was washed with 100 ml buffer A and 10 mL buffer A supplemented with 2 mM ATP and 10 mM MgCl_2_. Protein was eluted with 10 ml buffer A supplemented with buffer BXT (IBA). The eluate was pooled, concentrated and loaded onto a Superdex 200 Increase 10/300 GL column (Cytiva) equilibrated in buffer A. Gel-filtered Sld3/7 was concentrated to approximately 1.5 mg ml^−1^, aliquoted and flash-frozen in liquid nitrogen.

### Cloning, expression and purification of Dpb11

Codon-optimized *S. cerevisiae* Dpb11 followed by a 3C protease cleavage site and a C-terminal 3×Flag tag was subcloned into a pGB-04;05 shuttle vector and subsequently transformed into electrocompetent EMBacY cells. Bacmid and baculoviruses were prepared as described above; 1 l Sf21 insect cells at one million cells per ml were infected with 0.5% v/v P1 virus and collected 48 h after cell-cycle arrest.

The cell pellet was resuspended in 50 mL buffer A supplemented with one cOmplete EDTA-free protease inhibitor tablet (Merck) and 0.7 mM PMSF, lysed by sonication on ice for 2 min (1-s pulse-on, 4-s pulse-off) and ultracentrifuged at 45,000 rpm at 4 °C for 1 h. The soluble phase was mixed with 2.4 ml Bio-Lock Reagent and passed through 1 ml pre-equilibrated anti-Flag M2 Affinity Gel (Sigma) in a gravity column. The column was washed with 150 ml buffer A and 10 ml buffer A supplemented with 2 mM ATP and 10 mM MgCl_2_. To elute bead-bound protein, the beads were resuspended in 5 ml buffer A supplemented with 0.5 mg ml^−1^ 3×Flag peptide and incubated for 5 min, after which the flow-through was collected. The eluate was diluted to 150 mM KCl and loaded onto a 1 ml Mono S 5/50 column (Cytiva) equilibrated in buffer B (25 mM HEPES-KOH pH 7.5, 150 mM KCl, 10% v/v glycerol, 0.02% w/v NP-40, 1 mM EDTA and 1 mM DTT). After washing the column with 10 ml buffer B, Dpb11 was eluted with a linear gradient of 150–1,000 mM KCl in buffer B over 20 column volumes. Fractions containing pure Dpb11 were pooled and dialysed against buffer C (25 mM HEPES-KOH pH 7.5, 300 mM KOAc, 10% v/v glycerol, 0.02% NP-40, 1 mM EDTA and 1 mM DTT) at 4 °C overnight while stirring. Subsequently, Dpb11 was concentrated to approximately 0.5 mg ml^−2^, aliquoted and flash-frozen in liquid nitrogen.

Dpb11 mutants containing charge-reversal substitutions (Dpb11(3E), Dpb11(3X) and Dpb11(3E/3X)) were purified using gel filtration instead of cation-exchange chromatography. The reason for this alteration was the predicted isoelectric point of the mutant proteins, which matches the pH of buffers B and C. After Flag affinity purification, the eluate was concentrated and loaded onto a Superdex 200 Increase 10/300 GL column equilibrated in buffer C. Dpb11-containing fractions were pooled, concentrated to approximately 0.5 mg ml^−1^, aliquoted and flash-frozen in liquid nitrogen.

### Cloning, expression and purification of Sld2

An expression cassette encoding *S. cerevisiae* Sld2 in-frame with an N-terminal VNp6 peptide tag^71^ and a retro-protein XXA solubility tag^72^, as well as a C-terminal Twin-Strep-tag, was subcloned into a pET303 backbone and transformed into bacterial T7 Express cells. Multiple colonies were picked to inoculate 4× 1 l LB + 100 µg ml^−1^ carbenicillin and incubated static overnight at 37 °C. The next morning, cultures were transferred to 30 °C and grown to an optical density at 600 nm (OD_600 nm_) of 0.8, shaking at 200 rpm. Isopropyl β-d-1-thiogalactopyranoside (IPTG; 80 µM) was added to each flask to induce the expression of Sld2 for 21 h at 30 °C at 200 rpm. Subsequently, cells were pelleted by centrifugation at 4,000*g* for 10 min at 4 °C, flash-frozen and stored at −80 °C.

Cells were thawed and resuspended in 100 ml buffer D (25 mM HEPES-KOH pH 7.5, 800 mM KCl, 10% v/v glycerol, 1 M sorbitol, 2 mM ATP, 10 mM MgCl_2_, 0.02% v/v NP-40, 0.1% w/v Tween-20, 1 mM DTT) with two cOmplete EDTA-free protease inhibitor tablets (Merck) and 0.7 mM PMSF, then sonicated on ice for 2 min (5-s pulse-on, 5-s pulse-off). The lysate was clarified by centrifugation in a JA-25.50 rotor at 18,000 rpm for 20 min at 4 °C, and the supernatant was applied onto a gravity column packed with 1 ml Strep-Tactin XT Superflow High Capacity Resin equilibrated in buffer D. The resin was washed with 75 ml buffer D followed by 25 ml buffer E (25 mM HEPES-KOH pH 7.5, 500 mM NaCl, 10% v/v glycerol, 0.02% w/v NP-40, 1 mM EDTA and 1 mM DTT), after which Sld2 was eluted by passing 10× 1 ml buffer E supplemented with buffer BXT through the resin. The highest-concentration fraction was identified by SDS–PAGE (Coomassie staining) and dialysed in buffer F (25 mM HEPES-KOH pH 7.5, 700 mM KOAc, 40% v/v glycerol, 0.02% w/v NP-40, 1 mM EDTA and 1 mM DTT) for 4 h at 4 °C. Sld2 was aliquoted at approximately 0.8 mg ml^−1^ and flash-frozen in liquid nitrogen. For the phosphomimetic Sld2(8D) variant, aspartate substitutions were introduced at the following residues: threonine 84, serine 100, serine 128, serine 138, threonine 168, serine 172, serine 188 and serine 208.

### Cloning, expression and purification of ALFA-tagged Pol ε

Codon-optimized *S. cerevisiae* Pol2-3×Flag, Dpb2, Dpb3 and Dpb4-ALFA were subcloned into GoldenBac shuttle vectors and assembled into a co-expression plasmid, pGB-dest-PolE, as described above for Sld3/7. Similarly, electrocompetent EMBacY cells were transformed with pGB-dest-PolE to prepare bacmids and generate a P1 baculovirus as described above. One billion Sf21 insect cells were seeded in 1 l Sf-900 III SFM medium and infected with 0.5% v/v P1 baculovirus, incubated at 27 °C at 120 rpm and collected 48 h after cell-cycle arrest by centrifugation at 180*g* at 4 °C for 15 min. The cell pellets were flash-frozen in liquid nitrogen and stored at −80 °C. To purify ALFA-tagged Pol ε, the cell pellets were resuspended in 50 ml buffer G (25 mM HEPES-KOH pH 7.6, 400 mM KOAc, 10% v/v glycerol and 2 mM DTT) supplemented with one cOmplete EDTA-free protease inhibitor tablet (Merck), and lysed by sonication on ice for 2 min (1-s pulse-on, 4-s pulse-off). The lysate was clarified by ultracentrifugation at 45,000 rpm for 45 min at 4 °C in a Ti45 rotor, and the supernatant was passed twice through a column packed with 1 ml anti-Flag M2 affinity gel equilibrated in buffer G. The column was washed with 150 ml buffer G and 20 ml buffer G + 2 mM ATP and 10 mM Mg(OAc)_2_. Protein was eluted by incubating the resin three times in 5 ml buffer G + 0.5 mg ml^−1^ 3× Flag peptide for 5 min and collecting the flow-through. The eluate was loaded onto a 5-ml Heparin HP column (Cytiva) and eluted over 25 column volumes with a linear gradient of 400–1,000 mM KOAc in buffer G. Fractions containing Pol ε were concentrated and gel-filtered onto a HiLoad 16/60 Superdex 200-pg column (Cytiva). Pol ε was concentrated to approximately 1 mg ml^−1^, aliquoted and flash-frozen in liquid nitrogen.

### Cloning, expression and purification of Sic1

T7 Express cells (NEB) were transformed with hexahistidine-tagged *S. cerevisiae* Sic1^73^. Transformant colonies were incubated overnight in 100 ml LB supplemented with 100 µg ml^−1^ carbenicillin at 37 °C shaking at 200 rpm. Two litres of LB medium and 100 µg ml^−1^ carbenicillin were inoculated with 1% v/v dense overnight culture and grown to an OD_600 nm_ of 0.6 at 37 °C and 200 rpm. Expression of Sic1 was induced by adding 0.5 mM IPTG, after which the cultures were incubated for 3 h at 37 °C at 200 rpm. Cells were collected by centrifugation at 4,000*g* for 10 min at 4 °C, and the cell pellet was flash-frozen in liquid nitrogen and stored at −80 °C.

The pellet was dissolved in 100 ml buffer H (25 mM HEPES-KOH pH 7.5, 500 mM NaCl, 10% v/v glycerol, 1 mM EGTA, 0.2% w/v Triton X-100, 0.5 mM TCEP, 10 mM Imidazole) with two cOmplete EDTA-free protease inhibitor tablets (Merck) and 0.7 mM PMSF in a beaker. Lysozyme (0.2 mg ml^−1^) was added and incubated with the cell suspension for 10 min while stirring. Cells were then sonicated on ice for 5 min (2-s pulse-on, 5-s pulse-off), and the debris was removed by centrifugation at 20,000 rpm in a JA-25.50 rotor for 30 min at 4 °C. Two millilitres of Ni-NTA beads (QUIAGEN) were equilibrated in buffer H and rotated with the clarified lysate for 1 h at 4 °C. Subsequently, beads were collected in a gravity column and washed with 150 ml buffer H and 20 ml buffer H supplemented with 2 mM ATP and 10 mM MgCl_2_. Protein was eluted with 15 ml buffer H supplemented with 250 mM imidazole, concentrated and gel-filtered on a HiLoad 16/60 Superdex 75-pg column (Cytiva) equilibrated in buffer I (25 mM HEPES-KOH pH 7.5, 5% v/v glycerol, 5 mM MgCl_2_, 0.5 mM EDTA and 0.5 mM TCEP) supplemented with 200 mM NaCl. Gel filtration did not yield pure Sic1. Consequently, peak fractions were loaded onto a 1-ml Mono S column (Cytiva) and washed with 10 column volumes of buffer I supplemented with 50 mM NaCl. Sic1 was eluted with a linear gradient of 50–1,000 mM NaCl in buffer I over 30 column volumes, and fractions containing pure Sic1 were dialysed at 4° C in buffer I + 200 mM NaCl for 3 h under agitation. Sic1 was concentrated to 10.8 mg ml^−1^ and flash-frozen in liquid nitrogen.

### Cloning, expression and purification of Twin-Strep-tagged SUMO-Mcm10

*S. cerevisiae* Mcm10 was cloned in-frame with an N-terminal 10×His-SUMO cassette and a C-terminal Twin-Strep-tag, and transformed into Rosetta 2 pLysS cells. Multiple colonies were picked and grown overnight at 37 °C in 100 ml LB supplemented with 100 µg ml^−1^ carbenicillin and 33 µg ml^−1^ chloramphenicol. Then, 6× 1 l LB supplemented with 100 µg ml^−1^ carbenicillin and 33 µg ml^−1^ chloramphenicol were each inoculated with 10 ml dense overnight culture and grown to an OD_600 nm_ of 0.7 at 37 °C and 200 rpm. Overexpression was induced by the addition of 0.5 mM IPTG and continued for 16 h at 16 °C. Cells were collected by centrifugation at 4,000*g* for 10 min at 4 °C, flash-frozen in liquid nitrogen and stored at −80 °C. The cell pellet was resuspended in 230 ml buffer J (25 mM HEPES-KOH pH 7.6, 500 mM NaCl, 10% v/v glycerol, 1 mM EDTA, 0.05% w/v Tween-20 and 1 mM DTT) supplemented with four cOmplete EDTA-free protease inhibitor tablets (Merck), then lysed by sonication on ice for 5 min (2-s pulse-on, 5-s pulse-off). The lysate was clarified by centrifugation in a JA-25.50 rotor at 20,000 rpm for 30 min at 4 °C, and the supernatant was loaded onto a 1-ml cOmplete His-Tag Purification column (Merck) installed in tandem with a 1-ml Strep-Tactin XT 4Flow High Capacity column (IBA), both pre-equilibrated in buffer J. The columns were washed with buffer J until the absorbance at 280 nm returned to its baseline signal, after which protein was eluted from the His-Tag Purification column into the connected Strep-Tactin XT column with 9 ml buffer J supplemented with 200 mM imidazole. The His-Tag Purification column was disconnected, the Strep-Tactin XT column was washed with 9 ml buffer B, and this was finally followed by elution with 9 ml buffer K (25 mM HEPES pH 7.6, 300 mM NaCl, 10% v/v glycerol, 0.05% w/v Tween-20, 1 mM DTT and 5 mM desthiobiotin). Mcm10-containing fractions were pooled and dialysed overnight into buffer L (25 mM HEPES pH 7.6, 200 mM NaCl, 20% v/v glycerol, 0.05% w/v Tween-20, 1 mM EDTA and 2 mM DTT) at 4 °C while stirring. Mcm10 was aliquoted at a concentration of approximately 0.5 mg ml^−1^ and flash-frozen in liquid nitrogen.

### Preparation of an MH-conjugated ARS1 DNA template

A 168-bp DNA template containing the *S. cerevisiae* origin of replication ARS1, flanked by two MH recognition sites, was generated by PCR and purified as previously described^74,75^. The DNA template was covalently tethered to either Twin-Strep-tagged MH or tandem ALFA/Twin-Strep-tagged MH using previously established protocols^30^.

#### S-CDK prephosphorylation of Sld3/7

Sld3/7 prephosphorylation by S-CDK was modified from previous protocols^24^. In brief, 900 nM Twin-Strep-tagged Sld3/7 was phosphorylated by 100 nM S-CDK in buffer M (40 mM HEPES-KOH pH 7.5, 310 mM potassium glutamate, 10 mM Mg(OAc)_2_, 10% v/v glycerol, 0.02% w/v NP-40, 1 mM DTT, 2 mM ATP and 0.4 mg ml^−1^ BSA) in a total volume of 100 µl for 8 min at 24 °C and 1,250 rpm. Phosphorylation was stopped by adding 2.2 µM Sic1 to the reaction and incubating it for 2 min at 24 °C and 1,250 rpm. Then, 500 mM KCl was added to the reactions, which were then bound to 10 µl MagStrep ‘type3’ XT slurry (IBA) equilibrated in buffer N (25 mM HEPES-KOH pH 7.5, 500 mM KCl, 5 mM Mg(OAc)_2_, 10% v/v glycerol, 0.02% w/v NP-40 and 1 mM DTT). After 30 min of incubation at 24 °C and 1,250 rpm, beads were washed five times with 200 µl buffer K, and ppSld3/7 was eluted in 10 µl buffer N + 25 mM d-biotin for 10 min at 24 °C and 1,250 rpm, aliquoted, flash-frozen in liquid nitrogen and stored at −80 °C. Successful phosphorylation and approximate yield were estimated by SDS–PAGE. For cryo-EM experiments, ppSld3/7 was prepared in parallel and not flash-frozen before use.

### Rad53 prephosphorylation of Sld3/7

Rad53-prephosphorylated Sld3/7 was prepared by incubating 700 nM Twin-Strep-tagged Sld3/7 with 356 nM Rad53 in 100 µL buffer M for 30 minutes at 30 °C and 1,250 rpm. Subsequently, Rad53-prephosphorylated Sld3/7 was purified by Strep-Tactin XT affinity purification exactly as described above for CDK-prephosphorylated Sld3/7.

### In vitro ATP–dCMGE assembly

ATP-bound dCMGE complexes were assembled as previously described^30,47^ with minor modifications. First, MCM-DHs were loaded for 30 min at 30° C and 1,250 rpm by co-incubating 20 nM MH-conjugated ARS1 with 52 nM ORC, 52 nM Cdc6 and 110 nM Mcm2–7–Cdt1 in 100 µl buffer O (25 mM HEPES-KOH pH 7.5, 100 mM potassium glutamate, 10 mM Mg(OAc)_2_, 1 mM ATP and 0.02% w/v NP-40). Afterwards, loaded MCM-DHs were phosphorylated with 80 nM DDK at 24 °C and 1,250 rpm for 10 min, then bound for 30 min at 24 °C and 1,250 rpm to 5 µl MagStrep ‘type3’ XT slurry equilibrated in buffer P (25 mM HEPES-KOH pH 7.5, 100 mM potassium glutamate, 10 mM Mg(OAc)_2_ and 0.02% w/v NP-40). The beads were washed three times with 200 µl buffer Q (25 mM HEPES-KOH pH 7.5, 500 mM NaCl, 5 mM Mg(OAc)_2_ and 0.02% w/v NP-40) and once with 200 µl buffer P, then eluted in 20 µl buffer O + 25 mM d-biotin for 15 min at 24 °C and 1,250 rpm. S-CDK (200 nM) was added to DDK-phosphorylated MCM-DHs, and dCMGE assembly was then started by further addition of 40 nM Dpb11, 32.5 nM Pol ε, 133 nM GINS, 106 nM Cdc45, 40 nM Sld3/7 and 67 nM Sld2. After 12 min of incubation at 30 °C and 1,250 rpm, the reactions were immediately used for negative-stain grid preparation. Wild-type proteins were substituted with mutant constructs or omitted as specified. To split dCMGEs into sCMGEs, 100 nM Mcm10 and 300 nM RPA were added at the same time as the other firing factors.

### In vitro pre-IC assembly

To assemble the pre-IC, DDK-phosphorylated MCM-DHs were prepared as described above, but eluted in the absence of ATP. Sld3/7 and Sld2 were replaced by equimolar amounts of ppSld3/7 and phosphomimetic Sld2(8D), and no CDK was added. DDK-phosphorylated DHs were incubated with ppSld3/7, Sld2(8D), Dpb11, Pol ε, GINS and Cdc45 in the absence of ATP and CDK for 10 min at 24 °C and 1,250 rpm, after which reactions were either negatively stained, applied to cryo-EM grids and plunge-frozen using a Vitrobot, or further purified.

### ALFA pull-down of pre-IC and dCMGE complexes

To purify either pre-IC or dCMGE, phospho-DH formation and respective complex assembly was done on an ALFA-tagged 2×MH-ARS1 DNA template. Twenty microlitres of assembly reaction prepared as described above was added to 15 µl ALFA PE Selector slurry (NanoTag Biotechnologies) equilibrated in buffer P and bound for 5 min at 24 °C and 1,250 rpm.

For pre-IC maturation reactions, beads were washed with 3× 150 µl buffer P and eluted for 5 min at 24° C and 1,250 rpm in 20 µl buffer P + 200 µM ALFA peptide (NanoTag Biotechnologies). Pre-IC to dCMGE conversion was triggered by adding 1 mM ATP per reaction and analysed by nsEM.

To assay for the high-salt stability of the pre-IC and dCMGE, beads were washed with 3× 150 µl of buffer R (25 mM HEPES-KOH pH 7.5, 5 mM Mg(OAc)_2_, 10% v/v glycerol and 0.02% w/v NP-40) and either 250 mM potassium glutamate (low-salt wash) or 300 mM KCl (high-salt wash). Elution was performed in 20 µl buffer P + 200 µM ALFA peptide (no nucleotide for pre-IC reactions; 1 mM ATP for dCMGE reactions) for 5 min at 24 °C and 1,250 rpm.

### In vitro DNA replication assay

DNA replication was performed at 30 °C and 1,250 rpm following previous protocols^76^. In brief, MCM-DHs were loaded for 20 min using 40 nM ORC, 40 nM Cdc6, 60 nM Mcm2–7–Cdt1 and 4 nM 10.6 kb pJY22 plasmid template DNA in buffer S (25 mM HEPES-KOH pH 7.6, 100 mM potassium glutamate, 10 mM magnesium acetate, 5 mM ATP, 0.02% NP-40-S and 2 mM DTT). Loaded MCMs were phosphorylated with 50 nM DDK for 15 min. DNA replication was performed for 30 min by adding final concentrations of 40 nM Dpb11, 20 nM Pol ε, 20 nM GINS, 80 nM Cdc45, 20 nM CDK, 25 nM Sld3/7, 50 nM Sld2, 200 nM RPA, 20 nM TopoI, 50 nM Pol α, 20 nM Mcm10, 200 μM each of CTP, GTP and UTP, 80 μM dNTP and 33 nM ɑ^32^P-dCTP. When specified, Dpb11, Sld3/7 and Sld2 proteins were excluded from reactions or substituted by mutant versions. In the CDK bypass experiment, reactions contained either 10 nM wild-type Sld2 and 5 nM Sld3/7 or 50 nM Sld2(8D) and 25 nM phosphorylated Sld3/7.

Reactions were stopped with 85 mM EDTA, cleared over an Illustra MicroSpin G-50 column, denatured in 2% sucrose, 0.02% bromophenol blue, 60 mM NaOH and 10 mM EDTA, separated on 0.8% agarose gels in alkaline conditions containing 30 mM NaOH and 2 mM EDTA at approximately 1 V per cm for 17 h, fixed in cold 5% trichloroacetic acid, dried, exposed to phosphor screens and scanned using a Typhoon phosphor imager.

### Sample preparation and data collection for nsEM

Carbon-coated 300-mesh copper grids (EM Resolutions) were glow-discharged at 25 mA for 1 min in a GloQube Plus (Quorum) in ambient air. Four-microlitre samples were incubated for 2 min on a glow-discharged grid, blotted and negatively stained by two applications of 4 µl 2% uranyl-acetate for 20 s, after which the grid was blotted dry. nsEM micrographs were acquired using a Rio16 camera (Gatan Digital Micrograph) on a FEI Tecnai G2 Spirit Twin microscope operated at 120 kV. Approximately 50–150 micrographs were collected per dataset at 3.1 Å per pixel (px) (29,000× magnification) at −1 to −2 µm defocus.

### nsEM image processing

Micrographs were imported in RELION-4^77^ and contrast transfer function (CTF) was estimated using Gctf^78^. MCM-containing particles were picked using crYOLO (v. 1.9.2)^79^, imported into RELION and extracted with a 144-px box size. Extracted particles were 2D-classified in either RELION-4 or cryoSPARC (v.4.4.1)^80^. Interpretable class averages were categorized (DH, pre-IC, *cis*- or *trans*-dCMGE, sCMGE) and particles were quantified. Complex assembly efficiency was calculated as the number of all detected target particles divided by the number of all licensed replication origins. For example, the number of pre-IC particles was divided by the sum of DHs and pre-IC particles. For Mcm10-dependent CMGE splitting experiments, all sCMGEs were multiplied by a factor of 0.5, to account for the fact that sCMGEs originate from a single, licensed replication origin.

### Cryo-EM sample preparation for the pre-IC

MCM-DHs were loaded onto an ALFA-tagged 2×MH-ARS1 template and phosphorylated by DDK as described above. One hundred microlitres of DNA-loaded, phospho-DHs were purified on 15 µl ALFA PE Selector beads for 10 min at 24 °C and 1,250 rpm. Subsequently, beads were washed three times with 200 µl buffer Q and once with 200 µL buffer O and eluted for 10 min in 20 µl buffer O supplemented with 200 µM ALFA peptide at 24 °C and 1,250 rpm. Given that ALFA elution yielded a higher number of phospho-DHs than Strep-Tactin XT purification did, the eluted phospho-DHs were incubated with a four times higher molar amount of firing factors (Dpb11, Pol ε, GINS, Cdc45, ppSld3/7 and Sld2(8D)) at 24 °C and 1,250 rpm for 10 min.

Graphene-oxide-coated 300-mesh UltrAuFoil R1.2/R1.3 grids were prepared on the day according to a previously published protocol^47^. Four microlitres of sample was applied per grid in a Mark IV Vitrobot (FEI), followed by incubation for 60 s at 24 °C and 90% humidity. Grids were blotted for 3–4.5 s at blot force 0 and plunge-frozen in liquid ethane.

### Cryo-EM data collection for the pre-IC

A total of 59,347 movies were collected on a 300-kV FEI Titan Krios G3i at a nominal magnification of 130,000× (0.95 Å px^−1^ physical pixel size) using a Falcon IV direct electron detector in counting mode and a Selectris energy filter with a slit width of 10 eV using EPU v.3.2. Three shots were acquired per hole at spot size 9 with a beam diameter of 660 nm, a 100-µm objective aperture inserted and a defocus range from −2.0 to −3.0 µm. Each movie was recorded with 1,674 electron event representation (EER) frames for 5.44 s with a total fluence of 39 electrons per Å^2^ (Extended Data Table 1).

### Cryo-EM image processing for the pre-IC

A total of 59,347 EER movies were aligned and dose-weighted with 5 × 5 patches using RELION’s own implementation of MotionCor2^81^. Fifty-four internal frames were grouped into 31 fractions resulting in a dose per frame of 1.26 electrons per Å^2^. Motion-corrected micrographs were imported into cryoSPARC (v.4.4.1)^80^ and CTF estimation was done using Patch CTF. Initial particle picking was performed using Blob Picker with a diameter range of 200–350 Å and circular blobs. A total of 5,149,760 particles were extracted, fourfold binned to 3.8 Å px^−1^ with a 150-px box size and cleaned up with multiple rounds of reference-free 2D classification with 400 classes and an uncertainty factor of 2. A total of 62,192 MCM-DH and pre-IC particles were selected from 2,867 micrographs containing more than 20 particles per micrograph in a defocus range from −1.5 to −2.5 µm, and used to train a Topaz network with 75 expected particles per micrograph^82^. After Topaz picking at an extraction threshold of −6 with an extraction radius of 28 px, 1,572,464 particles were extracted and Fourier-cropped to 3.8 Å px^−1^ with a box size of 150 px and subjected to 3 rounds of 2D classification yielding 302,557 DHs and 345,093 pre-ICs.

Ab-initio reconstructions of both DHs and pre-ICs were generated independently in *C*1. The initial volumes of each complex were used as 3D references for heterogenous refinement to further separate DHs and pre-ICs from each other and remove low-quality particles. A total of 279,730 DH particles were separated into 20 classes by alignment-free 3D classification in cryoSPARC. A total of 162,764 high-quality DH particles were selected, unbinned to 0.95 Å px^−1^ and refined to a final resolution of 2.8 Å with *C*2 symmetry applied. After separating pre-IC particles from DH particles through heterogenous refinement, 335,896 binned pre-IC particles were non-uniform-refined with *C*2 symmetry applied, unbinned to 0.95 Å px^−1^ with a 600-px box size and 3D-classified without alignment, yielding 290,496 particles that were non-uniform-refined to 3.72 Å. This first unbinned reconstruction of the pre-IC was subjected to further classification in cryoSPARC to remove low-quality particles, resulting in a stack of 151,175 pre-IC particles exhibiting improved density quality. After homogenous refinement, symmetry expansion was performed. Local *C*1 refinement yielded a 3.4-Å-resolution map of the pre-IC dimer, with some residual anisotropy visible. To improve the reconstruction further, refinement of a signal-subtracted monomer was performed. To generate the best possible mask for subtraction, a double-subtraction strategy was implemented. First, one asymmetric unit was locally refined using a soft mask around the top pre-IC monomer. The resulting, improved, reconstruction was used to generate a new soft mask for signal subtraction, which was used to accurately remove the signal from the top pre-IC monomer. The remaining pre-IC monomer was reconstructed and locally refined to 3.3-Å resolution, and showed improved isotropy. This map was used to generate a third soft mask for a second signal subtraction of the bottom monomer. This allowed us to determine the structure of the top monomer using local refinement to a resolution of 3.2 Å.

### Cryo-EM sample preparation for phospho-DH-3745

DDK-phosphorylated, DNA-loaded MCM-DHs were eluted from MagStrep ‘type3’ XT beads in buffer T (25 mM HEPES-KOH pH 7.5, 100 mM KOAc, 0.02% w/v NP-40 and 25 mM d-biotin) and incubated with 32 nM (yeast-expressed) Sld3/7 and 127 nM Cdc45 for 10 min at 30 °C and 1,250 rpm. To stabilize the phospho-DH-3745 complex, the sample was cross-linked with 0.05% w/v glutaraldehyde for 5 min and quenched with 25 mM Tris-HCl pH 7.5. For the first dataset, the reaction was vitrified without further purification as described for the pre-IC. For the second and third dataset, the reaction was purified after cross-linking through ALFA pull-down. Five cross-linked and quenched reactions were pooled and bound to 1.5 µl ALFA Selector PE resin through the ALFA-tagged 2×MH-ARS1 DNA template for 1 h at 24 °C and 1,250 rpm, washed once with 50 µl buffer O and eluted for 30 min at 24 °C and 1,250 rpm in 12 µl buffer O + 200 µM ALFA peptide. Cryo-EM grids were prepared as described for the pre-IC.

### Cryo-EM data collection for phospho-DH-3745

Three datasets of 31,794 (dataset 1), 32,200 (dataset 2) and 41,658 (dataset 3) movies were acquired on a 300-kV FEI Titan Krios G3i using a Gatan K2 Summit direct electron detector in counting mode and a BioQuantum energy filter with a slit width of 20 eV at a nominal magnification of 130,000× (1.08 Å px^−1^) using EPU v.3.2. Per hole, two shots were recorded with a 100-µm objective aperture inserted, a defocus range from −1.1 to −2.5 µm and a total fluence of 49.1–50.4 electrons per Å^2^.

### Cryo-EM image processing for phospho-DH-3745

Each dataset was preprocessed separately. First, cryo-EM movies were motion-corrected in RELION-4 using its own implementation of MotionCor2^81^ and CTF estimation was done with Gctf^78^. A Topaz picking network^82^ was iteratively trained on the first dataset using a selection threshold of −3, a scale factor of 8 and 30 expected particles per micrograph. Picked particles were extracted, 2× binned (2.16 Å px^−1^) with a 360-px box size and cleaned up by reference-free 2D classification. Noise and low-quality averages were discarded. The remaining particles were used as input for the next round of Topaz training. Ultimately, particles were unbinned (448-px box size) and transferred to cryoSPARC^80^ to generate an initial volume, and subjected to non-uniform refinement with *C*2 symmetry applied. A total of 100,495 particles from dataset 1, 184,975 particles from dataset 2 and 239,120 particles from dataset 3 were then joined for downstream processing, yielding 524,590 particles. After another round of 2D classification, 359,025 particles underwent 2 rounds of CTF refinement (beam-tilt, anisotropic magnification, per-particle defocus and per-micrograph astigmatism) and Bayesian polishing in RELION^83^, resulting in a 3.1-Å reconstruction of the consensus phospho-DH bound to Sld3 after non-uniform refinement in cryoSPARC with *C*2 symmetry applied.

To isolate Cdc45-bound phospho-DHs, particles were first *C*2-symmetry-expanded in cryoSPARC. An AlphaFold-Multimer^84^ prediction of an Sld3-CBD–Cdc45 complex was aligned with the map of the phospho-DH by docking an atomic model of a CMG ring^30^ (PDB: 7QHS) into one MCM ring and superimposing the prediction via Cdc45. A volume of the aligned Sld3-CBD–Cdc45 prediction was generated at 10-Å resolution through the ‘molmap’ command in ChimeraX^85^ and used as input to prepare a soft mask in RELION. Using this mask, 718,050 *C*2-symmetry-expanded phospho-DH particles were subjected to 2 rounds of focused 3D classification without alignment and a *T*-value of 20 into 8 classes. A total of 72,693 particles exhibiting proteinaceous features inside this mask were selected and refined in RELION in *C*1 local searches restricted to 1.8° while masking out Sld3-CBD–Cdc45 signal from the opposite MCM hexamer. Subsequently, the masked out Sld3-CBD–Cdc45 density was signal-subtracted in RELION. The Cdc45-bound DH was then imported into cryoSPARC and locally refined, yielding a final reconstruction at 3.7 Å.

In parallel, *C*2-symmetry-expanded Sld3-bound phospho-DHs were subjected to signal subtraction within cryoSPARC. Cdc45 and Sld3-CBD density associated with one of the two MCM hexamers was masked out and subtracted. A total of 718,050 particles of the remaining phospho-DH bound to a single Sld3-CBD–Cdc45 complex were subjected to 2 rounds of 3D classification without alignment in cryoSPARC. Ten classes were chosen, with a class similarity of 0.25, and resolution was limited to 15 Å. A total of 96,027 particles were selected, from 3D classes displaying featured Cdc45 density, and locally refined using *C*1 symmetry in cryoSPARC to a resolution of 3.5 Å. This approach yielded a map of the Sld3–Cdc45-bound phospho-DH, also featuring Sld7 density.

### Cryo-EM sample preparation for sCMGE assembled with Sld2 and RPA

sCMGE complexes on double-roadblocked ARS1 DNA (168 bp) were prepared essentially as described above (‘In vitro ATP–dCMGE assembly’). After dCMGE splitting, 20-µl assembly reactions were further purified on paramagnetic ALFA beads as described above (‘ALFA pull-down of pre-IC and dCMGE complexes’). Cryo-EM grids were prepared as described for the pre-IC, with three applications of 4 µl eluate per grid.

### Cryo-EM data collection for sCMGE assembled with Sld2 and RPA

A total of 50,060 movies were collected at a nominal magnification of 130,000× (0.95 Å px^−1^ physical pixel size) on a FEI Titan Krios G3i using a Falcon IV direct electron detector in counting mode and a Selectris energy filter with a slit width of 10 eV using EPU v.3.2. Per hole, three shots were acquired with a defocus range from −2.0 to −2.9 µm and a 100-µm objective aperture inserted. Movies were recorded with 31 frames and a total dose of 38.6 electrons per Å^2^ (Extended Data Table 2).

### Cryo-EM image processing of sCMGE assembled with Sld2 and RPA

A total of 50,060 EER movies were motion-corrected using RELION’s own implementation of MotionCor2^81^ and CTF-estimated with CTFFIND (v.4.1.13)^86^. 1,940 particles were manually picked from 20 micrographs and used as input for Topaz training^82^. Using Topaz, 4,827,716 particles were picked and extracted at 3.8 Å px^−1^ (4× binning) and a 108-px box size, and cleaned up with multiple rounds of 2D classification in cryoSPARC (v.4.4.1)^80^. A subset of clean sCMGE and MCM-DH classes were selected to generate initial 3D models, which were used in heterogenous refinement of a total of 3,801,543 sCMGE and MCM-DH particles. This yielded 1,125,095 sCMGE particles, which were unbinned with a 432-px box and homogeneously refined (with global CTF correction) to a final resolution of 2.7 Å.

### Cryo-EM sample preparation for sCMGE assembled without Sld2 and RPA

sCMGE complexes were assembled as described above, with one notable exception: the omission of Sld2. After dCMGE splitting, 13 20-µl reactions were pooled and jointly purified on 60 µl ALFA beads. Binding and washing steps were performed as described, and DNA-bound complexes were eluted in 50 µl buffer O + 200 µM ALFA peptide. Lacey cryo-EM grids (400-mesh Cu; TAAB) were coated with graphene oxide by first hydrophilizing the grid surface with 4 µl 300 nM DDM and side-blotting, followed by two rounds of on-grid incubation with 4 µl of a 20 µg ml^−1^ graphene oxide suspension in 300 nM DDM. Grids were washed with three 5-µl droplets of Milli-Q H_2_O from the backside, and blotted dry from the backside. Cryo-EM grids were vitrified as described before for the pre-IC, with two applications of 4 µl eluate per grid.

### Cryo-EM data collection for sCMGE assembled without Sld2 and RPA

A total of 70,337 movies were recorded on a FEI Titan Krios G3i at a nominal magnification of 130,000× (0.95 Å px^−1^ physical pixel size) using a Falcon IV direct electron detector in counting mode and a Selectris energy filter with a slit width of 10 eV using EPU v.3.2. Shots were acquired in a 0.7-µm spacing, with a 100-µm objective aperture inserted and a defocus range from −1.4 to −2.4 µm. Per movie, 29 frames were recorded with a total fluence of 42.0 electrons per Å^2^ (Extended Data Table 2).

### Cryo-EM image processing for sCMGE assembled without Sld2 and RPA

A total of 70,337 EER movies were preprocessed using RELION’s own implementation of MotionCor2^81^ and imported into cryoSPARC (v.4.4.1)^80^ for CTF estimation using PatchCTF. A total of 4,579 particles were manually picked from 134 micrographs and used to train a Topaz model^87^ with 75 expected particles per micrograph. A total of 2,390,783 particles were extracted with 8× binning at 7.6 Å per px and a 70-px box size, and subsequently cleaned up with multiple rounds of reference-free 2D classification, removing well-averaging MCM-DHs as well as noise, ultimately yielding 162,691 CMG-like particles. Initial 3D references were generated with clean subsets of sCMGE- and dCMGE-like classes (orange and purple outlines, respectively; Extended Data Fig. 8c) using cryoSPARCs ab-initio reconstruction. These volumes were subsequently used to separate sCMGEs from contaminating dCMGE-like particles through multiple rounds of heterogenous refinement (*C*1 symmetry; one sCMGE reference and two dCMGE references). A total of 72,370 cleaned-up sCMGE particles were then unbinned in RELION-5.0 with a box size of 512 px, subjected to 2 rounds of Bayesian polishing and 3 rounds of CTF refinement (each round correcting separately: fourth-order aberrations, tilt and trefoil; anisotropic magnification; per-particle defocus and per-micrograph astigmatism) and 3D-refined using Blush^88^ to a nominal resolution of 3.4 Å.

### Atomic model building and refinement

The structure of the pre-IC complex was built to a locally refined monomeric pre-IC cryo-EM map density-modified with EMReady^89^. A single CMGE complex extracted from PDB 7PMK (ref. ^48^) was first docked into the cryo-EM density. After this initial placement, individual domains were then docked as rigid bodies into the cryo-EM density using UCSF Chimera^90^. Because they matched the cryo-EM density closely, the structure predictions of Dpb11–Mcm7, Dpb11–GINS, Cdc45–Sld3, Mcm4–Sld3 and Mcm7–Sld7 generated with AlphaFold 3^37^ were also used as starting models for building these interaction interfaces. Each chain or pair of chains was flexibly fit after generating self-restraints in Coot^91^ using density maps of varying blurring. Fragments mapping outside the visible density were deleted. The entire model was then manually adjusted with real-space refinement in Coot, using varying whole-molecule restraints depending on the local quality of the density. Automated real-space refinement was then performed in PHENIX (v.1.21)^92^ against the non-postprocessed map.

The pre-IC dimer complex structure was built to a *C*2-symmetric cryo-EM map density modified with EMReady. First, two refined pre-IC monomers were rigid-body-docked into the cryo-EM density map in ChimeraX. DNA chains from each monomer were trimmed at the overlapping region and were merged. Finally, the protein–protein interface between the monomers was adjusted using real-space refinement in Coot. Automated real-space refinement was then performed in PHENIX (v.1.21)^92^ against the non-postprocessed map.

The DH–Sld3-MBD and DH-3745 complex was built starting from a previously published MCM-DH structure bound to duplex DNA (PDB: 7P30; ref. ^14^), combined with the structure predictions of Mcm4–Sld3, Sld7–Mcm6 and Cdc45–Sld3 produced using AlphaFold 2 (v.2.3.4)^84^. Initial docking was performed in ChimeraX^93^. The density fit of protein regions except zinc fingers was first adjusted using molecular-dynamics-based real-space refinement in Isolde^94^. Next, the positions of all atoms were adjusted with flexible fitting and real-space refinement (sphere refinement) in Coot^91^. Fragments mapping outside the visible density were truncated from the atomic coordinate file. Automated real-space refinement was performed against the non-postprocessed map.

The structure of sCMGE assembled on ARS1 DNA with RPA and Sld2 was modelled into a cryo-EM map density modified with EMReady, using PDB 7PMK (ref. ^48^) as an initial template. The starting model was rigid-body-docked in UCSF Chimera, followed by flexible fitting of individual chains in Coot using chain restraints. Where density permitted, the model was expanded manually or guided by AlphaFold 2 predictions. Because the resolution was insufficient for base identification, an arbitrary repetitive DNA sequence was modelled. To account for the bases that stretch between the modelled double-stranded and single-stranded DNA stretches and could not be built owing to poor density, we maintained the nucleotide numbering from PDB 6SKL (ref. ^95^). Final automated real-space refinement was performed using PHENIX (v.1.21) against the non-postprocessed map. For all structures, the quality of the resulting atomic models was evaluated with MolProbity^96^ (Extended Data Tables 1 and 2). Structures in figures are displayed using EMReady-postprocessed maps.

### Reporting summary

Further information on research design is available in the Nature Portfolio Reporting Summary linked to this article.

## Online content

Any methods, additional references, Nature Portfolio reporting summaries, source data, extended data, supplementary information, acknowledgements, peer review information; details of author contributions and competing interests; and statements of data and code availability are available at 10.1038/s41586-026-10657-7.

## Supplementary information

Supplementary Information Supplementary Results, Supplementary Figures, Supplementary Tables and Supplementary References

Reporting Summary

Peer Review file

## Figures and Tables

**Extended Data Table 1:**
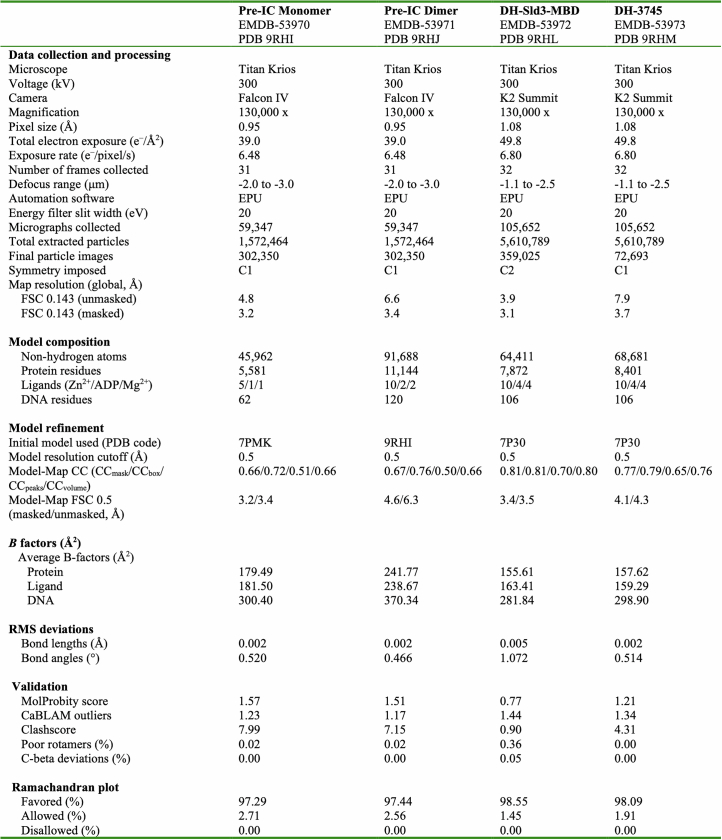
Cryo-EM data collection, refinement and validation statistics

Information for the structures of pre-IC Monomer, pre-IC Dimer, DH–Sld3-MBD and DH-3745.

**Extended Data Table 2:**
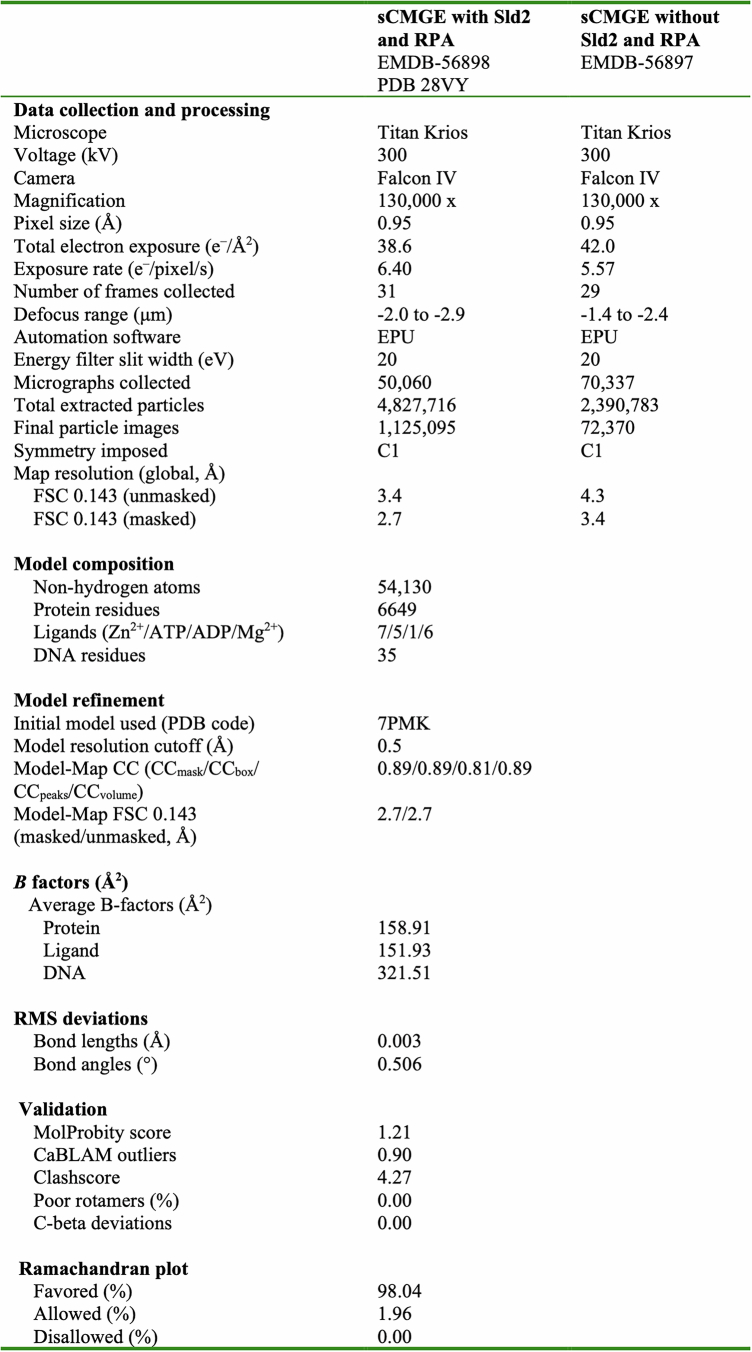
Additional cryo-EM data collection, refinement and validation statistics

Information for the structures of sCMGE assembled with or without Sld2 in the presence of RPA.

## Data Availability

Data supporting the findings of this study are available within the paper and its Supplementary Information. Cryo-EM density maps have been deposited in the Electron Microscopy Data Bank (EMDB) under the following accession codes: EMD-53972 (DH–Sld3-MBD), EMD-53973 (DH–Sld3/7–Cdc45), EMD-53971 (dimeric pre-IC), EMD-53970 (monomeric pre-IC), EMD-56898 (sCMGE assembled with Sld2 and RPA), EMD-56897 (sCMGE assembled with RPA and without Sld2). Atomic coordinates have been deposited in the PDB under the following accession codes: 9RHL (DH–Sld3-MBD), 9RHM (DH–Sld3/7–Cdc45), 9RHJ (dimeric pre-IC), 9RHI (monomeric pre-IC) and PDB 28VY (sCMGE assembled with Sld2 and RPA).
