# Supplementary material for: Structure of the pre-initiation complex explains CMGE biogenesis
Source: Nature. 2026 Jun 17;655(8125):1330–8. doi: 10.1038/s41586-026-10657-7 (PMC13421386; doi:10.1038/s41586-026-10657-7)
Supplement: Supplementary file 2 — Reporting Summary [file 41586_2026_10657_MOESM2_ESM.pdf]

nature portfolio

Corresponding author(s): Alessandro Costa

Last updated by author(s): May 11, 2026

Reporting Summary

Nature Portfolio wishes to improve the reproducibility of the work that we publish. This form provides structure for consistency and transparency in reporting. For further information on Nature Portfolio policies, see our Editorial Policies and the Editorial Policy Checklist.

Statistics

For all statistical analyses, confirm that the following items are present in the figure legend, table legend, main text, or Methods section.

n/a	Confirmed	
☐	☒	The exact sample size (*n*) for each experimental group/condition, given as a discrete number and unit of measurement
☐	☒	A statement on whether measurements were taken from distinct samples or whether the same sample was measured repeatedly
☐	☒	The statistical test(s) used AND whether they are one- or two-sided<br>*Only common tests should be described solely by name; describe more complex techniques in the Methods section.*
☒	☐	A description of all covariates tested
☒	☐	A description of any assumptions or corrections, such as tests of normality and adjustment for multiple comparisons
☐	☒	A full description of the statistical parameters including central tendency (e.g. means) or other basic estimates (e.g. regression coefficient) AND variation (e.g. standard deviation) or associated estimates of uncertainty (e.g. confidence intervals)
☐	☒	For null hypothesis testing, the test statistic (e.g. *F*, *t*, *r*) with confidence intervals, effect sizes, degrees of freedom and *P* value noted<br>*Give P values as exact values whenever suitable.*
☒	☐	For Bayesian analysis, information on the choice of priors and Markov chain Monte Carlo settings
☒	☐	For hierarchical and complex designs, identification of the appropriate level for tests and full reporting of outcomes
☒	☐	Estimates of effect sizes (e.g. Cohen's *d*, Pearson's *r*), indicating how they were calculated

Our web collection on statistics for biologists contains articles on many of the points above.

Software and code

Policy information about availability of computer code

Data collection	Gatan DigitalMicrograph and ThermoFisher EPU v3.2.
Data analysis	Topaz v0.2.5, crYOLO 1.9.2, MotionCor2, Gctf v1.06, CTFFIND v4.1.13, RELION v4.0 and v5.0, cryoSPARC v4.4.1, UCSF ChimeraX v1.8 and 1.9, ISOLDE v1.6.0, Coot v0.9.8.1 EL, Phenix v1.21., AlphaFold v2.3.4 AlphaFold 3

For manuscripts utilizing custom algorithms or software that are central to the research but not yet described in published literature, software must be made available to editors and reviewers. We strongly encourage code deposition in a community repository (e.g. GitHub). See the Nature Portfolio guidelines for submitting code & software for further information.

Data

Policy information about availability of data

All manuscripts must include a data availability statement. This statement should provide the following information, where applicable:

- Accession codes, unique identifiers, or web links for publicly available datasets
- A description of any restrictions on data availability
- For clinical datasets or third party data, please ensure that the statement adheres to our policy

Data supporting the findings of this study are available within the paper and its supplementary information files. Cryo-EM density maps have been deposited in the Electron Microscopy Data Bank (EMDB) under the following accession codes: EMD-53972 (maps for the DH-Sld3-MBD), EMD-53973 (maps for the DH-Sld3/7-Cdc45), EMD-53971 (maps for the dimeric pre-IC), EMD-53970 (maps for the monomeric pre-IC), EMDB-56898 (maps for sCMGE assembled with Sld2 and RPA), EMD-56897 (maps for sCMGE assembled with RPA and without Sld2). Atomic coordinates have been deposited in the Protein Data Bank (PDB) with the following accession codes: 9RHL (DH-Sld3-MBD), 9RHM (DH-Sld3/7-Cdc45), 9RHJ (dimeric pre-IC), 9RHI (monomeric pre-IC) and PDB 28VY (sCMGE assembled with Sld2 and RPA).

Field-specific reporting

Please select the one below that is the best fit for your research. If you are not sure, read the appropriate sections before making your selection.

☒ Life sciences ☐ Behavioural & social sciences ☐ Ecological, evolutionary & environmental sciences

For a reference copy of the document with all sections, see nature.com/documents/nr-reporting-summary-flat.pdf

Life sciences study design

All studies must disclose on these points even when the disclosure is negative.

Sample size	Negative stain EM experiments were used to determine the nucleotide dependency of CMG assembly and the salt-stability of CMG complexes formed in the absence or presence of nucleotide. Furthermore, negative stain EM experiments were used to test the effect of omitting or phosphorylating firing factors and to test structure-based mutants. To obtain 2D classes of different reaction intermediates, we usually collected 50 - 150 micrographs for each condition and per replicate since these conditions allow for high-quality 2D class averages suitable for quantitative analysis.<br><br>To yield high-resolution structures of Cdc45 recruitment (DH-Sld3-MBD and DH-Sld3/7-Cdc45), 105,652 micrographs were collected in the cryo-EM experiment from 3 grids. To yield high-resolution structures of the pre-Initiation Complex, 59,347 micrographs were collected from 2 grids.<br><br>No statistical methods were used to predetermine sample size.
Data exclusions	Negative stain and cryo-EM micrographs with poor staining, ice contamination or without graphene oxide, respectively, were excluded. Picked particles that did not align to a distinct class in 2D and 3D (cryo-EM only) were excluded from further analysis.
Replication	The different complexes identified in this study were visualised in multiple experiments (both negative stain and cryo-EM). All biochemical experiments were performed in replicates.
Randomization	For 3D reconstructions independent halves of the dataset were used to compute Fourier Shell Correlation.
Blinding	Blinding is not relevant for single particle reconstruction, because reference free models are used.

Reporting for specific materials, systems and methods

We require information from authors about some types of materials, experimental systems and methods used in many studies. Here, indicate whether each material, system or method listed is relevant to your study. If you are not sure if a list item applies to your research, read the appropriate section before selecting a response.

Materials & experimental systems

n/a	Involved in the study	
☒	☐	Antibodies
☐	☒	Eukaryotic cell lines
☒	☐	Palaeontology and archaeology
☒	☐	Animals and other organisms
☒	☐	Human research participants
☒	☐	Clinical data
☒	☐	Dual use research of concern

Methods

n/a	Involved in the study	
☒	☐	ChIP-seq
☒	☐	Flow cytometry
☒	☐	MRI-based neuroimaging

Eukaryotic cell lines

Policy information about cell lines

Cell line source(s)	Sf21 insect cells were obtained from the Crick Cell Services science technology platform.
Authentication	None of the cell lines were authenticated.
Mycoplasma contamination	Sf21 cells were tested negative for mycoplasma contamination.
Commonly misidentified lines (See ICLAC register)	No commonly misidentified cell lines were used in this study.
