# Supplementary material for: Structure of the pre-initiation complex explains CMGE biogenesis
Source: Nature. 2026 Jun 17;655(8125):1330–8. doi: 10.1038/s41586-026-10657-7 (PMC13421386; doi:10.1038/s41586-026-10657-7)
Supplement: Supplementary file 3 — Peer Review file [file 41586_2026_10657_MOESM3_ESM.pdf]

nature portfolio

Peer Review File

Structure of the Pre-Initiation Complex Explains CMG Biogenesis

Corresponding Author: Dr Alessandro Costa

Any redactions in this file are there to maintain patient confidentiality, the confidentiality of unpublished data, or to remove third-party material.

This file contains all reviewer reports in order by version, followed by all author rebuttals in order by version.

Attachments originally included by the reviewers as part of their assessment can be found at the end of this file.

Version 0:

Reviewer comments:

Referee #1

(Remarks to the Author)
This manuscript from the Costa group presents a comprehensive biochemical and structural dissection of the yeast pre-Initiation Complex (pre-IC) involved in CMG helicase assembly during eukaryotic DNA replication initiation. By combining in vitro reconstitution with purified proteins, cryo-EM structural determination and targeted mutagenesis, the authors visualize the ATP-free pre-IC assembly and delineate how kinase-regulated factor recruitment, MCM ring remodelling and ATP-dependent conformational changes lead to firing-factor ejection and CMG maturation.

The cryo-EM reconstruction of the ATP-free pre-IC is a major achievement, representing the first high-resolution structure of an almost complete set of firing factors bound to MCM. This structure provides valuable mechanistic insight into the interactions between firing factors and MCMs. Although many of the key contacts have been described in previous studies, it is still important to see these interactions in the context of the entire pre-IC.

A particularly notable finding is the absence of Sld2 density on the pre-IC map despite its proposed role in GINS recruitment, but the conclusions and parallels drawn to the metazoan system seem a little premature. The absence of Sld2 does not necessarily exclude Sld2 from participating in GINS recruitment because it may be released following pre-IC formation. Sld2 phosphorylation clearly enhances pre-IC formation efficiency but the mechanistic basis for this effect is not elucidated in the present study. The authors propose that Sld2 is not strictly required for sCMGE formation either and instead functions downstream, potentially paralleling the role of RECQL4 in metazoans but the precise downstream function remains unresolved. The essential nature of Sld2 could arise from inhibiting pre-IC formation and dCMGE splitting additively.

Major points:

1. The colour choices (and colour changes, e.g. in Fig. 1d) for the proteins make it hard to distinguish the various factors in some of the figures. Sld7 and Mcm3 are very similar, as are Mcm2 and Sld3, and Pol e and Mcm4.

2. The authors used the Sld2-8D phosphomimetic variant for in vitro reconstitution and cryo-EM determination, whereas reference (13) employed the Sld2-11D variant, which can support CDK bypass. What was the rationale for selecting Sld2-8D for reconstruction? Could this choice contribute to the absence of Sld2 density in the pre-IC structure? Related to that, DNA replication is reduced in the system used by the authors, suggesting some DDK/CDK functions are not recapitulated.

3. The discussion and presentation of Fig. 2a-d and Ext. Data Fig. 5a,b could be shortened because they are based on previously published results. It should also be clearly justified why experimentally determined structures are reproduced as AF models instead of showing experimental structures.

4. In the pre-IC structure, the authors propose that Sld3 binds both MCM hexamers, but the linker region between the MBD and helical domain seems flexible. Why could both domains not bind the same MCM hexamer?

5. In Fig. 3, the authors show that the homodimerization domain of Sld7 is not required for pre-IC assembly. Is it possible that homodimerization of Sld7 becomes more important when Sld7 is limiting? This could be tested experimentally. Related, is the Sld7 homodimerization domain essential for yeast viability?

6. In the same figure (Fig. 3), the authors measure pre-IC assembly for the Sld7 ΔDD mutant but dCMGE assembly for the Sld7 dropout. This is odd and the same readout should be used for both conditions.

7. Previous studies in Xenopus egg extracts have suggested that two Cdc45 molecules are recruited to origins simultaneously. In Ext. Data Fig. 7f,h, some 2D classes contain only one Cdc45 and the DH–Sld3/7–Cdc45 cryo-EM structure shows two copies of Sld3/7 but only one copy of Cdc45. Do the authors think Cdc45 and Sld3/7 recruitment each occur sequentially for both copies, and sequentially to each other?

8. Dbp11 is known as a phosphoreader of Sld2 and Sld3. Do the authors see any interactions between Dbp11 and Sld3 or between Dbp11 and phospho residues in the pre-IC structure? If not, do the authors think Dbp11 is not a phosphoreader or that there are other states they are missing?

9. The Dbp11 E3 mutant shows robust decrease in pre-IC assembly (Fig. 4d) but no clear defect in DNA replication in vitro (Ext. Data Fig. 7c). This discrepancy deserves an explanation.

10. Since the DH–Sld3/7–Cdc45 structure is asymmetric with only one Cdc45 bound, are there any differences between both hexamers, e.g. in the ATPase sites or pore loops?

11. A major concern relates to the role of Sld2 in pre-IC formation:

i. Given the functional importance of Sld2, the data in Fig. 7 should be reproduced at least in biological duplicates. Currently it appears they are based on a single experiment.

ii. The pre-IC structure was solved from reactions containing Sld2-8D. In the absence of Sld2, how can the authors ensure that the resulting pre-IC assembly is structurally identical to the complex formed with Sld2-8D? In Fig. 7e, the 2D classes of dCMGE in the absence of Sld2 also appear to be asymmetric. Could the authors determine a cryo-EM structure of a pre-IC assembled without Sld2? The reduced pre-IC formation efficiency observed in the absence of Sld2 could cause altered interactions during pre-IC assembly and explain the DNA replication defects.

iii. In Fig. 7b, pre-IC formation efficiency is ~20% for both WT-Sld2 and no-Sld2 reactions. Surprisingly for downstream dCMGE formation, the efficiency is approximately two-fold higher without Sld2 compared to with WT-Sld2. Do the authors think WT-Sld2 is dominant negative in this reaction and what would be the mechanism? Moreover, since the Sld-8D mutant may not perfectly mimic phospho-Sld2, this experiment should be repeated with wt Sld2 -/+ CDK, or better pre-phosphorylated Sld2 as the authors did for Sld3.

Minor points:

1. In the first Results section, the authors state: “This observation agrees with earlier Western blot evidence that high-salt resistant association of GINS and Cdc45 to MCM requires ATP binding.” However, according to Ext. Data Fig. 2e, the pre-IC appears to disassemble into sCMG, in which Cdc45 and GINS remain bound to MCM. This observation does not seem to support the stated conclusion.

2. In Ext. Data Fig. 6d, the last 2D class image from the +Rad53 condition does not resemble an MCM-DH particle. It appears too fuzzy and featureless to be confidently assigned.

3. In Ext. Data Fig. 2b, it is unclear at which step ATP is removed or added. This is not explicitly indicated in panel 2a. 2D class averages from reactions performed without ATP should be included for direct comparison with the ATP-containing condition.

4. In Ext. Data Fig. 5, it would make the data more accessible to non-structural biologists if the authors could highlight the regions in the PAE plots that correspond to the interaction interfaces shown on the left.

5. In Fig. 3b, Sld7 ΔDD is labelled as a deletion of residues 180-258, whereas in the main text it is described as 182-257.

6. In Fig. 3b, Mcm2 is labelled as Mcm4.

7. In the Fig. 5 legend, “Mcm2” is written as “Mc2.”

8. References are missing after the first sentence of the second results section (for DDK docking and Mcm4).

9. What are the blue arrows in Ext. Data Fig. 7f?

10. All abbreviations should be defined the first time they are used.

11. It is hard to keep track of all the buffers used. The authors should provide a summary table for those.

Data reproducibility:

Repeat experiments are not indicated for Figs 1b, 2g, 4h.

Referee #2

(Remarks to the Author)
Multiple initiation factors convert MCM2-7 double hexamers to active Cdc45-MCM-GINS (CMG) helicases during the initiation of DNA replication. This involves the recruitment of Cdc45 and GINS, coupled to a switch of the MCM2-7 heterohexamers from surrounding double-strand DNA at origins to encircling single-strand DNA at forks. The mechanism of CMG assembly and activation is understood poorly, and here the authors use a clever in vitro approach to isolate and determine the structure of a 'pre-initiation complex' (pre-IC) on DNA, using budding yeast proteins that mediate DNA replication initiation. The pre-IC structure helps to explain how Sld3 recruits Cdc45 to MCM2-7 double hexamers, binding Mcm4 of one Mcm2-7 hexamer and recruiting Cdc45 to the other. Moreover, the two MCM2-7 rings of the original double-hexamer are partially separated in the pre-IC, allowing for engagement of MCM2-7 with GINS that is also bound by Dpb11 and the Dpb2 subunit of polymerase epsilon. Interestingly, the opening of the two MCM2-7 rings appears to be stabilised by the previously unanticipated binding of Dpb11 to MCM2-7.

Past work showed that the Sld2 protein is essential for the initiation of DNA replication in budding yeast and is important for CMG assembly. Sld2 is not observed in the pre-IC structure, and the authors show that CMG assembly is not completely blocked in the absence of Sld2. This leads them to suggest that Sld2 also plays a role in the subsequent CMG activation step that requires Mcm10, consistent with recent studies of the Sld2 orthologue RECQL4 in Xenopus laevis. The authors show that single CMG complexes can be produced at a low level from MCM2-7 double hexamers in the absence of Sld2, but the significance is unclear, as the authors do not address whether such single CMG complexes still encircle double-strand DNA (indicating a role for Sld2 in DNA strand exclusion from nascent CMG), or else represent active CMG helicases around single-strand DNA.

Overall, the isolation of the pre-IC and the description of its structure represent a major advance for the field and merit publication in Nature, after the resolution of some specific issues that are listed below. The Sld2 data are also interesting but are rather preliminary and in danger of over-interpretation, as discussed below. Further work would be required before firm conclusions could be made about the putative role of Sld2 in CMG activation and "the essential role of Sld2 during origin activation".

Major points

1. The authors show that the pre-IC can form with reduced efficiency in the absence of Sld2 (Fig. 7). In places, the quantification of the data is hard to understand.

For example, the authors that state that "While ppSld3/7, GINS and Cdc45 were all required for pre-IC formation, dropping out the Sld2 8D phosphomimetic variant still yielded recognisable pre-IC averages, although with 75% reduction in DH-to-preIC conversion efficiency. Similarly reduced efficiency was obtained when swapping Sld2 8D for non-phosphorylated, wild type Sld2 (Figure 7a,b). This indicates that Sld2 phosphorylation by CDK increases pre-IC-assembly efficiency. When repeating the Sld2-dropout experiment in the presence of ATP, dCMGE could be assembled, though DH-to-dCMGE conversion efficiency dropped by 50% (Figure 7b)."

Which figure shows this 75% reduction in DH-to-preIC conversion efficiency? If this is meant to be shown in Fig. 7b (where the -Sld2 sample has a 'CMG assembly' value of about 25%), then what is this in relation to (Sld2-8D has a value of 50%)? In Fig. 7b, it's hard to see how the -Sld2 sample has 'dropped by 50%' (in relation to what?). In Fig. 7b, why does the -Sld2 sample support more CMG assembly than the 'Sld2-Wt' sample?

2. The observation of a low level of CMG assembly in the absence of Sld2 is interesting, but it leads to a discussion that I found confusing and in places misleading, regarding how GINS is recruited to MCM2-7 double hexamers during initiation in budding yeast.

The authors claim that "In yeast, the GINS delivery function had been tentatively assigned to Sld2, based on genetic and biochemical data13,23,25,31". Previous studies showed that each of Sld2, Pol epsilon and Dpb11 are important for GINS recruitment in budding yeast cells. Pol epsilon (Dpb2) and Dpb11 were shown to bind GINS directly, and the original report of a 'pre-Loading Complex' by Araki's lab argued that phospho-Sld2 bridged Pol epsilon to Dpb11, thereby helping Dpb11 and Pol epsilon recruit GINS to MCM2-7 double hexamers.

In the abstract, the authors state that "Different from the textbook view, the essential firing factor Sld2 is not required for delivering GINS to MCM". Later they discuss how GINS recruitment in metazoa requires the DONSON protein, not present in yeast, and they suggest that Dpb11 (but not Sld2) plays the "GINS recruitment function of DONSON". This will be quite confusing for many readers, when the data indicate that both Sld2 and Pol epsilon are important for pre-IC assembly (GINS recruitment), though they are not 100% essential (as a low level of complex assembly is seen in their absence). Residual CMG assembly without Sld2 or Pol epsilon is consistent with past data – the data in the 2015 Nature paper by Yeeles et al. showed that a low level of GINS could be recruited to loaded MCM2-7-Cdc45 in the absence of Sld2 or Pol epsilon (Fig. 2a-

b of that paper), though at the time the emphasis was placed on the importance of these factors (rather than on the fact that they might not be 100% essential).

In the present manuscript, the authors are more careful in some places about discussing the role of Sld2 in pre-IC assembly (e.g. "phospho-Sld2 promotes but is not strictly required for pre-IC assembly"), but it's important to discuss this point consistently throughout the text, without using exaggerated language that risks confusion (including the implication that these data re-write the textbook regarding the role of Sld2).

3. Related to the above point, the authors conclude that since Sld2 'is not required' for CMG assembly yet is essential for DNA replication initiation, the 'essential role' of Sld2 must be downstream of CMG assembly. This is confusing, as Sld2 is important (though not 100% essential) for CMG assembly and so this is likely to be part of the explanation for why Sld2 is essential for replication initiation. Sld2 might also have a role in CMG activation, which could also contribute to its importance in DNA replication initiation, but the 'essentiality' of Sld2 might then be the sum of its two roles (its important contribution to CMG assembly plus its putative role in CMG activation). It doesn't make sense to argue that only the proposed role for Sld2 during CMG activation makes the protein essential for DNA replication initiation. More careful discussion of this point is important to avoid confusion.

4. In addition to finding that Sld2 is not 100% essential for CMG assembly, the authors report that splitting of double-CMGs into two single CMGs (a step dependent on Mcm10) can occur in the absence of Sld2, though with reduced efficiency. This leads them to "conclude that the essential role of Sld2 during origin activation is enacted neither during dCMGE formation nor Mcm10-dependent splitting into two single CMGEs. This changes our understanding of Sld2."

As noted above, 'the essential role of Sld2' during replication initiation might involve the sum of several individual roles, without Sld2 needing to be 100% essential for any one of these. Alternatively, Sld2 might be essential for the transition of CMG from dsDNA to ssDNA (in addition to its contribution to earlier steps such as CMG assembly)?". In their 2024 paper by Henrikus et al. (Nat. Struct. Mol. Biol.), the authors developed a strategy to distinguish single-stranded and double-stranded DNA-interacting particles by cryoEM. Presumably they could use a similar approach to determine whether single CMG complexes that form in the absence of Sld2 (but in the presence of Mcm10) encircle single-strand DNA (as is the case in the presence of Sld2), or still encircle double-strand DNA (identifying a role for Sld2 in the strand exclusion step). The latter possibility would certainly change our understanding of Sld2.

5. In the section entitled "What disrupts the DH interface?", the authors consider several possibilities, such as binding of Sld3 and Dpb11 to Mcm2-7 double hexamer, and release of ADP during pre-IC assembly. The authors suggest that "This Sld3 interaction may contribute to dislodging Mcm7 from its position across the two hexamers, where it protected the Mcm5 A domain in trans.", whereas for Dpb11 they state "From the pre-IC structure alone, it is unclear whether Dpb11 binding across the two MCM hexamers breaks the DH interface, or rather Dpb11 accesses a pre-formed gap within the Nterminal dimerisation interface of MCM."

It is unclear why Sld3 binding is favoured over Dpb11 binding as a trigger for disrupting the DH interface. In both cases it's a 'chicken-and-egg' problem and it's not really possible to say whether binding of either factor is a trigger for disrupting the original MCM2-7 double hexamer, or whether binding of Sld3 and Dpb11 helps to stabilise an open state as in the pre-IC, but something else provides the initial trigger for opening of the double-hexamer interface.

The authors favour ADP release during pre-IC formation as playing an important role: "Disrupting DH dimerisation upon pre-IC formation involves a substantial structural transition, with a change in buried area from 6,816 to 722 Å2. What might provide the energy for this structural change? ADP release upon pre-IC formation (Extended Data Figure 10b) likely plays a role in this process." However, it's currently still unclear how the process is initiated; we simply have several lines of evidence for factors that might contribute (Sld3 binding, Dpb11 binding, ADP release), without any idea about the order of events.

Minor points:

1. Page numbers would have been extremely useful!

2. In several of the figures, starting with Fig. 1c, the authors use 'Apo' to refer to '-ATP' in their reaction schemes. I appreciate the usage of 'Apo' when referring to the structure of a protein in an unbound state, but I suspect that the usage here of 'Apo' in reaction schemes will confuse readers who are not structural biologists, and it would be simpler to just use '-ATP'.

3. "Our results agree with previous biochemical data, which identified the same Sld3 region as essential for MCM binding. In addition to its interaction with Mcm4, Sld3 has also been observed to bind phosphorylated Mcm6 independently (not observed in our structure), indicating a distinct phosphoreader role that complements its function in recognizing DDK-mediated MCM modification (30)."
Deegan et al. 2016 (reference 30) also showed binding of Sld3 to phospho-Mcm4, as well as phospho-Mcm6. Perhaps the authors could refer to this?

4. "What disrupts the DH interface" This needs a question mark!

5. In Ext. Data 5c, the authors use AlphaFold3 to predict that Rad53 phosphorylation of three specific sites in Sld3 should block interaction with Mcm4. Subsequently, they confirm that Rad53 phosphorylation of Sld3 blocks CMG assembly. It would

be good to test the AlphaFold model more specifically, by mutating the three predicted phosphorylation sites in Sld3 and testing whether pre-phosphorylation of mutated Sld3 by Rad53 can no longer inhibit CMG assembly.

6. The authors show that the separation between the two Mcm2-7 rings in the pre-IC is not triggered by Cdc45 recruitment and they conclude that it must “occur in a step that follows Cdc45 recruitment”. Strictly speaking, they have only shown that the separation is not dependent on Cdc45 recruitment, and they have not addressed the timing per se. It’s possible that the separation between the two Mcm2-7 rings occurs at the same time as Cdc45 recruitment but is triggered by something else that happens at the same time. Measuring dependency is not the same as measuring timing.

7. The quantification in Fig. 7b and 7d lacks error bars; was this experiment done more than once?

8. “In Sld2-drop-out conditions, splitting of double-MCM containing complexes (either DHs or dCMGEs) to single CMGEs dropped by 3-fold (from 12 to 4%, Figure 7d,e)”. Fig. 7d seems to show dCMGE splitting, not DH splitting as well.

9. Shouldn’t the reaction scheme in Fig. 7c mention CMG assembly as well as dCMGE splitting?

Referee #3

(Remarks to the Author)
This study by Puhringer et al. uses an elegant in vitro strategy to assemble yeast pre-initiation complexes on origin DNA without CDK and DDK kinases. Authors use cryo-Electron Microscopy to capture the structure of the yeast pre-initiation complex consisting of two CMG helicases bound to Dpb11, Sld3, and Dpb2. The most interesting and novel aspects of this structure is the captured interface between CMG and Sld3, plus the interface between CMG and Dpb11.

However, the structure presented lacks details in most of the Sld3-Cdc45 interacting region, with the exception of an alpha-helix. To compensate for this, the authors refer to a recent crystal structure of Sld3-Cdc45 from the Araki & Yao labs, which reveals the Sld3-Cdc45 interaction in more detail. Unfortunately, the structure presented in this manuscript is also missing the Sld3-Sld7 interacting region, which the authors instead model using AlphaFold.

The presented structure also shows the interaction between a portion of Dpb11 and CMG, and the authors remark that it is similar to how DONSON interacts with CMG in a recent structure of the frog pre-initiation complex. This finding has interesting evolutionary implications about conservation of function rather than conservation of sequence in initiation proteins.

Finally, the authors report that Sld2 is not required to deliver GINS to CMG, and state that Sld2 is likely involved in a step downstream of CMG assembly. However, the conclusion that Sld2 is not strictly required is not entirely novel, as a seminal study from the Diffley lab already showed that CMGs can be assembled inefficiently without Sld2 (Yeeles et al., 2015, Nature, Fig. 2a-b). Beyond speculation, the authors do not provide any direct evidence of how Sld2 acts downstream of CMG assembly.

Overall, this study presents a detailed structural corroboration of several previously-published findings. Although this type of validation is very valuable for the field, the new structure does not present critical new insights. The manuscript is highly technical and very difficult to read for a general audience reader who is not a professional structural biologist. A specialized structure-centric journal may be more appropriate for this work.

Detailed remarks:

1. The Sld3-CDC45 interaction data presented in this structure corroborates the findings from a recently published crystal structure and is not novel on its own.

2. This structure does not show the Sld3-Sld7 interaction interface; the authors use AF3 modeling to support the idea that this heterodimerization should exist in the context of the pre-IC, but it highlights the limitation of the current structure.

3. The manuscript goes into a lot of detail about the yeast pre-IC. This will be of interest to a small subset of readers. Of more interest is what this structure can tell us about the human initiation mechanism. The authors do not comment on such implications.

4. The Dpb11-GINS interaction is not novel (was previously shown in biochemical studies), and was previously shown for xenopus TopBP1-GINS (ref 46).

5. It is unclear what the authors mean here: "Improved DH decoration was obtained upon glutaraldehyde crosslinking followed by nanobody-affinity purification using an ALFA tag47 (Ext. Data Fig. 7g,h, a second, crosslinking-tolerant handle fused to the MH DNA roadblock). The development of this method will be further described in Pühringer et al. in preparation." The authors vaguely describe a technique and then cite an upcoming manuscript “in preparation”. More detail/clarification should be provided, or this statement should be taken out.

6. Fig. 5a,b: Where is the Sld3-Sld7 density? Are the highlighted regions portions of MCM2-7 that interact with Sld3? Or are the highlighted regions portions of Sld3 that are resolved in the structure? The figure captions do not provide clarity on this matter.

7. The authors make the following statement: "We conclude that the separation between the two MCM rings must occur in a step that follows Cdc45 recruitment." This is not a novel conclusion. This is an obvious implication of the recent structure of frog DONSON+CMG (Cvetcovic et al., 2023).

8. The following section about how assembly factors may be ejected is speculative. It should be moved from the results to the discussion section. "In fact, Cdc45 engagement by C-terminal Pol2 sterically clashes with the Sld3 CBD, providing a mechanism for Sld3 ejection (Figure 6g). The ability of two ATP-CMGEs to rotate with respect to one another36 provides another means for the release of CMG assembly factors. Indeed, as soon as two CMGs visit a cis dCMGE configuration, Dpb11 would be dislodged from its position, nestled between Mcm3 and Mcm7 across the two hexamers, (Figure 4a). CMG rotation would also lead to eviction of Sld3 itself,".

9. The authors make the following statement: "So far, we have established that phospho-Sld2 promotes but is not strictly required for pre-IC assembly and stable topological DNA binding through ATP-dependent CMGE conversion." However, the authors do not show how or why Sld2 promotes CMG assembly.

10. The authors state that "We conclude that the essential function of Sld2 during origin activation must be performed downstream of CMGE assembly." This is a speculation rather than a conclusion.

11. In reference to this statement from the manuscript: “In Sld2-drop-out conditions, splitting of double-MCM containing complexes (either DHs or dCMGEs) to single CMGEs dropped by 3-fold (from 12 to 4%, Figure 7d,e). We conclude that the essential role of Sld2 during origin activation is enacted neither during dCMGE formation nor Mcm10-dependent splitting into two single CMGEs. This changes our understanding of Sld2.” Again, the data suggests that Sld2 is important for CMG splitting, but doesn’t indicate that it’s essential for this step. Calling this a “conclusion” is too strong given the data.

12. In this sentence: “This domain is essential for origin activation but functions downstream of, not during, CMG assembly 63-67.”, do the authors mean to say “protein” instead of “domain”?

13. Authors cite ref 67 as supporting the idea that RecQL4 acts downstream of CMG assembly; however, ref 67 reports otherwise, as indicated in its title: Im, J. S. et al. Assembly of the Cdc45-Mcm2-7-GINS complex in human cells requires the Ctf4/And-1, RecQL4, and Mcm10 proteins. Proc Natl Acad Sci USA 106:15628-15632.

14. Regarding this statement “Thus, a more appropriate question is: has the role of SLD2/RECQL4 been repurposed in yeast to make up for the loss of DONSON?” The use of “Sld2/RECQL4” is confusing when referring to yeast, because in yeast the protein is called Sld2.

Referee #4

(Remarks to the Author)
I co-reviewed this manuscript with one of the reviewers who provided the listed reports.

Version 1:

Reviewer comments:

Referee #1

(Remarks to the Author)
The authors have addressed all previous concerns in this revision. The new structure of sCMGE formed without Sld2 that shows duplex DNA engagement over ssDNA engagement is particularly important and strengthens the authors' conclusions about Sld2 function. Congratulations to the authors for this remarkable achievement.

Referee #2

(Remarks to the Author)
The authors have done a good job of addressing the points raised previously. In particular, the revised manuscript contains exciting new data that indicate that Sld2 is required for ejection of one of the two DNA strands from the Mcm2-7 ring of the nascent CMG helicase. I think that the manuscript should be published in Nature, once a couple of remaining issues are clarified – addressing these issues should not take long and will only require textual changes and clarification of the presented data, rather than new experiments.

Specific points

1. Quantification of new data showing that CMGE encircles dsDNA in absence of Sld2

In Fig. 7i the authors present cryoEM structures of single CMGE complexes that were formed in the presence or absence of Sld2, to determine whether Mcm2-7 encircles dsDNA or ssDNA. The figure shows that nascent CMG still encircles dsDNA in the absence of Sld2 (contrasting with ssDNA in the presence of Sld2), which indicates that Sld2 is required for DNA-strand exclusion from the Mcm2-7 central channel. This is an important discovery but it’s not possible from the presented

data to assess this result quantitatively. In other words, did the cryoEM dataset show that single CMGE are exclusively seen around dsDNA, when reactions were performed in the absence of Sld2 (indicating that Sld2 is essential under these conditions for DNA-strand exclusion during initiation, despite the presence of Mcm10), or did some proportion of single CMGE complexes encircle ssDNA and the remainder surround dsDNA (suggesting that both Mcm10 and Sld2 can contribute to DNA strand exclusion)? The authors should clarify this point and provide whatever quantification is possible for the new cryoEM data. This is especially important when it comes to the authors' strong assertion that the DNA strand exclusion step represents the 'essential role' of Sld2 (see next point).

2. I think that the revised manuscript will still cause some unnecessary confusion about the 'essential role' of Sld2, when the reader compares the authors' data to previous papers. The authors assert that "Sld2 exerts its essential function after CMGE formation". Assuming that Sld2 is indeed essential for DNA-strand exclusion (see above – the authors need to clarify the quantification for this point), that doesn't mean that this is the only reason why Sld2 is essential for initiation in vitro and in cells.

The final paragraph of the manuscript downplays the role of Sld2 in recruiting GINS, instead suggesting that Dpb11 plays the GINS recruitment role of metazoan DONSON. This will lead to some confusion, since previous data indicate that the interactions amongst Pol epsilon, Sld2 and Dpb11 jointly support GINS recruitment (likely via contacts on GINS by both Pol epsilon and GINS, which are reinforced by their association with Sld2). For example, Araki's lab showed in Figure 7 of their 2010 Genes Dev paper by Muramatsu et al. that the temperature sensitive lethality caused by mutations in sld2, dpb11 or pol2 (pol epsilon) can be suppressed by over-expression of GINS, consistent with the importance of Sld2-Dpb11-Pol epsilon for GINS recruitment. Metazoan TOPBP1 contacts GINS (like Dpb11) and it remains to be determined whether TOPBP1 also contacts MCM in the preIC, together with DONSON. Did the authors want to highlight that an equivalent interaction of TOPBP1 with MCM (as for Dpb11) would clash with what we know of DONSON's interaction with MCM (suggesting that yeast Dpb11 is really doing something that metazoan TOPBP1 might not)?

To help the reader to interpret the authors' exciting new data alongside previous studies, perhaps they could consider some minor changes to the text, such as:

- Instead of "Sld2 exerts its essential function after CMGE formation" the authors could say "Sld2 plays an essential role after CMGE formation".

- Instead of "we conclude that the essential function of Sld2 during origin activation must be performed downstream of dCMGE assembly" the authors could say "we conclude that Sld2 plays an essential role during origin activation that is downstream of dCMGE assembly."

- Instead of saying "However, Sld2 is not required for making a stable dCMGE complex. Its essential role is instead enacted after holohelicase assembly." the authors could say "However, Sld2 is not absolutely required for making a stable dCMGE complex. In contrast, it plays an essential role after holohelicase assembly."

3. Given the important new insight into Sld2 function, might a revised title for the manuscript be helpful?

4. It's only a minor point, but I couldn't access Ext. Data Table 2 (the file was a duplicate of Ext. Data Table 1).

Referee #3

(Remarks to the Author)
In the revised manuscript, Puhringer et al. address all the major criticisms that we raised for the original manuscript.

1. Most Important Change: by performing additional experiments, the authors now show convincingly that Sld2 plays an important role in double-CMG splitting during CMG activation, and explain the apparent discrepancies between their own data and the data from the Yelles 2015 paper reporting the first reconstitution of origin firing with purified yeast proteins.

2. It is very interesting that in the absence of SLD2 but in the presence of MCM10, the authors observe a significant fraction of split, single-CMGE complexes. Nature must have a way of preventing these single CMGs from initiating uni-directional replication, and the authors hint at this in the discussion. It is also possible that these single-CMGEs on dsDNA might be susceptible to unloading by SCF-dia2 which in involved in canonical replisome unloading during termination.

3. In the revised manuscript the authors do a better job of describing all the differences/similarities between yeast and meatazoan proteins/mechanisms. This helps with clarity, and with highlighting the implications for metazoa.

4. We appreciate that the structures presented in this manuscript represent a major technical feat, and we agree with the other two reviewers that this work in its revised form merits publication in Nature.

Referee #4

(Remarks to the Author)
I co-reviewed this manuscript with one of the reviewers who provided the listed reports.

Version 2:

Reviewer comments:

Referee #2

(Remarks to the Author)
The authors have now addressed the points that I raised. My only query regards the first point that I raised ("Quantification of new data showing that CMGE encircles dsDNA in absence of Sld2"). I had asked the authors to "clarify this point and provide whatever quantification is possible for the new cryoEM data". In the re-revised manuscript, they do indeed clarify the point with their new wording, explaining that sCMGE were only seen around ssDNA in presence of all firing factors, and were only seen around dsDNA in the absence of Sld2. However, they don't actually provide or explain the quantification that lead to this conclusion.

Open Access This Peer Review File is licensed under a Creative Commons Attribution 4.0 International License, which permits use, sharing, adaptation, distribution and reproduction in any medium or format, as long as you give appropriate credit to the original author(s) and the source, provide a link to the Creative Commons license, and indicate if changes were made.
In cases where reviewers are anonymous, credit should be given to 'Anonymous Referee' and the source.
The images or other third party material in this Peer Review File are included in the article's Creative Commons license, unless indicated otherwise in a credit line to the material. If material is not included in the article's Creative Commons license and your intended use is not permitted by statutory regulation or exceeds the permitted use, you will need to obtain permission directly from the copyright holder.
To view a copy of this license, visit https://creativecommons.org/licenses/by/4.0/

We would like to thank the reviewers for a thorough assessment of our work and for the useful feedback provided.

Referee #1:

This manuscript from the Costa group presents a comprehensive biochemical and structural dissection of the yeast pre-Initiation Complex (pre-IC) involved in CMG helicase assembly during eukaryotic DNA replication initiation. By combining in vitro reconstitution with purified proteins, cryo-EM structural determination and targeted mutagenesis, the authors visualize the ATP-free pre-IC assembly and delineate how kinase-regulated factor recruitment, MCM ring remodelling and ATP-dependent conformational changes lead to firing-factor ejection and CMG maturation.

The cryo-EM reconstruction of the ATP-free pre-IC is a major achievement, representing the first high-resolution structure of an almost complete set of firing factors bound to MCM. This structure provides valuable mechanistic insight into the interactions between firing factors and MCMs. Although many of the key contacts have been described in previous studies, it is still important to see these interactions in the context of the entire pre-IC.

We thank the reviewer for the comments. We are pleased that the reviewer deems the structure of the ATP-free pre-IC "a major achievement", providing "valuable mechanistic insight into the interactions between firing factors and MCMs."

A particularly notable finding is the absence of Sld2 density on the pre-IC map despite its proposed role in GINS recruitment, but the conclusions and parallels drawn to the metazoan system seem a little premature. The absence of Sld2 does not necessarily exclude Sld2 from participating in GINS recruitment because it may be released following pre-IC formation.

We completely agree with this comment. In the results section, we now state "So far, we have shown that Sld2 can stimulate but is not needed for pre-IC assembly or dCMGE formation." We later address the role of Sld2 in activation.

Sld2 phosphorylation clearly enhances pre-IC formation efficiency but the mechanistic basis for this effect is not elucidated in the present study.

This is true. The phospho-Sld2 interaction site on Dpb11 is not resolved in our structure. It is known that Sld2 contacts the BRCT3-4 domains of Dpb11 via the essential phospho-Thr84, alongside other ancillary phospho-dependent contacts. However, BRCT3-4 are only flexibly tethered to Dpb11 BRCT1-2, which we were able to observe in our structure. To acknowledge this we now state in the results: "In the pre-IC cryo-EM map, density for all factors previously implicated in CMG assembly can be identified, except for one, Sld2. This was not surprising, given that phospho-Sld2 is known to engage a flexibly tethered region of Dpb11[1], which is not resolved in our pre-IC structure."

The authors propose that Sld2 is not strictly required for sCMGE formation either and instead functions downstream, potentially paralleling the role of RECQL4 in metazoans but the precise downstream function remains unresolved. The essential nature of Sld2 could arise from inhibiting pre-IC formation and dCMGE splitting additively.

We showed in our first submission that pre-IC formation and dCMGE splitting can still occur in the absence of Sld2, though with reduced efficiency. Do these defects, combined, block origin activation by reducing the number of split sCMGEs below a critical threshold required for replication initiation? Or do sCMGEs assembled without Sld2 inherently fail to support origin activation? Below, we describe how we have now addressed this dichotomy.

Major points:

1. The colour choices (and colour changes, e.g. in Fig. 1d) for the proteins make it hard to distinguish the various factors in some of the figures. Sld7 and Mcm3 are very similar, as are Mcm2 and Sld3, and Pol e and Mcm4.

A reconstituted nucleoprotein assembly featuring 38 polypeptides, of which 32 are visible, makes selecting colours a difficult task. We realised we have done a mistake in our first submission. We originally displayed the cryo-EM density for the complete pre-IC view in Figure 1b as semi-transparent, but this was unnecessary. Our original choice made the colours appear different from the opaque representations used elsewhere, creating inconsistency across panels. We have now revised all figures in the manuscript to ensure that colours are consistent throughout. Critically, the colour code used for MCM subunits in the cartoons of Figure 7j is now consistent with the other figures. We feel these changes make figures more intelligible.

2. The authors used the Sld2-8D phosphomimetic variant for in vitro reconstitution and cryo-EM determination, whereas reference (13) employed the Sld2-11D variant, which can support CDK bypass. What was the rationale for selecting Sld2-8D for reconstruction? Could this choice contribute to the absence of Sld2 density in the pre-IC structure? Related to that, DNA replication is reduced in the system used by the authors, suggesting some DDK/CDK functions are not recapitulated.

There are historical reasons why we chose to work with Sld2-8D (bearing aspartate substitutions in T84, S100, S128, S138, T168, S172, S188, S208) rather than the complete 11D variant reported by Tanaka and colleagues. A pilot experiment with the 8D variant yielded informative results that we decided to follow-up on. In fact, we found that this construct supports replication initiation without CDK and pre-IC formation with sufficient efficiency for structural studies. We found this result noteworthy. Data published in 2006 by Tak et al. already indicate that not all 11 sites need to bear phosphomimetic substitutions to bypass CDK requirement for origin activation. In fact, the authors found that T84 (mutated to aspartate in our construct) is the sole CDK target residue strictly required for cell growth and DNA replication. The reviewer is likely correct when noting that Sld2-11D might further promote replication initiation without CDK and pre-IC assembly (in reactions also containing pre-phosphorylated Sld3). In fact, when using the same reagents in a replication reaction in the presence of CDK, the nascent DNA signal is enhanced. This is probably because Sld2-8D becomes phosphorylated at additional sites, although further phosphorylation of the pre-phosphorylated Sld3 reagent might also contribute to improved replication initiation.

To clarify this point we have added a note to in the legend of Figure 1b, stating:

“CDK-pre-phosphorylated Sld3/7 and the Sld2 8D phospho-mimetic mutant support origin-dependent replication reconstituted in a test tube, albeit at lower level (This experiment was

performed two times). The low levels could be due to either the Sld2-8D phosphomimetic mutations only covering a subset of CDK sites, or to inefficient phosphorylation of Sld3 in the isolated pre-phosphorylation reaction, compared to the complete initiation mix."

3. The discussion and presentation of Fig. 2a-d and Ext. Data Fig. 5a,b could be shortened because they are based on previously published results.

We appreciate the reviewer's suggestion. We find it remarkable that the Mcm4 A domain site vacated upon Mcm4-NTI phosphorylation by DDK is occupied by Sld3 to enable Cdc45 recruitment. In our opinion, panels 2a-e provide a complete molecular view of the Cdc45 recruitment pathway and should be displayed in the main figure as originally done. We hope the reviewer will understand our viewpoint.

It should also be clearly justified why experimentally determined structures are reproduced as AF models instead of showing experimental structures.

As stated in Figure legend 2, experimentally determined structures are shown and PDB entry codes are referenced. We address the point raised by the reviewer by stating in the Results section: *"In our positive control, AF3 recapitulated the phospho-dependent disengagement of the N-terminal Mcm4 from A4 in cis, previously described with cryo-EM[2-4] (**Extended Data Figure 5a**)."*. As clear from this passage, the AF3 predictions of previously published experimental structures are reported in Extended Data Figure 5a and not in the main Figure. It is important to include them because they serve as control.

4. In the pre-IC structure, the authors propose that Sld3 binds both MCM hexamers, but the linker region between the MBD and helical domain seems flexible. Why could both domains not bind the same MCM hexamer?

The path from the Cdc45-interacting domain and the MCM binding domain is much shorter for the inter-hexamer than for the intra-hexamer interaction. Also, Sld7 would get in the way of the Sld3 path if the cis but not if the trans interaction is modelled. That said, the reviewer is right in questioning whether a cis interaction could be physically allowed. We cannot rule out this scenario and now acknowledge the possibility in Figure 3. We state "**(b)** Interaction map of Sld3-Cdc45-Mcm2-Mcm6-Sld7 on one MCM hexamer and Sld3-Mcm4 on the opposing hexamer. Also refer to Extended Data Fig. 3k. While Sld3 is adjacent to Mcm4 on the opposed MCM ring, making C-terminal Sld3-Mcm4 engagement most likely, we cannot rule out that Sld3 could take a longer route wrapping around Mcm2,6 and Sld7 to connect Cdc45-binding and MCM-binding domains within the same MCM hexamer."

5. In Fig. 3, the authors show that the homodimerization domain of Sld7 is not required for pre-IC assembly. Is it possible that homodimerization of Sld7 becomes more important when Sld7 is limiting? This could be tested experimentally.

Thank you for bringing up this idea. Prompted by the reviewer, we tested whether the Sld7 homo-dimerisation domain becomes important when concentrations of Sld3/7 heterodimer are limiting. To do this, we titrated Sld3/7 concentrations from 25 nM (generally used in our replication assay) down to 1.25 nM. We found no difference when comparing wt and a Sld3/7 variant bearing a truncated Sld7 homo-dimerisation domain. We share the result with the reviewer, pasted below.

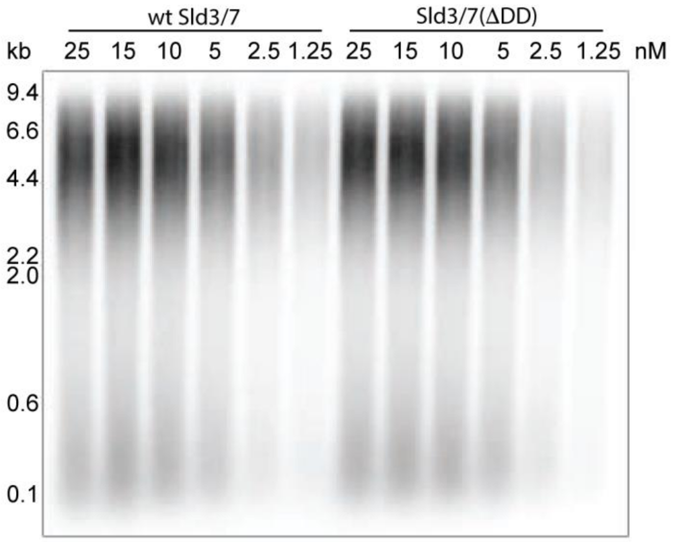

Related, is the Sld7 homodimerization domain essential for yeast viability?

Given that the Sld7 homo-dimerisation domain is not required for efficient replication in vitro and Sld7 itself is not essential for viability in yeast, we believe that performing a yeast viability experiment would not further inform the mechanism of CMG biogenesis. If a defect in viability were to be observed, in fact, we would be unable to explain what else, if not replication initiation, is going wrong in cells when the Sld7 homodimerisation domain is truncated. In summary, we feel that the new reconstituted replication result described above makes the yeast viability experiment requested fall outside the scope of this study.

6. In the same figure (Fig. 3), the authors measure pre-IC assembly for the Sld7 ΔDD mutant but dCMGE assembly for the Sld7 dropout. This is odd and the same readout should be used for both conditions.

To satisfy the reviewer's request we have decided to perform dCMGE assembly reactions in triplicates, for Sld7 ΔDD. This matches the experiment conducted in Sld7 dropout conditions. We find that, while omitting Sld7 altogether negatively affects dCMGE formation efficiency, this is not the case when C-terminal Sld7 is truncated.

7. Previous studies in Xenopus egg extracts have suggested that two Cdc45 molecules are recruited to origins simultaneously. In Ext. Data Fig. 7f,h, some 2D classes contain only one Cdc45 and the DH–Sld3/7–Cdc45 cryo-EM structure shows two copies of Sld3/7 but only one copy of Cdc45. Do the authors think Cdc45 and Sld3/7 recruitment each occur sequentially for both copies, and sequentially to each other?

This is an interesting point. We show that one or two Cdc45 molecules can be recruited to MCM double hexamers, in the absence of GINS. This suggests that Cdc45 recruitment involves two distinct double-hexamer binding events, which might occur before GINS delivery that completes pre-IC and eventually ATP-dCMGE formation. This emerging model agrees with single-molecule analysis of yeast CMG formation from Steve P. Bell's laboratory, described in De Jesus-Kim et al, 2021.While single-molecule work with frog egg

extracts from the Gheorghe Chistol's lab point to a symmetric recruitment of two Cdc45 molecules onto the MCM double hexamer, the homo-dimerisation scaffold is yet to be identified. One possible candidate is TRESLIN/MTBP, the ortholog of yeast Sld3/7. Although TRESLIN/MTBP shares sequence homology with Sld3/7, it varies both in size and domain organisation. This makes synchronous/symmetric Cdc45 recruitment to MCM double hexamers likely to be a metazoan-specific mechanism.

We appended this short paragraph to the Results section entitled "What disrupts the DH interface":

"Our EM imaging of reconstituted Cdc45 recruitment by Sld3/7 reveals one or two Cdc45 molecules engaged to DHs at any one time (**Extended Data Figure 7f,h**), suggesting that Cdc45 is recruited to each MCM hexamer in independent events. This is compatible with single-molecule measurements using yeast proteins[5] and different from single-molecule observations with frog egg extracts. In fact, in the Xenopus system, Cdc45 is recruited to DHs synchronously, via a mechanism that is yet to be uncovered[6]."

8. Dbp11 is known as a phosphoreader of Sld2 and Sld3. Do the authors see any interactions between Dbp11 and Sld3 or between Dbp11 and phospho residues in the pre-IC structure? If not, do the authors think Dbp11 is not a phosphoreader or that there are other states they are missing?

We thank the reviewer for bringing up this question, which prompted us to dig deeper into the Dpb11-Sld3 interaction. With our work we established that Dpb11 interacts with Mcm3 and Mcm7 directly, wedging the MCM double hexamer open via the BRCT domains 1 and 2. Previous structural work from Matthew Day, Laurence Pearl and colleagues on *S. pombe* proteins described the interaction between phospho-Sld3 and the Dpb11 ortholog, Rad4. Specifically, they characterised interactions which are already known to correspond to *S. cerevisiae* Sld3 phosphoresidues Thr600 and Ser622, binding the Dpb11 BRCT1 and BRCT2 domains respectively, and essential for replication initiation. Such interactions can also be recapitulated with AF3 predictions using *S. cerevisiae* sequences. By modelling these phospho-Sld3 contacts onto Dpb11 in our atomic model of the pre-IC, we can now establish that both BRCT sites of Dpb11 are solvent-exposed and hence available to engage phospho-Sld3. The C-terminal end of our atomic model (aa 543), which we had previously built emerging from Mcm7, is perfectly poised to join with phospho-Thr600 engaging Dpb11 according to the Day orthologous structure and AlphaFold. While our map is not of sufficient quality to build these interactions in the atomic structure, we have now included our modelling in **Extended Data Fig. 7e-h.**

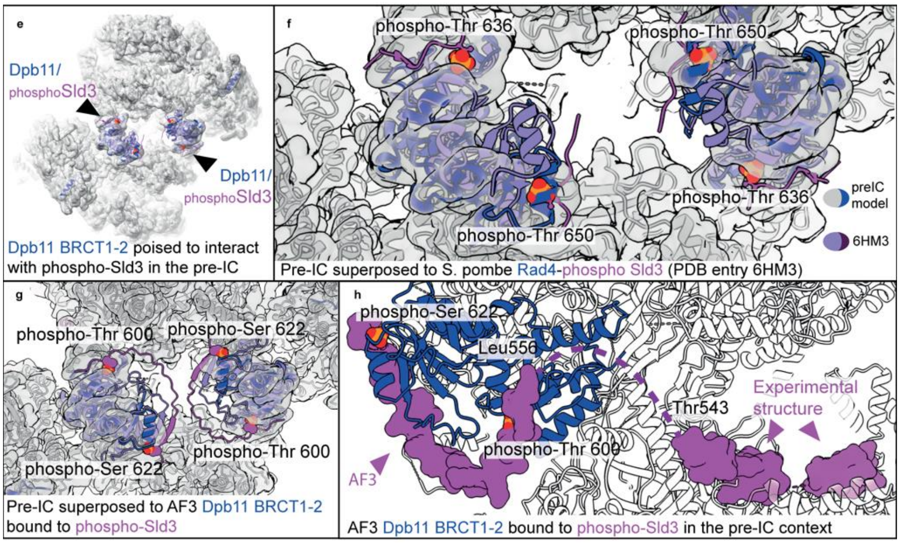

We also added this paragraph to the Results section:

"Dpb11 plays an essential phospho-reader function, however the local resolution of the BRCT1-2 does not allow building phosphorylation-dependent intersubunit contacts. Despite these limitations, superposition of the co-crystal structure of a *S. pombe* orthologs (Rad4 bound to phospho-Sld3[7]) reveals that the phospho-Sld3 binding sites on Dpb11 are solvent-accessible in the pre-IC structure. This result is also recapitulated through AlphaFold modelling showing that *S. cerevisiae* Sld3 phospho-Thr600 and phospho-Ser622, essential for replication initiation, engage the BRCT1 and 2, as previously described with genetics and biochemical analysis[8,9]. When modelled in the full context of the pre-IC experimental structure we note that the C-terminal end of the Sld3 atomic model points towards, and maps close to phospho-Thr600 (64 Å apart, **Extended Data Fig. 7e-h**). This measurement suggests a model whereby Sld3 physically engages Mcm4-6-7 and Dpb11 concurrently.

9. The Dbp11 E3 mutant shows robust decrease in pre-IC assembly (Fig. 4d) but no clear defect in DNA replication in vitro (Ext. Data Fig. 7c). This discrepancy deserves an explanation.

We agree that the DNA replication defect observed for Dpb11-3E is subtle (Extended Data Fig. 7c). In the figure caption of Extended Data Fig. 7c we now state: "A small defect for the variant protein can be detected." To explain the discrepancy noted by the reviewer we continue by stating "The bigger effect observed in pre-IC assembly by EM (Figure 4d) indicates that assessing the formation of this transient structural intermediate is a more sensitive assay compared to DNA replication reconstituted in vitro."

Notably, to corroborate the notion that the 3E mutation does provide an initiation defect as observed in the DNA synthesis product, the combined Dpb11-3E/3X mutation has a bigger effect in the replication assay, compared to the 3X mutation alone.

10. Since the DH–Sld3/7–Cdc45 structure is asymmetric with only one Cdc45 bound, are there any differences between both hexamers, e.g. in the ATPase sites or pore loops?

Because of how the data were processed, through signal-subtraction of residual Cdc45 density from one of the two MCM rings in the double hexamer, we are unable to address this

question.

11. A major concern relates to the role of Sld2 in pre-IC formation:

i. Given the functional importance of Sld2, the data in Fig. 7 should be reproduced at least in biological duplicates. Currently it appears they are based on a single experiment.

We have now repeated the experiment three times with freshly purified proteins. This was applied to pre-IC and dCMGE formation with or without Sld2 wt or the 8D variant. Error bars and P value reported based on ANOVA test. Also repeated three times, we measured the Mcm10-promoted dCMGE splitting with or without Sld2. Error bars and P value reported based on Student's t test. Figure 7 updated accordingly.

ii. The pre-IC structure was solved from reactions containing Sld2-8D. In the absence of Sld2, how can the authors ensure that the resulting pre-IC assembly is structurally identical to the complex formed with Sld2-8D?

Solving a new structure of the pre-IC in the absence of Sld2 would be a major challenge due to the inefficiency of the assembly reaction, and it would likely be indistinguishable from the full complex. In fact, phospho-Sld2 is known to interact with a Dpb11 domain that is invisible in our pre-IC structure. We were nonetheless inspired by this comment from the reviewer and decided to investigate the structural consequence of omitting Sld2 from the activation reaction. In this spirit, we decided to solve a structure assembled will all pre-IC components but Sld2, and split into sCMGE with Mcm10 and in the presence of RPA. The sCMGE protein component of the structure looks identical to that assembled with Sld2 (previously reported in Henrikus et al 2024). What changes is the DNA-engagement state. We find that, with RPA and Sld2, Mcm10-split sCMGE is bound to the fork nexus, and contains single stranded DNA running through the central channel of MCM, as previously reported in several studies. If Sld2 however is dropped out, sCMGE remains duplex-DNA engaged. These new results are now reported in the revised Figure 7j. Together, our data support the notion that pre-IC formation itself is less efficient in the absence of Sld2, but the holohelicase can mature to the step of dCMGE separation into sCMGEs. What fails in the absence of Sld2 is a step that follows CMGE formation, namely lagging-strand ejection from the MCM central channel. This is the step that establishes divergent replication forks.

In Fig. 7e, the 2D classes of dCMGE in the absence of Sld2 also appear to be asymmetric.

Having now performed the experiment in triplicates, we obtained improved dCMGE classes in the absence of Sld2, which we include in the revised Figure 7d. dCMGEs populate both the cis and trans configurations in all conditions tested (wt unphosphorylated Sld2, the Sld2 8D phosphomimetic variant and the Sld2 dropout condition), as previously described for the full reaction with wt Sld2 and CDK.

Could the authors determine a cryo-EM structure of a pre-IC assembled without Sld2? The reduced pre-IC formation efficiency observed in the absence of Sld2 could cause altered interactions during pre-IC assembly and explain the DNA replication defects.

This question is addressed just above in our reply to Point ii.

iii. In Fig. 7b, pre-IC formation efficiency is ~20% for both WT-Sld2 and no-Sld2 reactions. Surprisingly for downstream dCMGE formation, the efficiency is approximately two-fold higher without Sld2 compared to with WT-Sld2. Do the authors think WT-Sld2 is dominant negative in this reaction and what would be the mechanism? Moreover, since the Sld-8D mutant may not perfectly mimic phospho-Sld2, this experiment should be repeated with wt Sld2 -/+ CDK, or better pre-phosphorylated Sld2 as the authors did for Sld3.

Having repeated all experiments described in Figure 7 with freshly purified proteins and in three biological replicates, we can confirm that, in the absence of Sld2, dCMGE formation (which requires ATP) appears relatively more efficient that pre-IC formation (which requires no ATP). We rationalise that this is likely due to the reported enhanced DNA binding stability of these proteins in the presence of ATP (addressed in Douglas et al Nature 2018), which we also show in this study (Extended Data Figure 2c-e). While some variability can be detected using wt or the Sld2 variant, this is not statistically significant. Thus, a -/+CDK experiment would unfortunately not be informative in these experimental conditions. The point of the experiment is to show that dCMGE formation happens efficiently in the absence of Sld2, which is important in the context of our new discovery of sCMGE on duplex DNA in the absence of Sld2. Of note, we have attempted to work with pre-phosphorylated Sld2 but failed to obtain structural-biology grade functional protein.

Minor points:

1. In the first Results section, the authors state: “This observation agrees with earlier Western blot evidence that high-salt resistant association of GINS and Cdc45 to MCM requires ATP binding.” However, according to Ext. Data Fig. 2e, the pre-IC appears to disassemble into sCMG, in which Cdc45 and GINS remain bound to MCM. This observation does not seem to support the stated conclusion.

Thank you for picking up this issue. In the high-salt treated sample we observe single CMG (with no Pol epsilon engaged) in both top as well as bottom-view orientations, as opposed to side-views only. This is generally observed for CMGs that have fallen off duplex DNA (addressed in Lewis et al. 2022). Given what we now know of the pre-IC, earlier observations that GINS and Cdc45 are tightly bound to DNA only if ATP is present should not be interpreted as loose association of GINS/Cdc45 onto DHs, which can be washed off with high salt. Rather, they reflect stable CMG formation in the context of the two-fold symmetric pre-IC assembly, which splits into two single CMGs and falls off DNA upon high-salt wash, when ATP is omitted. For brevity, we decided to revise the sentence mentioned, by putting emphasis on retention by DNA and not by MCM:

“This observation agrees with earlier Western blot evidence that high-salt resistant Cdc45 and GINS binding to licensed-origin DNA requires ATP binding”.

2. In Ext. Data Fig. 6d, the last 2D class image from the +Rad53 condition does not resemble an MCM-DH particle. It appears too fuzzy and featureless to be confidently assigned.

We believe that the 2D average mentioned is a tilted view of a DH. Nonetheless, to address the reviewer’s challenge, we have now re-analysed the dataset and now included four 2D averages of side-view oriented DHs.

3. In Ext. Data Fig. 2b, it is unclear at which step ATP is removed or added. This is not explicitly indicated in panel 2a.

This has now been addressed in Extended Data Fig. 2a, where +/- 1mM ATP is now labelled at the DH biotin elution step.

2D class averages from reactions performed without ATP should be included for direct comparison with the ATP-containing condition.

These classes are included in Figure 1c. We now refer to this in the legend of Extended Data Figure 2b.

4. In Ext. Data Fig. 5, it would make the data more accessible to non-structural biologists if the authors could highlight the regions in the PAE plots that correspond to the interaction interfaces shown on the left.

Done.

5. In Fig. 3b, Sld7 ΔDD is labelled as a deletion of residues 180-258, whereas in the main text it is described as 182-257.

Thank you for spotting this issue. The correct deletion is: 181-257. Updated.

6. In Fig. 3b, Mcm2 is labelled as Mcm4.

Thank you for spotting this. Now corrected.

7. In the Fig. 5 legend, "Mcm2" is written as "Mc2."

Now corrected. Thank you.

8. References are missing after the first sentence of the second results section (for DDK docking and Mcm4).

Thank you. Fixed.

9. What are the blue arrows in Ext. Data Fig. 7f?

We now state that "The blue arrows indicate Cdc45 density."

10. All abbreviations should be defined the first time they are used.

All abbreviations now defined the first time they are used.

11. It is hard to keep track of all the buffers used. The authors should provide a summary table for those.

We have now added a new **Extended Data Table 3** to list all buffers used.

Data reproducibility:

Repeat experiments are not indicated for Figs 1b, 2g, 4h.

All these experiments were done twice. Now mentioned in the Figure legends.

Referee #2:

Multiple initiation factors convert MCM2-7 double hexamers to active Cdc45-MCM-GINS (CMG) helicases during the initiation of DNA replication. This involves the recruitment of Cdc45 and GINS, coupled to a switch of the MCM2-7 heterohexamers from surrounding double-strand DNA at origins to encircling single-strand DNA at forks. The mechanism of CMG assembly and activation is understood poorly, and here the authors use a clever in vitro approach to isolate and determine the structure of a 'pre-initiation complex' (pre-IC) on DNA, using budding yeast proteins that mediate DNA replication initiation. The pre-IC structure helps to explain how Sld3 recruits Cdc45 to MCM2-7 double hexamers, binding Mcm4 of one Mcm2-7 hexamer and recruiting Cdc45 to the other. Moreover, the two MCM2-7 rings of the original double-hexamer are partially separated in the pre-IC, allowing for engagement of MCM2-7 with GINS that is also bound by Dpb11 and the Dpb2 subunit of polymerase epsilon. Interestingly, the opening of the two MCM2-7 rings appears to be stabilised by the previously unanticipated binding of Dpb11 to MCM2-7.

We would like to thank the reviewer for the careful summary and for stating that our approach to isolate the pre-IC in vitro is "clever".

Past work showed that the Sld2 protein is essential for the initiation of DNA replication in budding yeast and is important for CMG assembly. Sld2 is not observed in the pre-IC structure, and the authors show that CMG assembly is not completely blocked in the absence of Sld2. This leads them to suggest that Sld2 also plays a role in the subsequent CMG activation step that requires Mcm10, consistent with recent studies of the Sld2 orthologue RECQL4 in Xenopus laevis. The authors show that single CMG complexes can be produced at a low level from MCM2-7 double hexamers in the absence of Sld2, but the significance is unclear, as the authors do not address whether such single CMG complexes still encircle double-strand DNA (indicating a role for Sld2 in DNA strand exclusion from nascent CMG), or else represent active CMG helicases around single-strand DNA.

The point raised is useful and prompted us to solve a structure of a split sCMGE complex. As described below, we found that sCMGE assembled without Sld2 remain bound to duplex DNA. The conceptual advance derived from our new results represents, in our opinion, a critical addition to our study.

Overall, the isolation of the pre-IC and the description of its structure represent a major advance for the field and merit publication in Nature, after the resolution of some specific issues that are listed below.

We are pleased to read that the reviewer considers the isolation and structure of the pre-IC are "a major advance for the field and merit publication in Nature". Below, we address the issues raised.

The Sld2 data are also interesting but are rather preliminary and in danger of over-interpretation, as discussed below. Further work would be required before firm conclusions could be made about the putative role of Sld2 in CMG activation and "the essential role of Sld2 during origin activation".

We would like to thank the reviewer for raising this issue.

Major points

1. The authors show that the pre-IC can form with reduced efficiency in the absence of Sld2 (Fig. 7). In places, the quantification of the data is hard to understand.

For example, the authors that state that "While ppSld3/7, GINS and Cdc45 were all required for pre-IC formation, dropping out the Sld2 8D phosphomimetic variant still yielded recognisable pre-IC averages, although with 75% reduction in DH-to-preIC conversion efficiency. Similarly reduced efficiency was obtained when swapping Sld2 8D for non-phosphorylated, wild type Sld2 (Figure 7a,b). This indicates that Sld2 phosphorylation by CDK increases pre-IC-assembly efficiency. When repeating the Sld2-dropout experiment in the presence of ATP, dCMGE could be assembled, though DH-to-dCMGE conversion efficiency dropped by 50% (Figure 7b)."

Which figure shows this 75% reduction in DH-to-preIC conversion efficiency? If this is meant to be shown in Fig. 7b (where the -Sld2 sample has a 'CMG assembly' value of about 25%), then what is this in relation to (Sld2-8D has a value of 50%)? In Fig. 7b, it's hard to see how the -Sld2 sample has 'dropped by 50%' (in relation to what?).

We have reworked this whole section being careful to eliminate any ambiguity. Here is the new text:

"While ppSld3/7, GINS and Cdc45 were all required for pre-IC formation, dropping out the Sld2 8D phosphomimetic variant still yielded recognisable pre-IC averages, although DH-to-pre-IC conversion rate dropped by roughly one third (from 28% to 20%, based on 16,318 averageable particles for Sld2 8D, 22,234 for the dropout, each over three biological replicates). Similarly reduced efficiency (based on 20,030 averageable particles) was obtained when swapping Sld2 8D for non-phosphorylated, wild type Sld2 (**Figure 7a-c**). When repeating these experiments in the presence of ATP, dCMGE[10] could be assembled with robust efficiency, reflecting the increased stability provided by nucleotide binding[11] (analysis based on 67,233 averageable particles over the three conditions, **Figure 7d,e**)."

In Fig. 7b, why does the -Sld2 sample support more CMG assembly than the 'Sld2-Wt' sample?

We have now repeated this experiment in triplicates. While variability can be observed, it is not statistically significant.

2. The observation of a low level of CMG assembly in the absence of Sld2 is interesting, but it leads to a discussion that I found confusing and in places misleading, regarding how GINS is recruited to MCM2-7 double hexamers during initiation in budding yeast.

a. The authors claim that "In yeast, the GINS delivery function had been tentatively assigned to Sld2, based on genetic and biochemical data13,23,25,31". Previous studies showed that

each of Sld2, Pol epsilon and Dpb11 are important for GINS recruitment in budding yeast cells. Pol epsilon (Dpb2) and Dpb11 were shown to bind GINS directly, and the original report of a ‘pre-Loading Complex’ by Araki’s lab argued that phospho-Sld2 bridged Pol epsilon to Dpb11, thereby helping Dpb11 and Pol epsilon recruit GINS to MCM2-7 double hexamers.

We have removed the word “tentatively”, for a fairer description of the published work, and are citing the pre-Loading Complex study from the Araki lab. Our own work agrees with data implicating Sld2 in GINS delivery to MCM to form pre-IC, on the path to sCMGE formation.

b. In the abstract, the authors state that “Different from the textbook view, the essential firing factor Sld2 is not required for delivering GINS to MCM”.

We omitted this sentence.

Later they discuss how GINS recruitment in metazoa requires the DONSON protein, not present in yeast, and they suggest that Dpb11 (but not Sld2) plays the “GINS recruitment function of DONSON”. This will be quite confusing for many readers, when the data indicate that both Sld2 and Pol epsilon are important for pre-IC assembly (GINS recruitment), though they are not 100% essential (as a low level of complex assembly is seen in their absence).

To address this fair point from the reviewer, we have revised a key sentence in the discussion as follows: “In agreement with previous work, we find that Sld2 is involved in the efficient delivery of GINS to MCM, as observed in the pre-IC assembly step of CMGE biogenesis. However, Sld2 is not required for making a stable dCMGE complex. Its essential role is instead enacted after holohelicase assembly.”

c. Residual CMG assembly without Sld2 or Pol epsilon is consistent with past data – the data in the 2015 Nature paper by Yeeles et al. showed that a low level of GINS could be recruited to loaded MCM2-7-Cdc45 in the absence of Sld2 or Pol epsilon (Fig. 2a-b of that paper), though at the time the emphasis was placed on the importance of these factors (rather than on the fact that they might not be 100% essential).

The reviewer is correct, thank you for making this point. We now reference to Figure 2a-b:

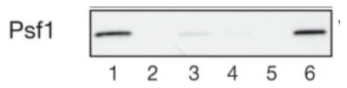

Extracted from Yeeles et al 2015, Figure 2b. This image shows low-level GINS recruitment in the absence of Sld2.

The reviewer will recongise that the amount of GINS (proxy for CMG formation) retained on DNA in the presence of Mcm10 and the absence of Sld2 is visible but significantly reduced compared to conditions where all firing factors are present. We think this is a key point in the activation mechanism, especially in light of our new data, which assign an essential role for Sld2 in the Mcm10-triggered step of dCMGE activation. Here, the dCMGE is split in two sCMGEs, the MCM gate is opened to eject the lagging strand, it is then closed to allow the two activated helicases to cross paths so that they can open two divergent replication forks. Our results imply that these four events are coupled. While we still do not understand the mechanism, we now understand that Sld2 is required for all these events to occur productively.

We address Yeeles et al 2015 Figure 2b in the Discussion section two frame new important questions that arise from our study. We state : "In this respect, we note that, according to Western blot analysis of the same reconstituted system the amount of CMG retained on DNA in the presence of Mcm10 is markedly reduced when Sld2 is omitted. Does this imply that selective strand ejection fails without Sld2, such that not only the lagging but also the leading strand DNA can be evicted, putting CMGEs at risk of falling off the double helix? Is the loss of a sCMGE partner the reason why orphan sCMGEs remain bound to duplex and do not transition to single-stranded DNA? In other words, can lagging-strand ejection only occur when two CMGEs cross paths? From an evolutionary perspective, is the lagging strand ejection fuction of Sld2 conserved in RECQL4[12-15]? Further work is needed to address these topics."

d. In the present manuscript, the authors are more careful in some places about discussing the role of Sld2 in pre-IC assembly (e.g. "phospho-Sld2 promotes but is not strictly required for pre-IC assembly"), but it's important to discuss this point consistently throughout the text, without using exaggerated language that risks confusion (including the implication that these data re-write the textbook regarding the role of Sld2).

We believe the changes detailed in our reply to point 2, above, together address the issue.

3. Related to the above point, the authors conclude that since Sld2 'is not required' for CMG assembly yet is essential for DNA replication initiation, the 'essential role' of Sld2 must be downstream of CMG assembly. This is confusing, as Sld2 is important (though not 100% essential) for CMG assembly and so this is likely to be part of the explanation for why Sld2 is essential for replication initiation. Sld2 might also have a role in CMG activation, which could also contribute to its importance in DNA replication initiation, but the 'essentiality' of Sld2 might then be the sum of its two roles (its important contribution to CMG assembly plus its putative role in CMG activation). It doesn't make sense to argue that only the proposed role for Sld2 during CMG activation makes the protein essential for DNA replication initiation. More careful discussion of this point is important to avoid confusion.

We now better explain that Sld2 is important (though not 100% essential) for GINS delivery to MCM to make pre-IC. In addition, with our revision work we structurally show that split sCMGE assembled at origins in the absence of Sld2 remain bound to duplex and not single-stranded DNA (Figure 7i). This demonstrates that Sld2 is involved in the process of lagging strand ejection.

4. In addition to finding that Sld2 is not 100% essential for CMG assembly, the authors report that splitting of double-CMGs into two single CMGs (a step dependent on Mcm10) can occur in the absence of Sld2, though with reduced efficiency. This leads them to "conclude that the essential role of Sld2 during origin activation is enacted neither during dCMGE formation nor Mcm10-dependent splitting into two single CMGEs. This changes our understanding of Sld2."

As noted above, 'the essential role of Sld2' during replication initiation might involve the sum of several individual roles, without Sld2 needing to be 100% essential for any one of these. Alternatively, Sld2 might be essential for the transition of CMG from dsDNA to ssDNA (in addition to its contribution to earlier steps such as CMG assembly)?". In their 2024 paper by Henrikus et al. (Nat. Struct. Mol. Biol.), the authors developed a strategy to

distinguish single-stranded and double-stranded DNA-interacting particles by cryoEM. Presumably they could use a similar approach to determine whether single CMG complexes that form in the absence of Sld2 (but in the presence of Mcm10) encircle single-strand DNA (as is the case in the presence of Sld2), or still encircle double-strand DNA (identifying a role for Sld2 in the strand exclusion step). The latter possibility would certainly change our understanding of Sld2.

We liked the suggestion from the reviewer and decided to solve the structure of a single CMGE assembled at origins but without Sld2. First, we attempted to assemble sCMGEs using the Henrikus protocol. This involved purifying DDK-free phosphorylated DHs and making sCMGEs by adding all firing factors in solution. The low efficiency of sCMGE formation combined with the high background noise derived from the spent firing rendered structure determination impossible. We hence decided to purify sCMGEs assembled with Mcm10 and RPA, using the two-step methyltransferase pulldown protocol employed elsewhere in our current study. This allowed us to increase MCM-containing particle density while minimising non-DNA bound particles. While in the presence of Sld2 Mcm10-split sCMGEs encircled single-stranded DNA, in its absence they encircled duplex DNA. This establishes that Sld2 has a role downstream of CMGE formation in the process of lagging strand ejection that achieves replication fork establishment. The new structural data are reported in Figure 7j. We agree with the reviewer that, having proven their prediction right, our new structural data change our understanding of Sld2.

5. In the section entitled "What disrupts the DH interface?", the authors consider several possibilities, such as binding of Sld3 and Dpb11 to Mcm2-7 double hexamer, and release of ADP during pre-IC assembly. The authors suggest that "This Sld3 interaction may contribute to dislodging Mcm7 from its position across the two hexamers, where it protected the Mcm5 A domain in trans.", whereas for Dpb11 they state "From the pre-IC structure alone, it is unclear whether Dpb11 binding across the two MCM hexamers breaks the DH interface, or rather Dpb11 accesses a pre-formed gap within the Nterminal dimerisation interface of MCM."

It is unclear why Sld3 binding is favoured over Dpb11 binding as a trigger for disrupting the DH interface. In both cases it's a 'chicken-and-egg' problem and it's not really possible to say whether binding of either factor is a trigger for disrupting the original MCM2-7 double hexamer, or whether binding of Sld3 and Dpb11 helps to stabilise an open state as in the pre-IC, but something else provides the initial trigger for opening of the double-hexamer interface.

Thank you for bringing up this point. We now altered the start of the Results paragraph "What disrupts the DH interface". It now reads: "From the pre-IC structure alone, it is unclear whether Dpb11 binding across the two MCM hexamers breaks the DH interface, or rather Dpb11 accesses a pre-formed gap within the N-terminal dimerisation interface of MCM. Similarly, we cannot tell whether Mcm7 binding by Sld3 causes, or is enabled by, the DH breaking. To address these questions, …" Later in the same paragraph, to acknowledge the role of Mcm7 NTI in stabilising the DH, we added "We solved a 3.1 Å resolution structure of a DH featuring Sld3 density at the A4 site, which possibly represents a first recruitment intermediate. In this structure Mcm7 NTI still tightly interacts with Mcm5, sealing the interface between the two hexamers (**Figure 5a**)."

We feel that, with this rewrite, we do a better job at explaining that indeed Sld3 binding is not sufficient to disrupt the double hexamer interface.

The authors favour ADP release during pre-IC formation as playing an important role: "Disrupting DH dimerisation upon pre-IC formation involves a substantial structural transition, with a change in buried area from 6,816 to 722 Å2. What might provide the energy for this structural change? ADP release upon pre-IC formation (Extended Data Figure 10b) likely plays a role in this process." However, it's currently still unclear how the process is initiated; we simply have several lines of evidence for factors that might contribute (Sld3 binding, Dpb11 binding, ADP release), without any idea about the order of events.

Our intention was to acknowledge small changes that could explain an allosteric reconfiguration, to then focus our attention on the structure of duplex DNA itself, which transitions from a compressed to a relaxed state similar to a spring. We realised, upon reading reviewer's point raised, that we clearly failed to make our point in this passage of the results section. To also incorporate the reviewer's point (see underlined text), we now reworked this paragraph as follows:

"Several changes in the nucleoprotein assembly suggest a mechanism, <u>although the sequence of events remains unclear</u>. For example, comparison with the DH reveals that ADP is released upon pre-IC formation (**Extended Data Figure 10b**), which could promote long-range allosteric changes. Also, MCM binding by GINS-Cdc45 appears to rearrange the N- and C-terminal tiers of MCM[16,17], likely contributing to the disruption of the DH dimerisation interface. The most striking structural change, however, occurs for the double helix, which transitions from a compressed to a relaxed state, similar to a loaded spring snapping free."

Minor points:

1. Page numbers would have been extremely useful!

Now page number added as per reviewer's request.

2. In several of the figures, starting with Fig. 1c, the authors use 'Apo' to refer to '-ATP' in their reaction schemes. I appreciate the usage of 'Apo' when referring to the structure of a protein in an unbound state, but I suspect that the usage here of 'Apo' in reaction schemes will confuse readers who are not structural biologists, and it would be simpler to just use '-ATP'.

We have swapped "Apo" for "No ATP" in Figure 1c, 6b, 6e, 11a and Extended Data Fig. 6f. We have retained Apo, when referring to the structure of an ATPase site, in Extended Data Figure 10. Thank you for the useful remark.

3. "Our results agree with previous biochemical data, which identified the same Sld3 region as essential for MCM binding. In addition to its interaction with Mcm4, Sld3 has also been observed to bind phosphorylated Mcm6 independently (not observed in our structure), indicating a distinct phosphoreader role that complements its function in recognizing DDK-mediated MCM modification (30)."
Deegan et al. 2016 (reference 30) also showed binding of Sld3 to phospho-Mcm4, as well as phospho-Mcm6. Perhaps the authors could refer to this?

We changed the sentence to: “In addition to the interaction with Mcm4 described above, Sld3 has also been observed to bind specific phosphorylated segments of Mcm4 and Mcm6 independently (not observed in our structure). This indicates a distinct phospho-reader role for Sld3, which complements its function in recognizing a DDK-dependent structural change in MCM”.

4. “What disrupts the DH interface” This needs a question mark!

We intend this paragraph title to be a statement, not a question. We believe that omitting the question mark is grammatically correct.

5. In Ext. Data 5c, the authors use AlphaFold3 to predict that Rad53 phosphorylation of three specific sites in Sld3 should block interaction with Mcm4. Subsequently, they confirm that Rad53 phosphorylation of Sld3 blocks CMG assembly. It would be good to test the AlphaFold model more specifically, by mutating the three predicted phosphorylation sites in Sld3 and testing whether pre-phosphorylation of mutated Sld3 by Rad53 can no longer inhibit CMG assembly.

Rad53 phosphorylates multiple sites on Sld3. These modifications likely contribute to abrogating different Sld3 interactions in the pre-IC. Therefore, we think it is unlikely that Rad53-mediated inhibition of CMG assembly will be blocked by introducing alanine mutations in only three Rad53 target residues on Sld3 (T519, S521 and T524), which we could observe engaging Mcm4 in our atomic model. Nonetheless, to satisfy the reviewer’s curiosity, we have confirmed using mass spectrometry that T519, S521 and T524 are indeed modified by Rad53 in the pre-phosphorylated Sld3 reagent used in our nsEM experiment.

6. The authors show that the separation between the two Mcm2-7 rings in the pre-IC is not triggered by Cdc45 recruitment and they conclude that it must “occur in a step that follows Cdc45 recruitment”. Strictly speaking, they have only shown that the separation is not dependent on Cdc45 recruitment, and they have not addressed the timing per se. It’s possible that the separation between the two Mcm2-7 rings occurs at the same time as Cdc45 recruitment but is triggered by something else that happens at the same time. Measuring dependency is not the same as measuring timing.

The reviewer is correct. We modified this sentence by stating: “We conclude that the separation between the two MCM rings must involve Sld2, Dpb11, GINS, Pol epsilon recruitment or a subset of these factors.”

7. The quantification in Fig. 7b and 7d lacks error bars; was this experiment done more than once?

To describe pre-IC assembly, dCMGE assembly and splitting efficiency, we have now repeated the experiment in triplicates with freshly purified proteins, added the error bars in the graphs and included P values in the figure legend to report statistical significance.

8. “In Sld2-drop-out conditions, splitting of double-MCM containing complexes (either DHs or dCMGEs) to single CMGEs dropped by 3-fold (from 12 to 4%, Figure 7d,e)”. Fig. 7d seems to show dCMGE splitting, not DH splitting as well.

We have reworked Figure 7, being careful to eliminate all ambiguity.

9. Shouldn't the reaction scheme in Fig. 7c mention CMG assembly as well as dCMGE splitting?

The cartoon model (now Fig. 7j) has now been modified to incorporate the reviewer's comment and also include the new information on the essential role of Sld2 in lagging-strand ejection after CMGE formation.

Referee #3:

This study by Puhringer et al. uses an elegant in vitro strategy to assemble yeast pre-initiation complexes on origin DNA without CDK and DDK kinases. Authors use cryo-Electron Microscopy to capture the structure of the yeast pre-initiation complex consisting of two CMG helicases bound to Dpb11, Sld3, and Dpb2. The most interesting and novel aspects of this structure is the captured interface between CMG and Sld3, plus the interface between CMG and Dpb11.

We would like to thank the reviewer for stating that our strategy to isolate the yeast pre-initiation complex is elegant and for acknowledging the discovery of new Sld3-MCM and Dpb11-MCM interactions.

However, the structure presented lacks details in most of the Sld3-Cdc45 interacting region, with the exception of an alpha-helix.

To compensate for this, the authors refer to a recent crystal structure of Sld3-Cdc45 from the Araki & Yao labs, which reveals the Sld3-Cdc45 interaction in more detail. Unfortunately, the structure presented in this manuscript is also missing the Sld3-Sld7 interacting region, which the authors instead model using AlphaFold.

The presented structure also shows the interaction between a portion of Dpb11 and CMG, and the authors remark that it is similar to how DONSON interacts with CMG in a recent structure of the frog pre-initiation complex. This finding has interesting evolutionary implications about conservation of function rather than conservation of sequence in initiation proteins.

We would like to thank the reviewer for appreciating the parallel we draw between the function of yeast Dpb11 and metazoan DONSON in GINS recruitment.

Finally, the authors report that Sld2 is not required to deliver GINS to CMG, and state that Sld2 is likely involved in a step downstream of CMG assembly. However, the conclusion that Sld2 is not strictly required is not entirely novel, as a seminal study from the Diffley lab already showed that CMGs can be assembled inefficiently without Sld2 (Yeeles et al., 2015, Nature, Fig. 2a-b).

Please refer to the answer to Reviewer 2, point 2c.

Beyond speculation, the authors do not provide any direct evidence of how Sld2 acts downstream of CMG assembly.

We have now tackled this issue by solving the structure of a split sCMGE assembled in the absence of Sld2. We observe that lagging strand ejection is defective in these conditions, given that MCM remains bound to duplex DNA. In addition, we have now repeated in triplicates the sCMGE formation experiment in the presence or absence of Sld2. We confirm our original observation that dCMGE splitting is inefficient when Sld2 is dropped out and can now state that the difference in sCMGE conversion efficiency is statistically significant. These results are reported in Figure 7.

Overall, this study presents a detailed structural corroboration of several previously-published findings. Although this type of validation is very valuable for the field, the new structure does not present critical new insights.

We respectfully disagree with the reviewer's statement, also because it appears somewhat at odds with their acknowledgement just a few lines above of the mechanistic novelty and evolutionary implications of our findings. Beyond the newly identified interactions, we would like to emphasise that the existence of a pre-initiation complex (where the CMG is assembled by different firing factors as a whole, rather than through a series of independent, possibly sequential interactions) had only been postulated and not biochemically demonstrated in the past. In this study, we not only show that the complex exists and can be isolated (using what the reviewer described as "an elegant in vitro strategy"). We determine its cryo-EM structure and also describe the mechanism whereby pre-IC matures to become dCMGE. Furthermore, with our revision work, we reveal that sCMGEs fail to eject the lagging strand if Sld2 is omitted, establishing an essential helicase activation function for Sld2 that occurs after CMGE assembly. This observation allows us to corroborate a parallel between the function of Sld2 and the metazoan ortholog RecQL4, which we had drawn already in our first submission. For these reasons, we believe our work critically extends previous genetic and biochemical characterisations of the CMG biogenesis pathway.

The manuscript is highly technical and very difficult to read for a general audience reader who is not a professional structural biologist. A specialized structure-centric journal may be more appropriate for this work.

We have revised our manuscript to ensure that readability is improved.

Detailed remarks:

1. The Sld3-CDC45 interaction data presented in this structure corroborates the findings from a recently published crystal structure and is not novel on its own.

In the revised version of our manuscript, we modelled key new features of the Dpb11 interaction with phospho-Sld3, using published work from Matthew Day and Laurence Pearl on a S. pombe Dpb11 ortholog, Rad4. Our findings of new Sld3-MCM interactions, combined with prior knowledge on the Cdc45 and Dpb11 interface, provide a complete view of the role of Sld3 in CMG biogenesis.

2. This structure does not show the Sld3-Sld7 interaction interface; the authors use AF3 modeling to support the idea that this heterodimerization should exist in the context of the pre-IC, but it highlights the limitation of the current structure.

The pre-IC structure does not describe the Sld3-7 interaction interface (because this element is flexibly tethered to the rest of the complex) but it describes an interface between Mcm6 and Sld7, which was previously unknown.

Also, we would like to respectfully clarify that Sld3-Sld7 heterodimerisation is not in question. Indeed, Sld3-7 was purified as a heterodimer, as stated in the methods section. A structure of the Sld3-Sld7 hetero-dimerisation domain was previously determined by Hiro Araki in 2015. In Extended Data Fig. 6k we show the low-pass filtered version of the complete pre-IC where density connecting Sld7 with the Sld3 Cdc45-binding domain can be observed. An AF3 model reported in Figure 6j helps interpret the lower resolution cryo-EM density, in a region that is inherently flexible. In assessing the importance of observing the Sld3-Sld7 interaction interface at high resolution, one should take into account that Sld7 is not essential for viability.

3. The manuscript goes into a lot of detail about the yeast pre-IC. This will be of interest to a small subset of readers. Of more interest is what this structure can tell us about the human initiation mechanism. The authors do not comment on such implications.

Because replication initiation has not yet been reconstituted with human proteins, it is not yet clear if all factors required to start human replication have been identified. Understanding yeast origin activation helps understand the organising principles of replisome assembly. This does allow us to draw parallels with metazoan replication systems. For example, in the discussion we compare yeast Dpb11 and DONSON, which has been lost during evolution in yeast. In the introductory paragraph, the reviewer highlights this comparison as a valuable feature of our paper, which we appreciate. We draw an important parallel between Sld2 and the metazoan ortholog, RECQL4, in the Discussion section, pointing to what is emerging as a post-CMG-assembly initiation fuction conserved from yeast to metazoan.

4. The Dpb11-GINS interaction is not novel (was previously shown in biochemical studies), and was previously shown for xenopus TopBP1-GINS (ref 46).

The reviewer is correct. Our observation that the GINI domain plays the same structural role in yeast and frog was exciting to see, as it perfectly agrees with a model proposed by Hiro Araki, whereby a divergent element exists in both yeast Dpb11 and metazoan TopBP1, which is responsible for GINS recruitment via the same mechanism. This was satisfactory for us to observe.

5. It is unclear what the authors mean here: "Improved DH decoration was obtained upon glutaraldehyde crosslinking followed by nanobody-affinity purification using an ALFA tag47 (Ext. Data Fig. 7g,h, a second, crosslinking-tolerant handle fused to the MH DNA roadblock). The development of this method will be further described in Pühringer et al. in preparation." The authors vaguely describe a technique and then cite an upcoming manuscript “in preparation”. More detail/clarification should be provided, or this statement should be taken out.

The experimental procedure is described in detail in the Methods section, in the paragraph entitled “ALFA pulldown of pre-IC and dCMGE complexes”. We decided to omit the statement “The development of this method will be further described in Pühringer et al. in preparation.” as it is not informative.

6. Fig. 5a,b: Where is the Sld3-Sld7 density?

It is in Extended Data Fig. 6k. We now point to this panel in Figure legend 3b.

Are the highlighted regions portions of MCM2-7 that interact with Sld3? Or are the highlighted regions portions of Sld3 that are resolved in the structure? The figure captions do not provide clarity on this matter.

The colour legend under Figure 5a,b states that MCM is white, Cdc45 is grey, Sld3 is magenta, Sld7 is light blue. This is also marked with arrows. Despite the redundancy we thought that this would help interpret the volume unambiguously. The reviewer will recognise, for example, that the Cdc45 density coloured in grey cannot possibly belong to MCM, or that some of the Sld3 density contacts Cdc45 and not MCM. We hope that the reviewer will agree with us that the figure and the colour legend are intelligible.

7. The authors make the following statement: "We conclude that the separation between the two MCM rings must occur in a step that follows Cdc45 recruitment." This is not a novel conclusion. This is an obvious implication of the recent structure of frog DONSON+CMG (Cvetcovic et al., 2023).

We respectfully disagree with the point raised. We cannot envisage how data contained in the DONSON study, which our lab conducted, address the question of the structural changes promoted upon Cdc45 vs GINS recruitment. The Cvetkovic paper described a double CMG structure engaged by dimeric DONSON, where the two CMGs are partially separated, although they exist in an arrangement distinct from that observed in the yeast pre-IC structure. In the DONSON paper, we did not address whether separation of the two MCM hexamers occurs upon or after Cdc45 recruitment and whether DONSON is involved in this process. Besides these remarks, DONSON has been lost during evolution in yeast, hence little can be inferred regarding yeast origin activation from the double CMG-DONSON structure.

8. The following section about how assembly factors may be ejected is speculative. It should be moved from the results to the discussion section. "In fact, Cdc45 engagement by C-terminal Pol2 sterically clashes with the Sld3 CBD, providing a mechanism for Sld3 ejection (Figure 6g). The ability of two ATP-CMGEs to rotate with respect to one another36 provides another means for the release of CMG assembly factors. Indeed, as soon as two CMGs visit a cis dCMGE configuration, Dpb11 would be dislodged from its position, nestled between Mcm3 and Mcm7 across the two hexamers, (Figure 4a). CMG rotation would also lead to eviction of Sld3 itself,".

We appreciate the suggestion to move the pre-IC disassembly section to the Discussion. However, we believe that this paragraph belongs to the Results section. It describes several structural features of the pre-IC that deserve to be highlighted in Figure 6 and relates to the experiments described in Figure 6e-f pertaining to the disassembly process.

9. The authors make the following statement: "So far, we have established that phospho-Sld2 promotes but is not strictly required for pre-IC assembly and stable topological DNA binding through ATP-dependent CMGE conversion." However, the authors do not show how or why Sld2 promotes CMG assembly.

This is true. We cannot comment on how Sld2 promotes CMG assembly based on our data. This is because we cannot observe Dpb11 BRCT domains 3 and 4 and the majority of Pol

epsilon in our structure. In fact, these two domains are predicted to be flexibly tethered to BRCT1 and 2, which in turn we observe forming the wedge the splits the MCM double hexamer in the pre-IC. It is however known that 11 phosphomimetic amino acid changes (Ser/Thr-to-Asp substitutions) of Sld2, which target CDK sites, support replication initiation. In particular, Thr84 alone is sufficient to support initiation and is known to directly engage Dbp11 BRCT3 and 4.

10. The authors state that "We conclude that the essential function of Sld2 during origin activation must be performed downstream of CMGE assembly." This is a speculation rather than a conclusion.

We have further investigated this issue by determining the cryo-EM structure of a single CMGE originated from a double CMGE when Mcm10 is supplemented. We find that sCMGE in the absence of Sld2 remains bound to duplex DNA, meaning it is defective in the lagging-strand ejection step would establish the replication fork. We feel that this defines the helicase activation role of Sld2, downstream of CMGE assembly.

11. In reference to this statement from the manuscript: “In Sld2-drop-out conditions, splitting of double-MCM containing complexes (either DHs or dCMGEs) to single CMGEs dropped by 3-fold (from 12 to 4%, Figure 7d,e). We conclude that the essential role of Sld2 during origin activation is enacted neither during dCMGE formation nor Mcm10-dependent splitting into two single CMGEs. This changes our understanding of Sld2.” Again, the data suggests that Sld2 is important for CMG splitting, but doesn’t indicate that it’s essential for this step. Calling this a “conclusion” is too strong given the data.

We show that double CMGE splitting into two single CMGEs can occur without Sld2, although with reduced efficiency. The reviewer’s comment prompted us to look into the mechanism of CMGE activation by Sld2 further. To do this, we solved two new cryo-EM structures. Based on the new data we can now state that, in Sld2 dropout conditions, sCMGEs fail to eject the lagging strand and remain bound to duplex DNA instead. This establishes what the essential role of Sld2 is, after CMG assembly.

12. In this sentence: “ is essential for origin activation but functions downstream of, not during, CMG assembly 63-67.”, do the authors mean to say “protein” instead of “domain”?

Apologies for being unclear. We do mean domain - i.e. the Sld2-homology domain. To make the sentence unambiguous we have now made the following change: “This domain alone is essential for origin activation but functions downstream of, not during, CMG assembly”.

13. Authors cite ref 67 as supporting the idea that RecQL4 acts downstream of CMG assembly; however, ref 67 reports otherwise, as indicated in its title: Im, J. S. et al. Assembly of the Cdc45-Mcm2-7-GINS complex in human cells requires the Ctf4/And-1, RecQL4, and Mcm10 proteins. Proc Natl Acad Sci USA 106:15628-15632.

Thank you for spotting a mistake. We have now removed the Im reference as it does not support our statement.

14. Regarding this statement “Thus, a more appropriate question is: has the role of SLD2/RECQL4 been repurposed in yeast to make up for the loss of DONSON?” The use of

“Sld2/RECQL4” is confusing when referring to yeast, because in yeast the protein is called Sld2.

Thank you for bringing up this issue. We changed the sentence to “Thus, a more appropriate question is: has the role of the Sld2 (metazoan RECQL4) been repurposed in yeast to make up for the loss of DONSON?”

References for the debuttal document

1 Tak, Y. S., Tanaka, Y., Endo, S., Kamimura, Y. & Araki, H. A CDK-catalysed regulatory phosphorylation for formation of the DNA replication complex Sld2-Dpb11. *EMBO J* **25**, 1987-1996, doi:10.1038/sj.emboj.7601075 (2006).

2 Saleh, A. *et al.* The structural basis of Cdc7-Dbf4 kinase dependent targeting and phosphorylation of the MCM2-7 double hexamer. *Nature communications* **13**, 2915, doi:10.1038/s41467-022-30576-1 (2022).

3 Cheng, J. *et al.* Structural Insight into the MCM double hexamer activation by Dbf4-Cdc7 kinase. *Nature communications* **13**, 1396, doi:10.1038/s41467-022-29070-5 (2022).

4 Greiwe, J. F. *et al.* Structural mechanism for the selective phosphorylation of DNA-loaded MCM double hexamers by the Dbf4-dependent kinase. *Nature structural & molecular biology* **29**, 10-20, doi:10.1038/s41594-021-00698-z (2022).

5 De Jesus-Kim, L. *et al.* DDK regulates replication initiation by controlling the multiplicity of Cdc45-GINS binding to Mcm2-7. *eLife* **10**, doi:10.7554/eLife.65471 (2021).

6 Terui, R., Berger, S. E., Sambel, L. A., Song, D. & Chistol, G. Single-molecule imaging reveals the mechanism of bidirectional replication initiation in metazoa. *Cell* **187**, 3992-4009 e3925, doi:10.1016/j.cell.2024.05.024 (2024).

7 Day, M. *et al.* BRCT domains of the DNA damage checkpoint proteins TOPBP1/Rad4 display distinct specificities for phosphopeptide ligands. *eLife* **7**, doi:10.7554/eLife.39979 (2018).

8 Zegerman, P. & Diffley, J. F. Phosphorylation of Sld2 and Sld3 by cyclin-dependent kinases promotes DNA replication in budding yeast. *Nature* **445**, 281-285, doi:10.1038/nature05432 (2007).

9 Tanaka, S. *et al.* CDK-dependent phosphorylation of Sld2 and Sld3 initiates DNA replication in budding yeast. *Nature* **445**, 328-332, doi:10.1038/nature05465 (2007).

10 Lewis, J. S. *et al.* Mechanism of replication origin melting nucleated by CMG helicase assembly. *Nature* **606**, 1007-1014, doi:10.1038/s41586-022-04829-4 (2022).

11 Douglas, M. E., Ali, F. A., Costa, A. & Diffley, J. F. X. The mechanism of eukaryotic CMG helicase activation. *Nature* **555**, 265-268, doi:10.1038/nature25787 (2018).

12 Thangavel, S. *et al.* Human RECQ1 and RECQ4 helicases play distinct roles in DNA replication initiation. *Mol Cell Biol* **30**, 1382-1396, doi:10.1128/MCB.01290-09 (2010).

13 Matsuno, K., Kumano, M., Kubota, Y., Hashimoto, Y. & Takisawa, H. The N-terminal noncatalytic region of Xenopus RecQ4 is required for chromatin binding of DNA polymerase alpha in the initiation of DNA replication. *Mol Cell Biol* **26**, 4843-4852, doi:26/13/4843 10.1128/MCB.02267-05 (2006).

14 Sanuki, Y. *et al.* RecQ4 promotes the conversion of the pre-initiation complex at a site-specific origin for DNA unwinding in Xenopus egg extracts. *Cell Cycle* **14**, 1010-1023, doi:10.1080/15384101.2015.1007003 (2015).
15 Kliszczak, M. *et al.* Interaction of RECQ4 and MCM10 is important for efficient DNA replication origin firing in human cells. *Oncotarget* **6**, 40464-40479, doi:10.18632/oncotarget.6342 (2015).
16 Abid Ali, F. *et al.* Cryo-EM structures of the eukaryotic replicative helicase bound to a translocation substrate. *Nature communications* **7**, 10708, doi:10.1038/ncomms10708 (2016).
17 Yuan, Z. *et al.* Structure of the eukaryotic replicative CMG helicase suggests a pumpjack motion for translocation. *Nature structural & molecular biology* **23**, 217-224, doi:10.1038/nsmb.3170 (2016).

Response to Reviewer 2

The authors have done a good job of addressing the points raised previously. In particular, the revised manuscript contains exciting new data that indicate that Sld2 is required for ejection of one of the two DNA strands from the Mcm2-7 ring of the nascent CMG helicase. I think that the manuscript should be published in Nature, once a couple of remaining issues are clarified – addressing these issues should not take long and will only require textual changes and clarification of the presented data, rather than new experiments.

We would like to thank the reviewer for the positive assessment of our revised manuscript.

Specific points

1. Quantification of new data showing that CMGE encircles dsDNA in absence of Sld2

In Fig. 7i the authors present cryoEM structures of single CMGE complexes that were formed in the presence or absence of Sld2, to determine whether Mcm2-7 encircles dsDNA or ssDNA. The figure shows that nascent CMG still encircles dsDNA in the absence of Sld2 (contrasting with ssDNA in the presence of Sld2), which indicates that Sld2 is required for DNA-strand exclusion from the Mcm2-7 central channel. This is an important discovery but it's not possible from the presented data to assess this result quantitatively. In other words, did the cryoEM dataset show that single CMGE are exclusively seen around dsDNA, when reactions were performed in the absence of Sld2 (indicating that Sld2 is essential under these conditions for DNA-strand exclusion during initiation, despite the presence of Mcm10), or did some proportion of single CMGE complexes encircle ssDNA and the remainder surround dsDNA (suggesting that both Mcm10 and Sld2 can contribute to DNA strand exclusion)? The authors should clarify this point and provide whatever quantification is possible for the new cryoEM data. This is especially important when it comes to the authors' strong assertion that the DNA strand exclusion step represents the 'essential role' of Sld2 (see next point).

This is an important point. We were unable to observe any duplex-DNA interacting sCMGE when all firing factors were used in a dCMGE splitting reaction. We were unable to observe any single-stranded-DNA interacting sCMGE when Sld2 was dropped out.

We revised the last paragraph of the Results section as follows:

"When Sld2 and RPA were present, the 3D structure revealed sCMGE engaged to the fork nexus, with single-stranded DNA running through the MCM central channel (no sCMGE engaged to duplex DNA could be identified in this condition). When Sld2 was dropped out, however, sCMGE was found to be duplex-DNA bound (no sCMGE engaged to single-stranded DNA could be identified)."

2. I think that the revised manuscript will still cause some unnecessary confusion about the 'essential role' of Sld2, when the reader compares the authors' data to previous papers. The authors assert that "Sld2 exerts its essential function after CMGE formation". Assuming that Sld2 is indeed essential for DNA-strand exclusion (see above – the authors need to clarify the

quantification for this point), that doesn't mean that this is the only reason why Sld2 is essential for initiation in vitro and in cells.

In the legend of Figure 7, we now state "Cartoon representation of origin activation, showing that Sld2 plays an essential origin activation function downstream of CMG formation."

In our revised summary paragraph, we now state "While we find that Sld2 promotes GINS recruitment to MCM as expected, it also aids efficient separation of the CMGE dimer, and it is essential for lagging strand ejection from MCM. These findings have direct implications for our understanding of the metazoan Sld2 ortholog, RECQL4, pointing to a replication-fork establishment mechanism conserved across eukaryotes."

The final paragraph of the manuscript downplays the role of Sld2 in recruiting GINS, instead suggesting that Dpb11 plays the GINS recruitment role of metazoan DONSON. This will lead to some confusion, since previous data indicate that the interactions amongst Pol epsilon, Sld2 and Dpb11 jointly support GINS recruitment (likely via contacts on GINS by both Pol epsilon and GINS, which are reinforced by their association with Sld2). For example, Araki's lab showed in Figure 7 of their 2010 Genes Dev paper by Muramatsu et al. that the temperature sensitive lethality caused by mutations in sld2, dpb11 or pol2 (pol epsilon) can be suppressed by over-expression of GINS, consistent with the importance of Sld2-Dpb11-Pol epsilon for GINS recruitment. Metazoan TOPBP1 contacts GINS (like Dpb11) and it remains to be determined whether TOPBP1 also contacts MCM in the preIC, together with DONSON. Did the authors want to highlight that an equivalent interaction of TOPBP1 with MCM (as for Dpb11) would clash with what we know of DONSON's interaction with MCM (suggesting that yeast Dpb11 is really doing something that metazoan TOPBP1 might not)?

We thank the reviewer for this interesting point. We prefer not to add further speculation to our discussion section.

To help the reader to interpret the authors' exciting new data alongside previous studies, perhaps they could consider some minor changes to the text, such as:

- Instead of "Sld2 exerts its essential function after CMGE formation" the authors could say "Sld2 plays an essential role after CMGE formation".

Agreed. Modified as described above.

- Instead of "we conclude that the essential function of Sld2 during origin activation must be performed downstream of dCMGE assembly" the authors could say "we conclude that Sld2 plays an essential role during origin activation that is downstream of dCMGE assembly."

One sentence in last paragraph of the results has been modified as follows. "Given that we can reproduce the observation with the same purified proteins used in our nsEM experiments (*not shown*), we conclude that an essential function of Sld2 during origin activation must be performed downstream of dCMGE assembly."

- Instead of saying “However, Sld2 is not required for making a stable dCMGE complex. Its essential role is instead enacted after holohelicase assembly.” the authors could say “However, Sld2 is not absolutely required for making a stable dCMGE complex. In contrast, it plays an essential role after holohelicase assembly.”

A passage in the discussion section has been modified as follows: “However, Sld2 is not absolutely required for making a stable dCMGE complex. Instead, it plays an essential role after holohelicase assembly.”

3. Given the important new insight into Sld2 function, might a revised title for the manuscript be helpful?

We feel the title of the manuscript is appropriate as it describes the biogenesis of the CMGE complex, which is the active form of the replicative helicase at the replication fork. Such biogenesis implies splitting of a double CMGE into single CMGEs and ejection of the lagging strand.

4. It’s only a minor point, but I couldn’t access Ext. Data Table 2 (the file was a duplicate of Ext. Data Table 1).

Apologies for the oversight. Now fixed.
